# In Silico Analysis of P450s and Their Role in Secondary Metabolism in the Bacterial Class *Gammaproteobacteria*

**DOI:** 10.3390/molecules26061538

**Published:** 2021-03-11

**Authors:** Ntombizethu Nokuphiwa Msomi, Tiara Padayachee, Nomfundo Nzuza, Puleng Rosinah Syed, Justyna Dorota Kryś, Wanping Chen, Dominik Gront, David R. Nelson, Khajamohiddin Syed

**Affiliations:** 1 Department of Biochemistry and Microbiology, Faculty of Science and Agriculture, University of Zululand, KwaDlangezwa 3886, South Africa; msomizethu085@gmail.com (N.N.M.); teez07padayachee@gmail.com (T.P.); nomfundonzuza11@gmail.com (N.N.); 2 Department of Pharmaceutical Chemistry, College of Health Sciences, University of KwaZulu-Natal, Durban 4000, South Africa; prosinah@gmail.com; 3 Biological and Chemical Research Center, Faculty of Chemistry, University of Warsaw, Pasteura 1, 02-093 Warsaw, Poland; juchxd@gmail.com; 4 Department of Molecular Microbiology and Genetics, University of Göttingen, 37077 Göttingen, Germany; chenwanping1@foxmail.com; 5 Department of Microbiology, Immunology and Biochemistry, University of Tennessee Health Science Center, Memphis, TN 38163, USA

**Keywords:** cytochrome P450 monooxygenases, *Gammaproteobacteria*, genome data mining, lifestyle, secondary metabolism, biosynthetic gene clusters, *Firmicutes*, *Streptomyces*, *Mycobacterium*, *Cyanobacteria*

## Abstract

The impact of lifestyle on shaping the genome content of an organism is a well-known phenomenon and cytochrome P450 enzymes (CYPs/P450s), heme-thiolate proteins that are ubiquitously present in organisms, are no exception. Recent studies focusing on a few bacterial species such as *Streptomyces*, *Mycobacterium*, *Cyanobacteria* and *Firmicutes* revealed that the impact of lifestyle affected the P450 repertoire in these species. However, this phenomenon needs to be understood in other bacterial species. We therefore performed genome data mining, annotation, phylogenetic analysis of P450s and their role in secondary metabolism in the bacterial class *Gammaproteobacteria*. Genome-wide data mining for P450s in 1261 Gammaproteobacterial species belonging to 161 genera revealed that only 169 species belonging to 41 genera have P450s. A total of 277 P450s found in 169 species grouped into 84 P450 families and 105 P450 subfamilies, where 38 new P450 families were found. Only 18% of P450s were found to be involved in secondary metabolism in Gammaproteobacterial species, as observed in *Firmicutes* as well. The pathogenic or commensal lifestyle of Gammaproteobacterial species influences them to such an extent that they have the lowest number of P450s compared to other bacterial species, indicating the impact of lifestyle on shaping the P450 repertoire. This study is the first report on comprehensive analysis of P450s in *Gammaproteobacteria*.

## 1. Introduction

The phylum *Proteobacteria* is the most metabolically diverse bacterial phylum [1,2], constituting most of the known bacteria of medicinal, industrial, and agricultural significance [1,2]. Based on the 16S rRNA gene sequence and multiprotein phylogenetic analysis, *Proteobacteria* are divided into seven classes: *Alphaproteobacteria*, *Betaproteobacteria*, *Gammaproteobacteria*, *Deltaproteobacteria*, *Epsilonproteobacteria*, *Zetaproteobacteria* and *Acidithiobacillia* [3]. *Proteobacteria* members are well-known human pathogens; especially the class *Gammaproteobacteria* contain bacterial species that are medically, ecologically and scientifically important [1,2]. *Gammaproteobacteria* contain species with different physiology such as anaerobic, microaerophilic, and facultative aerobic, as well as different shapes such as rods, cocci or curve shapes [1]. Most of the bacterial species in this class are commensals with the ability to cause diseases and some of them are strictly pathogenic. Below we provide brief information on well-known pathogens of this class (Table 1).

Because of the pathogenic nature of these species, quite a large number of Gammaproteobacterial species’ genomes have been sequenced and are available for public use at different genome databases, including the Kyoto Encyclopedia of Genes and Genomes (KEGG) [26], the National Center for Biotechnology Information (NCBI) (https://www.ncbi.nlm.nih.gov/genome/microbes/) (accessed on 15 January 2021) and Joint Genome Institute Integrated Microbial Genomes and Microbiomes [27]. Most of these genome-sequencing articles focused on finding traits that play a role in pathogenesis to come up with novel drug targets. However, key enzymes such as cytochrome P450 monooxygenases (CYPs/P450s) have been neglected or not reported in the genome sequencing studies owing to their typical nomenclature criteria that involve identification of key motifs and assigning the family and subfamily based on International P450 Nomenclature rules [28,29,30]. Only a handful of studies reported the presence of a number of P450s in certain groups of *Gammaproteobacteria* [31,32]. P450s are heme-thiolate proteins known to be involved in organisms’ primary and secondary metabolism and are found in species belonging to different biological kingdoms [33], including in non-living entities such as viruses [34]. P450s perform enzymatic reactions in regio- and stereo-specific manners [35,36] and because of this inherent property they have been under the spotlight for the last 57 years to harness their potential [37]. P450s’ enzymatic reactions are essential in assessing the drug toxicity of prodrugs and xenobiotic compounds have been well-established [38]. P450s serve as drug targets against pathogenic microbes [39,40,41]. Well-known examples of P450s targeted are the CYP51 [39] and CYP53 [40] families. P450s have been investigated for various biotechnological, environmental and pharmaceutical applications and recently, their role in the generation of secondary metabolites valuable to humans has been thoroughly reviewed [42,43]. One of the best examples of the role of P450s is antibiotic production, such as in the biosynthesis of the macrolide antibiotic erythromycin and glycopeptide antibiotics [44,45] and in the production of the anticancer drugs taxol and perillyl alcohol [46,47]. Gammaproteobacterial species, especially marine species, are well-known producers of a variety of secondary metabolites [48]. These metabolites include antibacterial, antiviral and anti-tumor compounds (violacein, pentabromopseudilin, alterochromides and thiomarinol) and a variety of siderophores (vibrioferrin, anguibactin, vanchrobactin, vibriobactin, avaroferrin, putrebactin, and bisucaberin) [48]. The synthesis of secondary metabolites such as thiomarinol, the alterochromides, pentabromopseudilin, and violacein compounds has been studied in detail and their synthesis has been linked to the biosynthetic gene clusters [48]. Information on Gammaproteobacterial species producing different secondary metabolites, secondary metabolites’ biological properties and their genetic information has been reviewed [48].

Recent studies from our laboratory focusing on bacterial species revealed that the lifestyle of an organism plays a key role in shaping P450 content vis à vis P450s helping microbes to adapt to ecological niches. However, these studies were limited to few groups of bacterial species belonging to the genera *Streptomyces* [49,50] and *Mycobacterium* [51,52,53] and from the phyla *Cyanobacteria* [54] and *Firmicutes* [55,56]. Furthermore, to date, a comparative analysis of P450s in the Gammaproteobacterial species has not been reported. Thus, this study is aimed at addressing these research gaps by not only performing genome data mining, annotation and phylogenetic analysis of P450s in Gammaproteobacterial species, but also performing comparative analysis with other bacterial species to see the impact of lifestyle shaping the P450 content in this group of bacteria, among others with respect to P450s’ involvement in secondary metabolism.

## 2. Results and Discussion

### 2.1. Only Few Gammaproteobacterial Species Have P450s

**Figure 1:**
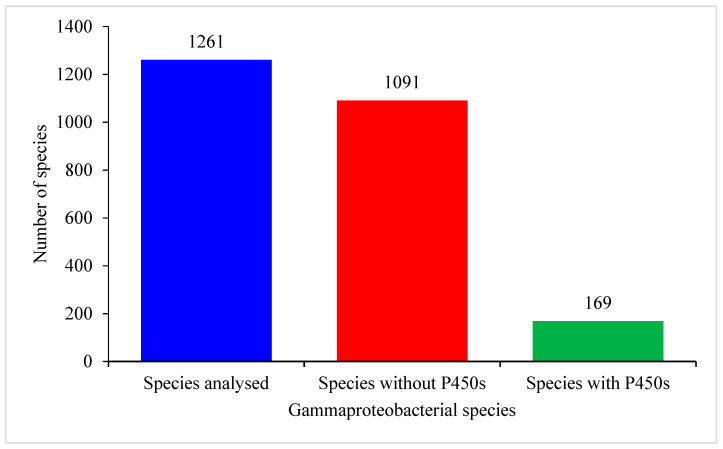
Analysis of P450s in Gammaproteobacterial species. Detailed analysis of the species and P450s is presented in Supplementary Dataset 1.

Genome-wide data mining for P450s in 1261 Gammaproteobacterial species belonging to 161 genera revealed the presence of P450s only in 169 Gammaproteobacterial species, indicating that most of these species do not have P450s in their genomes (Figure 1 and Supplementary Dataset 1; Sheet 1). Analysis of the *Gammaproteobacteria* genera disclosed that of the 161 genera, species belonging to 41 genera have P450s in their genomes (Supplementary Dataset 1; Sheet 1). A point to be noted is that the number of species genomes available in the 120 genera is very low. Sometimes only a single species genome is available and thus future availability of a higher number of species genomes will provide more accurate information on P450s in these genera (Supplementary Dataset 1: Sheet 1). However, a significant number of species belonging to genera such as *Shewanella*, *Aeromonas* and *Haemophilus* were analyzed in this study and no P450s were found, suggesting that species in these genera probably do not have P450s (Supplementary Dataset 1: Sheet 1). Genera-level comparison revealed that at least 50% of *Pseudomonas* and *Xanthomonas* species have P450s. Analysis of P450s in 169 Gammaproteobacterial species revealed the presence of 277 P450s in their genomes (Supplementary Dataset 1: Sheet 2). The P450 count in the Gammaproteobacterial species ranged from a single P450 to six P450s; *Gammaproteobacterium* HdN1 had the highest number of P450s in its genome (Supplementary Dataset 1: Sheet 2). Apart from the complete P450 sequences (277 P450s), one P450 fragment and 10 P450 false-positive hits were also found in some Gammaproteobacterial species (Supplementary Dataset 1: Sheet 2). Comparative analysis of the average number of P450s with *Firmicutes* species, *Streptomyces*, mycobacterial species and cyanobacterial species revealed that Gammaproteobacterial species have the lowest average number of P450s (0.2%) in their genomes (Table 2). Gammaproteobacterial species P450 sequences along with P450 fragment and false positive hit proteins were presented in Supplementary Dataset 2. 

### 2.2. A Few P450 Families Are Expanded in Gammaproteobacterial Species

**Figure 2:**
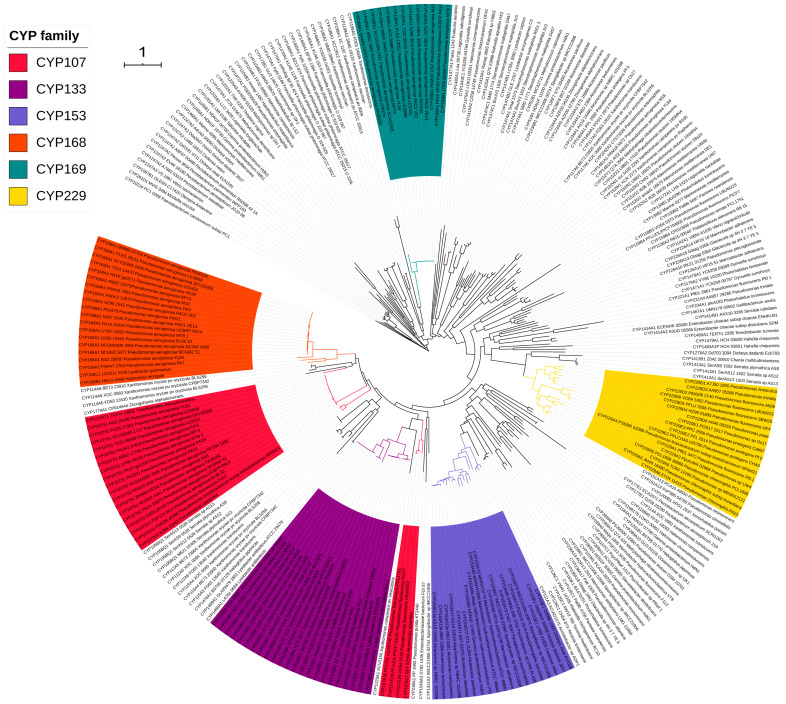
Phylogenetic analysis of Gammaproteobacterial species P450s. P450 families that are expanded in these species were highlighted in different colors and indicated in the figure.

Based on the P450 nomenclature criteria [29,30], 277 P450s of Gammaproteobacterial species can be classified into 84 P450 families and 105 P450 subfamilies (Table 3). Annotation of P450s in this study was further verified by performing phylogenetic analysis (Figure 2). As shown in Figure 2, P450s belonging to the same family grouped together, indicating the correct annotation of P450s. Gammaproteobacterial species P450s identified in this study, along with their protein sequences and species, are presented in Supplementary Dataset 2. The number of P450 families in Gammaproteobacterial species is found to be highest compared to *Firmicutes* species, mycobacterial species and cyanobacterial species, indicating the highest diversity of P450s in Gammaproteobacterial species (Table 2). However, the number of P450 families in Gammaproteobacterial species was found to be lowest compared to *Streptomyces* species (Table 2). In order to understand the P450 diversity in Gammaproteobacterial species, the P450 diversity percentage was calculated by modifying the previously proposed formulae [54,57] to reflect the actual number of species with P450s. The new formula is presented below:

(1) P450 diversity percentage =100×Total number of P450 families÷(Total number of P450s×Number of species with P450s)

Based on the above formulae, Gammaproteobacterial species were found to have the highest P450 diversity percentage compared to *Firmicutes* species, mycobacterial species, cyanobacterial species, and *Streptomyces* species (Table 2). 

**Figure 3:**
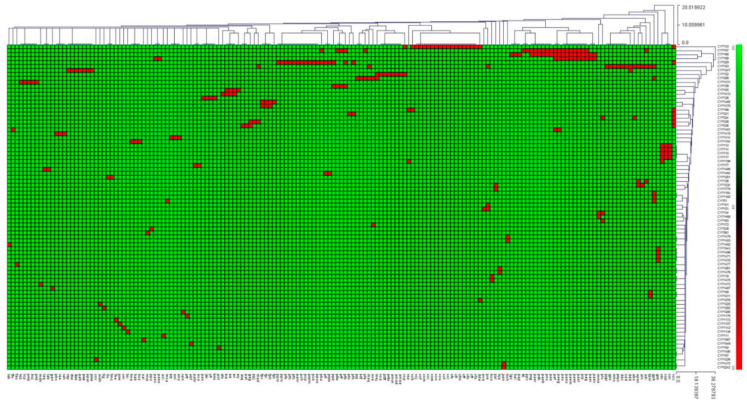
Heat map figure representing the presence or absence of cytochrome P450 families in 169 species of Gammaproteobacterial species. The data have been represented as −3 for family absence (green) and 3 for family presence (red). One-hundred and sixty-nine Gammaproteobacterial species form the horizontal axis and 84 P450 families form the vertical axis.

Among P450 families, the CYP133 has the highest number of member P450s with two-digit members: (23 P450s) contributing to 8.3% of total P450s in Gammaproteobacterial species (Table 3), followed by CYP107 (22 P450s contributing 7.9%), CYP168 (20 P450s contributing 7.2%), CYP153 (19 P450s contributing 6.8%), CYP229 (18 P450s contributing 6.5%) and CYP169 (13 P450s contributing 4,7%) (Table 3). The number of member P450s in the remaining 78 P450 families ranged from one to nine members (Table 3). The CYP133 family was found to be dominant in Gammaproteobacterial species, whereas in other bacterial species P450 families such as CYP107, CYP125 and CYP110 are dominantly present (Table 3). Considering the number of member P450s and the large number of species analyzed in this study, it can safely be said that these P450 families are expanded and not bloomed (a few P450 families with many genes) in Gammaproteobacterial species, which is contrary to other bacterial species where some P450 family blooming was observed [49,50,51,54]. The number of subfamilies in a family ranged from one to five subfamilies; CYP152 has five subfamilies, followed by CYP107 with four subfamilies (Table 3). Interestingly, some kind of P450 subfamily level expansion was observed in Gammaproteobacterial species where subfamily S in the CYP07 family, subfamily B in the CYP133 family, subfamily A in the CYP153 family and subfamily A in the CYP168 family have the highest number of members (Table 3). Contrary to the other bacterial species, analysis of the conservation of P450 families in Gammaproteobacterial species revealed that none of the 84 P450 families were conserved in these species and that none of the P450 families were co-present (Figure 3). The comparative analysis of P450 families in different Gammaproteobacterial species is presented in Supplementary Dataset 1: Sheet 4.

Analysis of P450 families revealed the presence of 38 new P450 families in Gammaproteobacterial species. Eighty-one P450s were found to be part of these 38 new P450 families. The list of new P450 families found in these species is presented in Supplementary Dataset 1: Sheet 5.

### 2.3. A Few P450s Are Involved in Secondary Metabolism in Gammaproteobacterial Species

**Figure 4:**
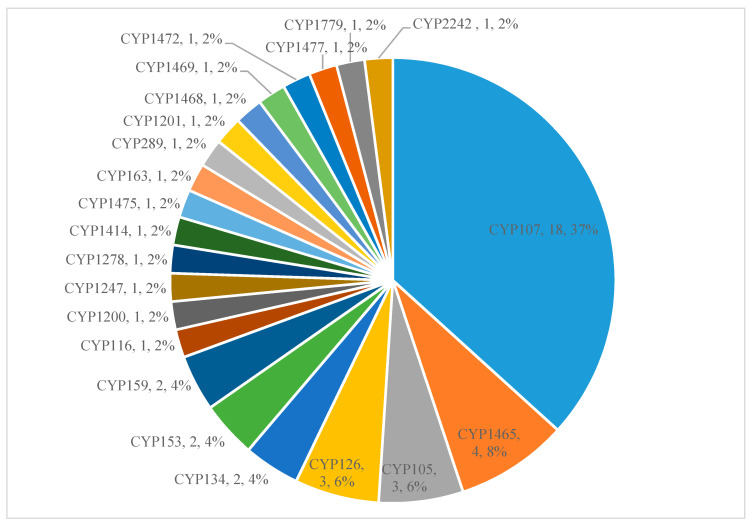
Comparative analysis of P450s involved in secondary metabolism in Gammaproteobacterial species. The P450 family name, number of P450s and the percentage of the total number of P450s that are part of BGCs are presented in the figure. Detailed information on secondary metabolite clusters, species and P450s is presented in Supplementary Dataset 1: Sheet 2.

Analysis of the P450s part of secondary metabolite biosynthetic gene clusters (BGCs) revealed that only a few P450s (18%) are part of these clusters, indicating their involvement in secondary metabolism in Gammaproteobacterial species (Figure 4). The percentage of P450s involved in Gammaproteobacterial species was found to be the same as in *Firmicutes* species (Table 2). Among 277 P450s, only 49 P450s belonging to 22 P450 families were found to be part of a secondary metabolite gene cluster (Figure 4). Among these families, CYP107 P450 family members were dominantly present (37%) in secondary metabolite BGCs (Figure 4). The same phenomenon was observed in *Streptomyces* species [49,50] and *Firmicutes* species [55], where CYP107 P450 family members were dominantly present as part of BGCs. Interestingly, among six P450 families that are expanded in these species, only two P450 family members were found to be part of the clusters (Figure 4). Among these two P450 families, the CYP107 family has 18 members and the CYP153 family has two members involved in secondary metabolism. This suggests that P450 family expansion has no correlation with P450s’ involvement in secondary metabolism in Gammaproteobacterial species, with the exception of the CYP107 family. The analysis of P450 secondary metabolite gene clusters revealed the presence of 12 types of BGCs, where the nonribosomal peptides (NRPS) were dominant (Supplementary Dataset 1: Sheet 2). One interesting point to be noted is that CYP107 family members are associated with the NRPS cluster (Supplementary Dataset 1: Sheet 2). The P450 secondary metabolite gene clusters showed the lowest percentage similarity to known secondary metabolite clusters, suggesting that these gene clusters probably produce novel compounds. Detailed information on P450s, their particular type of cluster and percentage identity to known secondary metabolite gene clusters is presented in Supplementary Dataset 1: Sheet 2). 

### 2.4. Functional Prediction of Gammaproteobacterial P450s

Most of the Gammaproteobacterial P450s are orphans without an assigned biological function. Based on homolog P450s from other organisms, some of the Gammaproteobacterial P450s functions can be predicted. CYP51, also known as sterol 14α-demethylase, is a highly conserved P450 across the phyla and stimulates a key enzymatic reaction that involves the stereo-selective three-step oxidative removal of the 14α-methyl group from the sterol during the synthesis of membrane sterol [39]. CYP102 family members are involved in the hydroxylation of fatty acids [58,59,60]. CYP101 family members are involved in camphor hydroxylation [61]. CYP105, CYP107, and CYP109 have been found to display highly diverse functions [43,62,63]. CYP107 and CYP105 family members are involved in degradation and biotransformation of a large number of xenobiotic and secondary metabolites [63,64]. P450s belonging to the CYP107, CYP109, and CYP134 families were found to hydroxylate different steroids, albeit with different substrate specificities [22]. CYP152 family members were found to be peroxygenases catalyzing the hydroxylation and decarboxylation of fatty acids [65,66,67]. CYP153 members are well-known alkane hydroxylases [68]. CYP226 family members are involved in the catabolism of dehydroabietic acid and abietic acid [69,70]. CYP261 family members were found to have a high affinity to fatty acids [71]. Based on the biosynthetic gene cluster analysis, in this study, we conclude that 49 P450s are involved in the synthesis of secondary metabolites (Supplementary Dataset 1: Sheet 2). However, the functions of these P450s in the synthesis of different secondary metabolites need to be determined. 

### 2.5. Impact of Lifestyle on P450 Repertoire Is Also Evident in Gammaproteobacteria 

It is now well known that lifestyle plays a key role in shaping the genome content of organisms, and P450s are no exception. The impact of lifestyle on P450 profiles in organisms or P450s’ role in the adaptation of organisms to different ecological niches reported in animals [72], plants [73], oomycetes [57] and fungi [28,40,74,75,76,77,78,79] was scrutinized. A number of P450s or a number of members of a P450 family have been found to be present in large numbers in species compared to the pathogenic species or species adapted to a simpler lifestyle where simpler/abundant carbon sources are available for survival. This phenomenon of the impact of lifestyle shaping P450 content has also been observed in bacterial species belonging to the genera *Streptomyces* [49,50], *Mycobacterium* [51,52], and the phyla *Cyanobacteria* [54] and *Firmicutes* [55]. Especially in *Firmicutes,* it has been reported that the pathogenic/commensal lifestyle was influenced to such an extent that some of the species belonging to quite a large number of genera have no P450s in their genomes [55]. The same phenomenon of a low number or no P450s was also observed in Gammaproteobacterial species’ genomes. Most of the bacterial species in the class *Gammaproteobacteria* are commensals with the ability to cause diseases and some of them are strictly pathogenic [1,2] which possibly led to have fewer P450s, as observed for *Firmicutes* [55]. The presence of the lowest number of P450s compared to the large number of species analyzed in the study (Table 2) clearly indicates that these bacterial species adapted to mostly living on simple carbon sources available in the host environment, which led to having fewer P450s in their genomes as observed in other species [55,77]. However, a noticeable difference was that the Gammaproteobacterial species had the highest number of P450 families and subfamilies compared to *Firmicutes* species, and thus the highest P450 diversity percentage compared to other bacterial species (Table 2). Nevertheless, the percentage of P450s involved in BGCs is the same as in *Firmicutes* species, indicating that most of the P450s play a role in primary metabolism. In summary, based on the number of P450s present in Gammaproteobacterial species and in comparison with other bacterial species, it is safe to say that the lifestyle of Gammaproteobacterial species profoundly affect their P450 repertoire, which is the same as what is observed in other organisms, as mentioned earlier. 

## 3. Materials and Methods

### 3.1. Species and Their Genome Database Information

In this study, 1261 Gammaproteobacterial species belonging to 161 genera that are available for public use at the KEGG [80] database were used. Information on genera, species names, species codes and their genome IDs is presented in Supplementary Dataset 1: sheet 1. 

### 3.2. Genome Data Mining and Annotation of P450s

P450 data mining and annotation were carried out following the standard procedure described previously by our laboratory [50,54,55]. Briefly, proteomes of each bacterial species were downloaded from the KEGG and subjected to the NCBI Batch Web CD-Search Tool [81]. The result was analyzed and proteins that belong to the P450 superfamily were selected and searched for the presence of characteristic P450 motifs, EXXR and CXG [82,83]. Proteins that were short in amino acid length and lacking both motifs were regarded as P450 fragments and these P450 fragments were not considered for further analysis. Proteins having both motifs were selected and subjected to BLAST analysis against annotated P450s at the P450 website (http://www.p450.unizulu.ac.za/) (accessed on 15 January 2021). Based on the percentage identity to the named homolog P450s at the P450 website, selected P450s were assigned to specific P450 families and P450 subfamilies, following the International P450 Nomenclature Committee rule [28,29,30] that proteins with >40% identity and >55% identity will be grouped under the same family and subfamily, respectively. P450s with less than 40% identity were assigned to a new P450 family.

### 3.3. Phylogenetic Analysis of P450s

Phylogenetic analysis of P450s was carried out following the protocol previously described by our laboratory [54,55]. Briefly, the P450 protein sequences where aligned using the MAFFT v6.864 program [84], which is available at the Trex web server [85]. For tree inferring and optimization, the Trex web server [85] was used. Then, the server inferred the tree with different algorithms, including maximum likelihood, for the best phylogenetic tree in the least-squares sense. Finally, the best-inferred tree was visualized, colored and generated by the Interactive Tree of Life (iTOL) [86].

### 3.4. Generation of P450 Profile Heat Maps

The generation of the heat map profile was carried out according to the method previously reported by our laboratory [28,54,55]. Gammaproteobacterial P450 family data information was used to show the presence and absence of P450s in this class. The data were presented as positive 3 with the presence of P450 indicated in red and negative 3 indicated in green for the absence of P450. A tab-delimited file containing P450 presence and absence data was loaded into the multi-experiment viewer using a two-color array [87]. Hierarchical clustering using a Euclidean distance metric was used to cluster the data. P450 families formed the vertical axis and Gammaproteobacterial species formed the horizontal axis.

### 3.5. Identification of P450 Part of Secondary Metabolite BGCs

The identification of secondary metabolite BGCs and P450s forming part of the BGCs was carried out following the procedure previously reported by our laboratory [54,55,56]. Briefly, genome ID of Gammaproteobacterial species (Supplementary Dataset 1: sheet 1) having P450s was submitted to anti-SMASH (antibiotics & Secondary Metabolite Analysis Shell) [88] for the identification of secondary metabolite BGCs. Anti-SMASH results were downloaded both in the form of gene cluster sequences and Excel spreadsheets representing species-wise cluster information. P450s that formed part of a specific gene cluster were identified by manual data mining of gene cluster sequences. Standard gene cluster abbreviation terminology available at the anti-SMASH database [88] was maintained in this study.

### 3.6. Data Analysis

All calculations were carried out following the procedure reported previously by our laboratory [54]. The average number of P450s was calculated using the formula: Average number of P450s = Number of P450s/Number of species. The P450 diversity percentage was calculated using the formula presented in the main text of this article. The percentage of P450s that formed part of BGCs was calculated using the formula: Percentage of P450s part of BGCs = 100 × Number of P450s part of BGCs/Total number of P450s present in species.

### 3.7. Comparative Analysis of P450s and Gene Cluster Data

P450s and their BGCs data for *Firmicute* species [55], *Streptomyces* species [49,50], mycobacterial species [49,51] and cyanobacterial species [54] were retrieved from published articles and used for comparative analysis.

## 4. Conclusions

Sustained research into genome sequencing resulted in the sequencing of a large number of species genomes. This gives us an opportunity to look at specific protein families, their distribution and their role in organisms’ physiology. This study is an example of such work, where cytochrome P450 monooxygenases (CYPs/P450s) were analyzed in the bacterial class *Gammaproteobacteria*. The study revealed that species in this class have few P450s in their genomes compared to other bacterial species. Interestingly, despite having a low number of P450s, Gammaproteobacterial species have the highest P450 diversity percentage compared to *Firmicutes* species, *Streptomyces* species, mycobacterial species and cyanobacterial species. The lifestyle of Gammaproteobacterial species, pathogenic or commensalism, had a profound impact on the P450 count in their genomes, leading to having a low number of P450s, as observed in *Firmicutes* species, indicating that the lifestyle of an organism plays a key role in shaping P450 content. Since P450 enzymes follow typical nomenclature criteria, this study will serve as a reference for P450 annotation in the class *Gammaproteobacteria*. Studies are in progress to determine the impact of lifestyle in other bacterial species.

Another interesting result from the study is the identification of secondary metabolite biosynthetic gene clusters that have P450s in the Gammaproteobacterial species. In this post-genomic era, before entering the laboratory one can analyze large numbers of genomes and select the best candidates for the production of unique/novel secondary metabolites. This approach is now gaining momentum, as it saves a tremendous amount of wet-laboratory work, money and laborious practice. In this study, we followed this approach and identified secondary metabolite biosynthetic gene clusters that seem to be novel, as they have the lowest percentage identity to known or characterized secondary metabolite gene clusters, indicating that the compounds synthesized by these gene clusters are novel and possibly have biotechnological values. Future studies include cloning of these novel clusters to enable the synthesis of metabolites and assessment of the biological properties of the synthesized metabolite.

## Figures and Tables

**Table 1:** Information on well-known pathogens of the bacterial class *Gammaproteobacteria*.

Species Name	General Information	Reference(s)
*Citrobacter freundii*	It is a commensal resident of the intestinal tracts of both humans and animals. It causes diarrhea and other infections in humans.	[4]
*Citrobacter koseri*	It is present as part of human flora. It causes meningitis in neonates, infants and immune-compromised people and in adults it causes urinary tract infections.	[5]
*Enterobacter cloacae*	It is a common nosocomial pathogen capable of producing a wide variety of infections, such as pneumonia, urinary tract infections, and septicemia.	[6]
*Enterobacter aerogenes*	It is a commensal of humans and an opportunistic pathogen. It causes respiratory, urinary, blood, or gastrointestinal tract infections.	[7]
*Enterobacter sakasakii*	It is a food-borne pathogen causing a severe form of sepsis, necrotizing enterocolitis and meningitis in neonatal infants.	[8]
*Escherichia coli*	It is commonly found in human flora. Pathogenic *E. coli* can be divided into two groups: extra-intestinal pathogens associated mainly with neonatal meningitis and urinary tract infections in adults and intestinal pathogens causes diarrhea.	[9]
*Escherichia albertii*	It is an emerging human enteropathogen and avian pathogen that causes diarrhea.	[10,11]
*Klebsiella pneumoniae*	It used to be an opportunistic pathogen causing a wide range of infections in immunocompromised patients, but has recently emerged as a pathogen of healthy and immunocompetent people. It causes pneumonias, urinary tract infections, bacteremia, and liver abscesses.	[12,13]
*Klebsiella oxytoca*	It is an emerging pathogen causing a wide range of infections similar to *K. pneumoniae*. *K. oxytoca* causes neonatal infections of the bloodstream, urinary tract, central nervous system, lung, skin, and soft tissues.	[14]
*Proteus mirabilis*	It is a human opportunistic pathogen. It causes infections of the urinary tract, including cystitis and pyelonephritis. It is also found in cases of asymptomatic bacteriuria, particularly in the elderly and patients with type 2 diabetes.	[15,16,17]
*Proteus vulgaris*	It is also an opportunistic pathogen causing infections such as *P. mirabilis*.	[18]
*Salmonella enterica*	This food-borne pathogen causes typhoid and paratyphoid fever in humans.	[19]
*Serratia marcescens*	It is an opportunistic pathogen causing bacteremia/sepsis.	[20]
*Shigella dysenteriae, S. flexneri*, *S. sonnei* and *S. boydii*	*Shigella* spp. are responsible for acute diarrhea and are major contributors to the global diarrheal disease burden.	[21,22]
*Yersinia pestis*	It causes bubonic plague and pneumonia. It is well-known to cause one of the most devastating diseases of human history, the black death.	[23]
*Yersinia enterocolitica*	It causes acute diarrhea, mesenteric adenitis, terminal ileitis, and pseudo-appendicitis.	[24]
*Yersinia pseudotuberculosis*	It causes mesenteric lymphadenitis, diarrhea, and septicemia in humans.	[25]

**Table 2:** Comparative analysis of key features of P450s and their association with secondary metabolism between Gammaproteobacterial species and different bacterial species. BGCs: biosynthetic gene clusters.

	Gammaproteobacterial Species	*Firmicutes* Species	*Streptomyces* Species	Mycobacterial Species	Cyanobacterial Species
Total no. of species analyzed	1261	972	203	60	114
No of Species have P450s	169	229	203	60	114
No. of P450s	277	712	5460	1784	341
No. of families	84	14	253	77	36
No. of subfamilies	105	53	698	132	79
Dominant P450 family	CYP133	CYP107	CYP107	CYP125	CYP110
Average no. of P450s	0.2	1	27	30	3
P450 diversity percentage	0.18	0.008	0.02	0.07	0.09
No. of P450s part of BGCs	49	126	1231	204	27
Percentage of P450s part of BGCs	18	18	23	11	8
Reference(s)	This work	[55,56]	[49,50]	[49,51]	[54]

**Table 3:** Comparative analysis of P450 families and subfamilies in Gammaproteobacterial species.

Family	Subfamily	P450 Count	Percentage Contribution
CYP101	M	1	0.36
CYP102	J	1	0.36
CYP1043	C	1	0.36
CYP1049	A	1	0.36
CYP105	BQ	4	1.44
CYP107	DJ	3	7.94
	E	4	
	HT	1	
	S	14	
CYP108	R	1	0.36
CYP1097	B	1	0.36
CYP1104	B	3	1.08
CYP111	A	1	0.36
CYP112	A	3	1.08
CYP1138	A	1	0.36
CYP1142	A	1	0.36
CYP114	A	4	1.44
CYP1157	C	1	0.36
CYP115	A	3	1.08
CYP1164	A	1	0.36
CYP1165	A	1	0.72
	B	1	
CYP116	B	2	0.72
CYP1172	A	1	0.36
CYP1179	A	1	0.36
CYP117	A	3	1.08
CYP1199	A	2	0.72
CYP1200	B	1	0.36
CYP1201	A	1	0.72
	B	1	
CYP1202	A	1	0.36
CYP1225	A	1	0.36
CYP1234	A	5	2.17
	B	1	
CYP1247	A	9	3.25
CYP1261	B	1	0.36
CYP126	C	4	1.44
CYP1278	A	1	0.36
CYP1311	A	1	0.36
CYP133	B	22	8.30
	D	1	
CYP134	B	2	0.72
CYP136	F	2	0.72
CYP1413	A	3	1.44
	B	1	
CYP1414	A	1	0.72
	B	1	
CYP1415	A	3	1.08
CYP1464	A	2	0.72
CYP1465	A	4	1.44
CYP1466	A	1	0.36
CYP1467	A	1	0.36
CYP1468	A	1	0.36
CYP1469	A	2	0.72
CYP1470	A	3	1.08
CYP1471	A	1	0.36
CYP1472	A	1	0.36
CYP1473	A	1	0.36
CYP1474	A	3	2.53
	B	2	
	C	2	
CYP1475	A	1	0.36
CYP1476	A	1	0.36
CYP1477	A	1	0.36
CYP1478	A	1	0.36
CYP1479	A	1	0.36
CYP1480	A	1	0.36
CYP1481	A	2	0.72
CYP1482	A	1	0.36
CYP1483	A	1	0.36
CYP151	A	2	0.72
CYP152	G	3	3.25
	R	2	
	W	2	
	X	1	
	Y	1	
CYP153	A	16	6.86
	E	3	
CYP159	B	3	1.08
CYP168	A	18	7.22
	B	1	
	C	1	
CYP169	A	13	4.69
CYP172	B	1	0.36
CYP177	E	2	0.72
CYP198	A	3	1.08
CYP221	A	2	0.72
CYP226	A	5	1.81
CYP229	A	7	6.50
	D	7	
	E	4	
CYP234	A	1	0.36
CYP236	A	3	1.08
CYP238	A	1	0.36
CYP261	D	1	0.36
CYP289	A	6	2.53
	D	1	
CYP51	B	2	0.72
CYP107	S	1	0.36
CYP1229	A	1	0.36
CYP1414	A	1	0.36
CYP2242	A	1	0.36
CYP159	B	1	0.36
CYP163	K	1	0.36
CYP1779	A	1	0.36

## Data Availability

Not applicable.

## Supplementary Materials

The following are available online, Supplementary Dataset 1: Sheet1: Information on species used in the study; Sheet 2: Secondary metabolite biosynthetic gene clusters analysis in Gammaproteobacterial species; Sheet 3: P450 profile heat map information; Sheet 4: Comparative analysis of P450 families in Gammaproteobacterial species; Sheet 5: New P450 families and their count in the bacterial class *Gammaproteobacteria*; Supplementary Dataset 2: Gammaproteobacterial species P450 sequences identified in this study. Each P450 sequence is presented with its assigned name, protein code (in parenthesis) and species name. Full-length P450 sequences along with P450-fragment and false positive hit proteins were presented.

## Sample Availability

Samples of the compounds are not available from the authors.
