# Supplementary material for: In Silico Analysis of P450s and Their Role in Secondary Metabolism in the Bacterial Class Gammaproteobacteria
Source: Molecules. 2021 Mar 11;26(6):1538. doi: 10.3390/molecules26061538 (PMC7998510; doi:10.3390/molecules26061538)
Supplement: Supplementary file 1 [file molecules-26-01538-s001.zip › Supplementary Datasets/Supplementary Dataset 2.docx]

Type of the Paper (Article)

*In silico* analysis of P450s and their role in secondary metabolism in the bacterial class *Gammaproteobacteria*

Ntombizethu Nokuphiwa Msomi ^1^, Tiara Padayachee ^1^, Nomfundo Nzuza ^1^, Puleng Rosinah Syed ^2^, Mbalenhle Sizamile Mfeka ^3^, Wanping Chen ^4^, Thandeka Khoza ^3*^, David R Nelson ^5^*, Khajamohiddin Syed ^1^*

^1^ Department of Biochemistry and Microbiology, Faculty of Science and Agriculture, University of Zululand, KwaDlangezwa 3886, South Africa; msomizethu085@gmail.com (N.N.M.); teez07padayachee@gmail.com (T.P.); nomfundonzuza11@gmail.com (N.N.); khajamohiddinsyed@gmail.com (K.S.)

^2^ Department of Pharmaceutical Chemistry, College of Health Sciences, University of KwaZulu-Natal, Durban 4000, South Africa; prosinah@gmail.com (P.R.S.)

^3^ Department of Biochemistry, School of Life Sciences, University of KwaZulu-Natal (Pietermaritzburg Campus), Scottsville, Pietermaritzburg, KwaZulu-Natal, 3209, South Africa; smmfeka850@gmail.com (M.S.M.); KhozaT1@ukzn.ac.za (T.K.)

^4^ Department of Molecular Microbiology and Genetics, University of Göttingen, Göttingen 37077, Germany; chenwanping1@foxmail.com (W.C.)

^5^ Department of Microbiology, Immunology and Biochemistry, University of Tennessee Health Science Center, Memphis, TN 38163, USA; dnelson@uthsc.edu (D.R.N.)

***** Correspondence: KhozaT1@ukzn.ac.za (T.K.), dnelson@uthsc.edu (D.R.N.) and khajamohiddinsyed@gmail.com (K.S.)

**Supplementary Dataset 2:** Gammaproteobacterial species P450 sequences identified in this study. Each P450 sequence is presented with its assigned name, protein code (in parenthesis) and species name. Full-length P450 sequences along with P450-fragment and false positive hit proteins were presented.

**P450s**

>CYP102J4(PC1_0268)Paectobacterium carotovorum subsp. carotovorum PC1

MSGKTAVPQPPIKPVIGNLADVDPRNSIDSLMKLAKTYGPFFKMRIFSDEFYVASSQELVNELSDESFFEKKLSAELLELRYLGGDGLFTAHTHEPNWGKAHRILMPALGPLGVRSMFDKMLDISEQMFLRWERFGPDVDIDVADNMTRLTLDTIALCGFDYRFNSFYRDDLLPFVKAIVGSLKEAGLRVRRPGIVNKLMIPSTRQYRTDKALMYSVVEQLIAARKMDPKASEKNDLLNRMLNGVDPQTGEKLSDENIAHQMLTFLVAGHETTSGMLSFTVYFLLKNPNVLNKARAIVDEVLGDEIPRIEHLAQLRYLEQILMESLRMWPTAGGHVVSPTQDTILAGKYPLTPKDSIVILQPQLHRDVKAWGDDANLFKPERFGPDNAENLLPNSWQPFGSGKRACIGRMFAMQEAQLVLAMMLQRFDFELSDPSYELKIVEHLTIKPDNLKIRIRVRKPSKTLARGVTPKETSNKMAVMPVAKAQHHQDLIPLLVLHGGNTGSSEAFANRIAADAQRYGFASTLAALDDYAEKLPQTGALIVITASYEGMPPNNARHFVPWVEGLADDALSGLKFSVFGCGNLQWVRTYQAIPKRVDLALEHAGGERIHERGVADSGGDFFGNFDDWYTKLWPALTTAFGRQGAVAEDATELELEFVHTDRVSTLQIPDMGRGVVVENRELVDMTSPFARSKRHIEIRLPEGMAYRAGDYLAVLPRNSDDQIDRVLRRFGLSPDMMLVINQAPDVTGLPIGQPISCAELLGNYVELSQPATRAQVAALAAATRCPPEKMELEKLATEHYENDVLAPRLSVLDLLYRFQSCPTDFRRYLTMLPSIKARQYSISSSPAWKPDHVTLTVAVVDSPALSGIGRYKGVASNYLASLKPDDRIAIVVRPSSPFFHLPDDPSIPIILIGAGSGIAPFRGFLQERALQQAAGVRVGPALLFFGTGHPDVDYLYRDELAAWEKSHIVTVLPAFSHQPDGEVTFVQHRVWADRERIKTLFCNGGSLFVCGDGLRMVPEVRDMLLRIYREATGSNETDAMLWADKLEREQGRYVVDMFI

>CYP105BQ1(SerAS9_0528)Serratia plymuthica AS9

MMLSSDANATPHLECLPTRRTCPFNPPEEYRFIRQECPVVRVKTPRGDFAWLVTRHQDVKKALSDKRLSSDPRSAGFPTYISGEVPPPPGFFLQLDAPDHTRLRKAVTEEFLNAHVEKLKPQMTAIIQRQLDELLSMQPPVDFVKAFAIPASAKIICELLGTPVEDHPFVQSRTDTVLDRSSPPELAEQAAIELMGYFDRIVTEKERAPGDDLLGRLIHKAQQAHQPSHEEIVGLAALLLLSAYDTMALAMGLGVVTLLNNPAQLQAFLADIDLGNDLVYELVRYLTINHAGLPRAALEDTEIGGQQIKAGEGVLIMLSSANRDEEIFEQPDVFNLHRREKTQLGFGHGIHKCLGMHFARAELTIAFHAIFTRIPSLAIAVPQETLTYRDEMVLYGLKALPVSWQPA

>CYP105BQ1(M621_02405)Serratia plymuthica S13

MMLSSDANATPHLECLPTRRTCPFNPPEEYRFIRQECPVVRVKTPRGDFAWLVTRHQDVKKALSDKRLSSDPRSAGFPTYISGEVPPPPGFFLQLDAPDHTRLRKAVTEEFLNAHVEKLKPQMTAIIQRQLDELLSMQPPVDFVKAFAIPASAKIICELLGTPVEDHPFVQSRTDTVLDRSSPPELAEQAAIELMGYFDRIVTEKERAPGDDLLGRLIHKAQQEHQPSHEEIVGLAALLLLSAYDTMALAMGLGVVTLLNNPAQLQAFLADIDLGNDLVYELVRYLTINHAGLPRAALEDTEIGGQQIKAGEGVLIMLSSANRDEEIFEQPDVFNLHRREKTQLGFGHGIHKCLGMHFARAELTIAFHAIFTRIPSLAIAVPQETLTYRDEMVLYGLKALPVSWQPA

>CYP105BQ1(SerAS12_0528)Serratia sp. AS12

MMLSSDANATPHLECLPTRRTCPFNPPEEYRFIRQECPVVRVKTPRGDFAWLVTRHQDVKKALSDKRLSSDPRSAGFPTYISGEVPPPPGFFLQLDAPDHTRLRKAVTEEFLNAHVEKLKPQMTAIIQRQLDELLSMQPPVDFVKAFAIPASAKIICELLGTPVEDHPFVQSRTDTVLDRSSPPELAEQAAIELMGYFDRIVTEKERAPGDDLLGRLIHKAQQAHQPSHEEIVGLAALLLLSAYDTMALAMGLGVVTLLNNPAQLQAFLADIDLGNDLVYELVRYLTINHAGLPRAALEDTEIGGQQIKAGEGVLIMLSSANRDEEIFEQPDVFNLHRREKTQLGFGHGIHKCLGMHFARAELTIAFHAIFTRIPSLAIAVPQETLTYRDEMVLYGLKALPVSWQPA

>CYP105BQ1(SerAS13_0528)Serratia sp. AS13

MMLSSDANATPHLECLPTRRTCPFNPPEEYRFIRQECPVVRVKTPRGDFAWLVTRHQDVKKALSDKRLSSDPRSAGFPTYISGEVPPPPGFFLQLDAPDHTRLRKAVTEEFLNAHVEKLKPQMTAIIQRQLDELLSMQPPVDFVKAFAIPASAKIICELLGTPVEDHPFVQSRTDTVLDRSSPPELAEQAAIELMGYFDRIVTEKERAPGDDLLGRLIHKAQQAHQPSHEEIVGLAALLLLSAYDTMALAMGLGVVTLLNNPAQLQAFLADIDLGNDLVYELVRYLTINHAGLPRAALEDTEIGGQQIKAGEGVLIMLSSANRDEEIFEQPDVFNLHRREKTQLGFGHGIHKCLGMHFARAELTIAFHAIFTRIPSLAIAVPQETLTYRDEMVLYGLKALPVSWQPA

>CYP126C1(VW41_14105)Klebsiella michiganensis RC10

MSEAIINSEQAAQSWPLPDVDLTNLDLFSRGFPHQVFTDLRSHHGALFHPRTALTPDGEGFWVFTRYHDIAAIAKDNDTFSSAGGGDRQGGGTMIEDLPREMGPGSVINMMDDPRHKALRRLIGPALTNARVAAMEDILFAAAESAVQAALQQERVDFVSAIAAELPLFAIASLVGIPQDDRHQIFAWINAVLDYSDRQLGETSISSQQGMKNFMAYGHKFVEEKRQNPGSDIVSLAVTGELPKGLGKLTPLEQLMVFSVVMVAGLETTRNAIAGGILAFIHHPEQWLRLQQDGGLMNSALDEILRWTSPTPYNRRTATRDVIIGDRLIRRGEKVTLWWASANRDDAYYEQPFTFDIGRQKNLHLAFGGGGHSCLGAQLARLEMRVILHHLLEQVHSFSLDGEVNWVRSNKHTGIRSMPVRFVKRD

>CYP126C1(AW19_789)Yersinia frederiksenii

MSEAIINSEQAAQLWPLPDVDLTNLDLFSRGFPHQVFTDLRRHRGALFHPRTALTPDGEGFWVFTRYHDIAAIAKDNDTFSSAGGGDRQGGGTMIEDLPREMGPGSVINMMDDPRHKALRRLIAPAITNARVAAMEDVLFAAAGSAVQAALQQERVDFVSAIAAELPLFAIASLVGIPHDDRHQIFAWINAVLDYSDRQLGETSISSQQGMQNFMAYGHKFVEEKRQNPGSDIVSLAVTGELAKGLGKLTPLEQLMVFSVVMVAGLETTRNAIAGGILAFIHHPEQWLRLQQDRGLMNSALDEILRWTSPTPYNRRTATRDVIIGDRLIRRGEKVTLWWASANRDDAYYEQPFAFDIGRQKNQHMAFGGGGHSCLGAQLARLEMRVILHHLLEQVDGFRLDGDVNWVRSNKHTGIRSMPVRFVKR

>CYP126C1(CH54_873)Yersinia kristensenii

MSEAIINSEQAAQLWPLPDVDLTNLDLFSRGFPHQVFTDLRRHRGALFHPRTALTPDGEGFWVFTRYHDIAAIAKDNDTFSSAGGGDRQGGGTMIEDLPREMGPGSVINMMDDPRHKALRRLIAPAITNARVAAMEDVLFAAAGSAVQAALQQERVDFVSAIAAELPLFAIASLVGIPHDDRHQIFAWINAVLDYSDRQLGETSISSQQGMQNFMAYGHKFVEEKRQNPGSDIVSLAVTGELAKGLGKLTPLEQLMVFSVVMVAGLETTRNAIAGGILAFIHHPEQWLRLQQDRGLMNSALDEILRWTSPTPYNRRTATRDVIIGDRLIRRGEKVTLWWASANRDDAYYEQPFAFDIGRQKNQHMAFGGGGHSCLGAQLARLEMRVILHHLLEQVDGFRLDGDVNWVRSNKHTGIRSMPVRFVKRY

>CYP126C1(Pat9b_4758)Pantoea rwandensis

MSEAIINSEQGAQFWPLPEVDLTNLDLFSHGFPHQVFTELRRHNGALFHPRTALTPDGEGFWVFTRYHDIAAIAKDNDTFSSAGGGDRQGGGTMIEDLPREMGPGSVINMMDDPRHKALRRLIGPAITNARVAAMEEVLSAAAGSAVQAALQQESVDFVSAIAAELPLFAIANLVGIPHDDRHQIFAWINAVLDYSDRQLGETSVSSQQGMKNFMAYGHKFVEEKRQNPGGDIVSLAVTGELPKGLGKLTALEQLMVFSVVMVAGLETTRNAIAGGILAFIQHPEQWQRLQQDGSLMNSALDEILRWTSPTPYNRRTATRDVVIGDRLIRRGEKVTLWWASANRDDAYYEQPFAFDIGRQKNLHLAFGGGGHSCLGAQLARLEMRVVLRHLLQQVNGFMLEGEVNWVRSNKHTGIRKMPVRFVKR

>CYP1278A2(Dd703_3094)Dickeya dadantii Ech703

MSDLLNPLSAIQHDSPWEYYARLTRETPVYFDRDLKLWVVSDAGSTDAVLSSPVLQVRPVSQPVPPGLVGQPAGDVFGQLVRMREGEYQQQLKAVIIRALSTVDSAHVSQLARQLAQQALSDGEEINRWMFSVPAAVVATLCGFLPRDVPEVVALIAEFVLCIPAAASPEHQQRASLAATSLLERFFAQIAQAPQGALLTELLQCASEEGWSQRAPLIANAIGFLSQTYDATASLAGNTLLTAQRYPELWLRRPADAFIDETARYTSPIQNTRRFAAHELTLLGQRIAPDDTILLLLAAANRDPARFAQPDVFQPQRNTQAYFSFSGGRHRCPGAAVARAITHGMVSALDAHMPDWHQRLHKRRYLPSGNARIPEFSLSEIN

>CYP133D1(Dd703_1892)Dickeya dadantii Ech703

MQLHELMNPQYSDQPFLLYRKLHALGPLIQAGDHVMISGSHAIIEALLNDRRVGKNYMESVRLRFGDDVAERPVFKGISKMFLVMNPPEHTRLRGLVMKSFSSKEIQLIRDTAIMTANVLVDTFIARGRCDLAKEFAFPFPARIICRMLGVPESDAQAFSNAASTLVKVFDPQITEQDLLSAGEAFTALYNYFSALIMDRQKYAGDDLVSLFLRSENNGDRLQHDEIIANVILLFIAGHETTSNMICNAVLALHDNPMELELLRQNRALIPAAVTECLRYDSSVQMIYRIAQDDIDVQGHIIAQGTRFFLILGAANHDPAVFSAPDVLNIRRREGRALSFGGGIHHCLGYRLALAEMEIALEVLLTRLPEMRLLKSGMERNHRANLRGVSALPVIW

>CYP134B1(plu0296)Photorhabdus luminescens

MAKLSSFNIHDPKFIKNPYDFYDILHKQDLVYFEQSQNSYFIGKYEDVDAILKSSIFNTKPLTALAEPVMGDRVLAQMEGEEHACKRKFIMQGLSRDYFNRYYEPMIRKITEDLLQPYMEKGNIDIVNDFGRDYAVLVTLSILGLPSDNYRDIAEWHKGIASFITQFDQTELEKMHSLECSQKLIRLLKPIIDQRRRNPSKDIISIFCQDTAMSMSEITALCLNILLAATEPADKILAMMLNHLISNPSMLDVVLKDRSLVRDAFEETLRLTSPVQLIPREASEDVTISGIDIPKGAVVFCMIGAANRDPSVFHKPNEFDLYRRKNTTSPQKANRKRHLAFGAGTHACAAAAFSLSQLEVSSNIILDLLHNLRFADHYHYQETGVYTRGPSKLLLSFDPIASSAIKE

>CYP153E1(Dda3937_03358)Dickeya dadantii 3937

MKMTKQLQDVFEDIDAVPLEKINPASRDRFINAAELPMFERLRREDPVHFTPESEFGPYWSLTLWEDIRAVGNNYRDFTSTQNIDLKSIEEKIKLEAALQALGHERRKNVGFITMDPPEHTKHRKAVTPAVGPSSLAQMEPIMRERAGIILDSLPIGEPFDWVDLVSKELTATVLATLLDFPFEERRKLTFWSDLLMFEPGHGLVKSWEQKAEETVKCYQVFEALWEKRRNGPPSYDLISMLAHHPDTRDMTLEQFRGTIVLLIIGGNDTTRNTISSSLYLLDKYPEEFAKLKANPKLVMPMISETLRFHPPVNFMSRVATRDVEIRGKNIKEGDRVVMWYTSGNRDASAIEDPDTFSIDRERARRHLSFGVGVHACIGSRVAEMQLTVIWEEILKRFSRIEVLEEPERSYSNFLHGFENLKVIIPKA

>CYP153E1(LH89_21620)Cedecea neteri

MTKQLQDVVEDVNAIPLEKINPARRDRFVNATELPVFERLRREDPVHFTPESEFGPYWSVTLWDDIRTVGNNYHDFTSTQNIDLKSLEEKEKLETAMQALGHERRKNVGFITMDPPEHTKHRKAAAPSVAPSSLAQMAPIMRERAGIILDGLPIGEPFDWVDRVSKEMTATVLATLLDFPFEERRKLTYWSDLLMFEPGHGPVTSWEQKAEETIKCYQVFEEMWEKRRNAPPSYDLISILAQHPDTRDMTLEQYRGTIVLLIIGGNDTTRNTISGSLYLLDKYPAEFAKLKANPKLVMPMISETLRFHPPVNFMSRVATRDVEIRGKNIQKGDRVVMWYASGNRDASAIEEPDTFSIDRERARRHLSFGVGIHACIGSRLAEMQLTIIWEEILKRFSRIEVLEEPERIYSNFLHGFENMKVIIHKE

>CYP153E1(Dd586_1369)Dickeya zeae Ech586

MTKQLQDVVEDVNAIPLDKINPARRDRFANGTELAVFERLRGEDPVHFTPESEFGPYWSVTLWDDIRTVGSNHRDFTSTQNIDLKTLEEKDRLETALQALGHERRKNVGFITMDPPEHTKHRKAVTPAVAPSSLSKMAPVMRERAGMIIDNLPIGEPFDWVDRVSKEMTATVLATLLDFPFEDRRKLTYWSDVLMFEPGHGPVTSWEQKAEETVKCYQVFEELWEKRRNAPPSYDLISVLAHHPDTRDMTLEQYRGTIVLLIIGGNDTTRNTISSSLYLLNKYPQEFAKLKADPKLVMPMVSETLRFYPPVNFMSRVANRDVELNGKTIKAGDRVVMWYASGNRDGSVIDEPDVFNIDRERSRRHLSFGVGIHTCIGSRLAEMQLTIVWEEILKRFSRIDVLEEPERNYSNFLHGFDSLNVIIHKD

>CYP159B2(Pat9b_5697)Pantoea rwandensis

MSTVHSKSEFCLNHCNHLPPTPLSIPDINLPFAAPSPRSDSDQLRANALAWAQRYCLIGRRGAHRLSTTPLLELGVALCGQAPGQRAEILVCWYLWVLILDDRIDDGPWAENGTLDRFITEVQAVTENNSADPSRFDDPMLVALADDLWPRTKDLGSERWRHQLVQNLIQHLRAQAMLVQMRETAVTVTLDEYLPLRRDSFGALFFFDLIDGAEMLDPYSHAADIEWWNKLREYCADIIAWTNDIHSIAKDVVCGERFNLVSILADTTASDWPAAIISARQMVNTAVEEFTVLAAQQTHCPPSAAIDPDRLRQVVKAAGDWHQTVSRYHLHPDNAATLTQRQVDLKRIPPTLKSREFEIDPYPLYARLRTEMPIVYDEPTDVWLVSRHADVKAALTHPGASNNNYSWQIGPLLGHTIVSMDGCEHAQHRALLNPLFRSKALAVFKSSITSVTRDLLANMQGRTQVDLVADFAATLPVQVMAHALGLPAETAEEVAKLKSWCAVGFAYLGNYRQEPALLSGGLSNRDRFYDFIQPHIDARRAEPKGDLISQLLAAKIDGQPLSEAFIRAYTAILMTAGSVTSVATLTNLIVNLLNQPGVKEAVMAKPELMDNALNETLRRNPPMQLVLREAREALLLPSGTIPAGATLACLIGSANRDPSRFSDPDRFDILRPDQTTSHFAFGAGRHFCLGALLARMEITTGASMLLQAFPNVDWAPGFQPIERGFLKRSFDRLEVTL

>CYP177E1(ECA2071)Pectobacterium atrosepticum SCRI1043

MPTKTQGRPLMFAHPERQDKYFPWADRLFRVNPYPWYDKVRAEHPVYRMENGEIVLTRYHDVMTWLKAPLGISNFGNGPWNNFDNTVLNCDPPEHTTLRRHSNKWFTPKLVNQYVTIATELAENALDRYSDGSVMDAFYELAVVPPHATMCRALGVPEDDAGLIYRHFLTCTDALGHGVGRDDTEKASQSFDYLFERCAHYIKEKRSNPNVGPQGLVDDFLKLADEGKLTERAVLETMVLFYGSGSPNPATVIASGLNHFAREPETFELYRTQPEERNAIINELTRLYPAEISMIRYATEDTEIDGIPVTKGTPVRAVIAAANRDPEFFENPHEFNHKRPPETSMNLTFGVGHHACAGQLISRSAVRSVFDAVAKKATRIQIAGEAGIAHTDRVRGYLSLPLRIY

>CYP177E1(GZ59_25290)Pectobacterium atrosepticum 21A

MFAHPERQDKYFPWADRLFRVNPYPWYDKVRAEHPVYRMENGEIVLTRYHDVMTWLKAPLGISNFGNGPWNNFDNTVLNCDPPEHTTLRRHSNKWFTPKLVNQYVTIATELAENALDRYSDGSVMDAFYELAVVPPHATMCRALGVPEDDAGLIYRHFLTCTDALGHGVGRDDTEKASQSFDYLFERCAHYIKEKRSNPNVGPQGLVDDFLKLADEGKLTERAVLETMVLFYGSGSPNPATVIASGLNHFAREPETFELYRTQPEERNAIINELTRLYPAEISMIRYATEDTEIDGIPVTKGTPVRAVIAAANRDPEFFENPHEFNHKRPPETSMNLTFGVGHHACAGQLISRSAVRSVFDAVAKKATRIQIAGEAGIAHTDRVRGYLSLPLRIY

>CYP234A1(plu4183)Photorhabdus luminescens

MMNVLINEYKKKMDSVRLGDPERKGFFYDAKQAIWHCYSYDICSYFLNSDYVTKKKLSIPLEIFSASDQSRVARFILYLNNSLIFNDDKYNTDAVSFIRGKFNEMNFEVIANDLLSPLKQCDLLTAKHLRGVNNLLAASLVGLKASAFFSAHALNVGMFFDGSMSGRAHFVSIAESFIAIYQQVLRQITINGGAEDVIHIEKFVADLSVTFIAAHETTMQLIIATFLYIKSHVITVTENNIKSIVTETYRLSSPVLAVNRVFKERLIYKNSCFNKGDRVLFYTGLANFDATVFDHPYQFQLDREGCPLSFGVGVKKCIGMNIAIHFTCQLITKILSCYQLDDVEIHEVTVGSLAIGCSKFTLKISKK

>CYP1049A3(D782_1435)Enterobacteriaceae bacterium FGI 57

MSHQVTLRFEDGATHFIQCLTGESVADAALRAKIAIPLDCRDGVCGTCKATCESGHFVLGDYVPDALSDDEANAGHVLTCQMRPSSDCVVQIAATSDAVGISSTAFTGRITACEALSPTAITFTAELENRSALRFLPGQYVNIQVPGSTQTRSYSFSSGPSANEVSFLIRNVPQGLMSSYLREQAKPGDAITFLGPMGSFYLRPIERPLLFLAGGTGLAPFLSMLDKIAEEGEVTQPVHLIFGVTHDEDRVELARLEDYARRLPNFSYLCTVASSESSSPHKGYVTQHITASQLNGGDVDIYLCGPPPMVEAVRDWLAAEGVKPRNFYYEKFAGAGQVVQTGEEHISPEDVDDTFDLRLALELGAVQLTMGRLSGAQLLAFRQLAEATAPFVVGKRFSDVTRYAQANHAFHLFLIEASGNAPLITLYKQLAVQDYIGRALRDDIEIVGDIVQQHQDLVSAFEYGDINAAREVIAQHALHSKATMSRALGKKSAPTAVAPTPQPEPARCPFAALAEQPPYSHELSWPQELQPFKVVDDGSQGDPYEHYRWMREHAPVLRCQSATSDVWFLSRYDDVWQAIRNPKLFSSEVVSPPPLTFLTLYDAPDHTRLRKIAQPSFMPLAIEPFAAEIERRAEVLIDALIAKGGGDVVEEFAIPLSIATISAMIDVPNEDEEKMKFWSDETFSYFGRLARNAPGTGTDEQSSMAFFAYLKEAMERLYLSNSQSIGGHIARMWKEGLLSEKEAKELCAFVFIAGHDTTTILVANAFRMFAEHPHLVQRIRENEADADKFVEEVARYRGTVQRVSRMTTEATTVAGVELPKGAVVRLLLSSANRDSRKFADGDTFNIDRDTTGHLGFGNGMHKCLGQPLAKLETLIATRLVARKVSDIALDPAQPIEYVRGNNLTNSGPAHLFVKLR

>CYP1179A2(VY86_10220)Photorhabdus temperate

MEQFSVYGDTYLNDRYSVYERLRNEAPAYFSKEMNSWFITRYEDVSSLLRSNDLITSHLIQEKLDNLKEGESEHFKEIIDIISTWMIYNDRPVHTRLRKFMNQAFMRKELEIIKPEIAKITHTLIQKITDSGCTEIDFVEEIAHPLPAMILCKMLGLDESEAGKFIKWSDDIADFMQNFVVSSVPDKGVSDITKKSMREMFDFLFETIKSRRNIPRNDLISRLANSEKFEDGAMLGDAEIAAQTVHLIFGGHKIPQFMLTNMLHCLITHPEQMEALKQDSSLLDNALMESMRFEGPIQYVTRHASKDIVLHGETIKKYDSVYLFLGAANRDPRAFQYPDMFNIQREEKLNHVAFGGGYHACIGAAFVQMELNVILEGLFGAFKDIQSNYNIQSPDWSHNPTFHGIKSMSIKVK

>CYP1200B1 (XPG1_2637)Xenorhabdus poinarii

MNTTAFAWPSVATIPNYNCTPGYEVLEPASYQRAGKIKLLSGHEAWHVINYHDVKKVLTSNTCLRGPSNEPNGPSILPTLTPKDLLLNLDFPHHARMKRFAAKDYSASGLAWLAPHMVDAIETLMQAACRGDSFDLYQDVLDPLVAQINCLLLGIPLVEKEYFRVLSVTVQKANPQKVEDLIEKFTALYQYLFEHVTGERQHADDGLIARFVAMRESATPPLNDEEITAILLGSLLGGDQNTLTVMTKIFYALLCTETLWQQVVDFPETTEQVSDELIRLTNLGTASAFPRICSEAIELSGVTIPAGATIFPDVFLANRDPSVYASPLTIDPFRNGPRHLQFGYGMHHCMGQELAKLEIYTAIKTIARLAPGLRLSDHVLPENFIWNEGIILRRPAQLPVCIIRNKVK

>CYP1247A2(Pecwa_3634)Pectobacterium parmentieri WPP163

MRSIKRLPMPPTRGVLGHVDYLKRSDIHLQMLRWKAQYGRFYRLRLGLTPAVVIADTEWIRTIMKSRPGEFRRISSIESVFQEAGLNGVFSSEGERWENQRKLTEPMFQPAHLKYFYSSLRKVTSRLSNRFTMLAETGETIALVDEFKRYTVDITSLLAFGEDVNTLEQGDNPLSQSLRHLFPIINERCESPLPVWRYIRRARDKQFDASLNLIREYVDGFIYRQRQRIQLNPQLLDAPENMLQVMLAEQQKDGMLKDDDIVANAITLLIAGEDTTANTLTWMSFLLCSAPSVEACVFQECKEVTDSVEAILPWPLPRMPWLTAVMYESMRLKPVAPLLYLEPTKNTVIDDFLIKKGTPLLLMLNASGFDDELFQQPYDFMPERWLERGKAAFSDLQPFGGGPRMCPGRSLALMEIKLGFHALCSGFRVEAQQAASAVTESFAFTMTPSGFCVKLHKREQTQ

>CYP1247A2(A8F97_00480)Pectobacterium parmentieri RNS08.42.1A

MRSIKRLPMPPTRGVLGHVDYLKRSDIHLQMLRWKAQYGRFYRLRLGLTPAVVIADTEWIRTIMKSRPGEFRRISSIESVFQEAGLNGVFSSEGERWENQRKLTEPMFQPAHLKYFYSSLRKVTSRLSNRFTMLAETGETIALVDEFKRYTVDITSLLAFGEDVNTLEQGDNPLSQSLRHLFPIINERCESPLPVWRYIRRARDKQFDASLNLIREYVDGFIYRQRQRIQLNPQLLDAPENMLQVMLAEQQKDGMLKDDDIVANAITLLIAGEDTTANTLTWMSFLLCSAPSVEACVFQECKEVTDSVEAILPWPLPRMPWLTAVMYESMRLKPVAPLLYLEPTKNTVIDDFLIKKGTPLLLMLNASGFDDELFQQPYDFMPERWLERGKAAFSDLQPFGGGPRMCPGRSLALMEIKLGFHALCSGFRVEAQQAASAVTESFAFTMTPSGFCVKLHKREQTQ

>CYP1247A2(A7983_03310)Pectobacterium wasabiae

MRSIKRLPMPPTRGVLGHVDYLKRSDIHLQMLRWKAQYGRFYRLRLGLTSAVVIADTEWIRTIMKSRPDEFRRISSIESVFQEAGLNGVFSSEGERWENQRKLTEPMFQPAHLKYFYPSLRKVTSRLSERFTMLAEAGETIALVDEFKRYTVDITSLLAFGEDVNTLEQGENSLSQSLRHLFPIINERCESPIPVWRYIRRARDKQFDVSLSLIREYLDGFIYRQRQRIQLNPQLMDAPENMLQVMLAEQRNNGALKDEDIMANAITLLIAGEDTTANTLTWMSFLLCSAPSAEECIFQECKEAADGAGAILPWPLPRMPWLTAVMYESMRLKPVAPLLYLEPTKDTVIDDFLIKKGTPLLLMLNASGFDDELFQQPYDFMPERWLERGKAAFSDLQPFGGGPRMCPGRSLALMEIKLGFHALCSGFRVEAQQPASAVTESFAFTMTPSGFRVKLHKREQTQ

>CYP1247A2(EV46_18180)Pectobacterium atrosepticum JG10-08

MRSIKRLPMPPTRGVLGHVDYLKRSDIHLQMLRWKAQYGCFFRLRLGLSSAVVIADTEWIRTIMKSRPDEFRRISSIESVFQEAGLNGVFSSEGERWELQRKLTEPMFQPAHLKYFYSSLRKVTSRLSERFTMLAEAGEAIALVDEFKRYTVDITSLLAFGEDVNTLEQGDNPLSQSLRHLFPIIHERCESPIPVWRYIRRARDKQFDASLNLIREYVDGFISRQRQRIQLNPQLLEAPENMLQVMLAEQQKDGTLKDDDIVANAITLLIAGEDTTANTLTWMSFLLCSAPSVEECVFQECKEAAEGVGAILPWPLPRMAWLTAVMYESMRLKPVAPLLYLEPTRDTVIDDFLIKKGTPLLLMLNASGFNDELFQQPYDFMPERWLERGKAAFSDLQPFGGGPRMCPGRSLALMEIKLGFHALCSGFHVEALQVASAVTESFAFTVTPSGFRVKLHKRELQQ

>CYP1247A2(Dd1591_0711)Dickeya zeae Ech1591

MKSIKRLPTPPTRGLLGHVEYLKRHDVHLQLLRWKERYGPFYRLRLGWKPAMVIADAEWIRTIMKARPDEFRRRSSIESVFQEAGLNGVFSSEGTRWEHQRKLTEPMFQPAHLKYFYPSLRKITARLSERFARLAQTGEVFSLVDEFKRYTVDVTSLLAFGEDINIIEQGENPLSQSLRRMFPVINQRCGSPIPLWRYIRRERDKQFDASLSLIRERLYAFIDHQRERLERNPQLIDAPENMLQIMINEQKKDGALTDEDILANAFTLLLAGEDTTANTLAWMSFLLCTSPAVEEQVVRECRQATEDAEDILPWPLPRMPVITAVMYEAMRLKPVAPLLYLEPVKDTVVADFHIRKGTPLLLMLHASGFEEALFQQPRDFMPERWLERGQASFSDLQPFGGGPRMCPGRSLALMEIKLGFHALCSRFRVEAQQPASEVIESFAFTMTPAGFRVRLHKRP

>CYP1247A2(LH89_16610)Cedecea neteri

MRSIKQLPAPPARSILGHVDYLKRPDIHLQMLQWKERYGLFYRLRLGFTPAMVIADSEWIRTIMKARPDDFRRRSSIESVFQEAGLNGVFSAEGARWMHQRKLTEPMFQPAHLKHFYPSLRKVTARLSERFAKLAETGEVVSLVDEFKRYTVDITSLLAFGEDINILEQGENPLSESLRRLFPVINERCGSPIPLWRYIKMARDKQFDASLKLIHDQLNEFICRQRERIQQSPQLMDAPENMLQIMIAEQQKDGTLTDADILANAFTLLLAGEDTTANTLAWMSFLLCSSPSVEAQVVRECHQATEEVGTGLPWPLPRMPLLTAVMYESMRLKPVAPLLYLEPVKDTVIADFLIKKGTPLLLMLNASGFDEALFHQPGDFRPERWLERGQASFSDLQPFGGGPRMCPGRSLALIEIKQGFHALCSGFRVEAQQPALDVMESFAFTVTPIGFRVRLHKRPQPDIAQHEV

>CYP1247A2(Dda3937_03443)Dickeya dadantii 3937

MSFAIPSFFISSGSLNGCFFAGHLSPEQGRISRMRSINRLPMPPTRGLLGHVHYLKRHDVHLQLLQWKERYGPFYRLRLGLTSAMVIADSEWIRTIMKARPDEFRRRSIIESVFQEAGLNGVFSSEGARWGHQRKLTEPMFQPAHLKYFYPSLRTITARLSARFARLAETGEVVSLVEEFKRYTVDITSLLAFGEDINTLEQGENPLSQSLRRMFPVINERCGSPIPLWRYIKRARDKQFDASLSLIDDHLNAFIDHQRERIRQNPQLLDAPENMLQIMLAEQQKDGTLTDADILANAFTLLLAGEDTTANTLTWMSFLLCSAPSMEENVVDECRQASGGEGGFLPWPLPRMPLLTAVMYESMRLKPVAPLLYLEPVKDTVIADFLIRKGTPLLLTLHANGFEETLFHHPHDFMPDRWLERGQASFSDLQPFGGGPRMCPGRSLALMEIKLGFHALCSGFRVEAQQPASDVMESFAFTVTPTGFYVRLHKRHQSDIARHEA

>CYP1247A2(A4U42_05050)Dickeya solani

MRSIKQLPMPPARGVLGHVHYLKRHDVHLQLLQWKERYGPFYRLRLGLASAMVIADSEWIRTIMKARPDEFRRRSSIESVFQEVGLNGVFSSEGARWMHQRKLTEPMFQPAHLKYFYPSLRKVTARLSERFSILAETGEVVSLVEEFKRYTVDITSLLAFGEDINTLEQGENPLSQSLRRMFPVINERCGSPIPLWRYIKRARDKQFDASLSLIYDHLNGFIDHQRERIRQNPLLLDTPENMLQIMLAEQQKDGTLTDADILANAFTLLLAGEDTTANTLAWMSFLLCSSPSMEEQVVDECSQVAEGSGRGLPWPLPRMPLLTAVMYESMRLKPVAPLLYLEPVKDTVIAGFLIKKGTPLLMMLHASGFEETLFHQPSDFMPERWLERGQASFSDLQPFGGGPRMCPGRSLALMEIKLGFHALCSGFRVEAQQPASDVIESFAFTVTPTGFRVRLHKQRQPDIAQHEA

>CYP1413A1(SerAS9_1502)Serratia plymuthica AS9

MTLCPYTRAVTAVTSDNPRADYLQMAPMQFQAQLQSWVAASPLAVREALNNRDLGVRPTGEPIPTLLLNTPAQSIFGALVRMQDGEVHPQLKAAIRQALAGIDEILIQQTTLTVAREIAPCLPDANQITRFNYALPVCVMASLLGVGADEWAELADEVLDFSRCIAPGGSESQRGKGILAAGRLSARFENRRGPLWLSLQRACAIRDMGHHTVLSNAIGLMFQACEGTAGLIGQTLLLMRNHDGDTQALIEKVLSDTPPIQNTRRFALRDTLVAGYRVLAGQDILILLCAGDESFAFGDGAHRCPGANWAKIIARYGIQHLSALGVDPQALNSFHWRVSQNARVPEFYL

>CYP1413A1(SerAS12_1502)Serratia sp. AS12

MTLCPYTRAVTAVTSDNPRADYLQMAPMQFQAQLQSWVAASPLAVREALNNRDLGVRPTGEPIPTLLLNTPAQSIFGALVRMQDGEVHPQLKAAIRQALAGIDEILIQQTTLTVAREIAPCLPDANQITRFNYALPVCVMASLLGVGADEWAELADEVLDFSRCIAPGGSESQRGKGILAAGRLSARFENRRGPLWLSLQRACAIRDMGHHTVLSNAIGLMFQACEGTAGLIGQTLLLMRNHDGDTQALIEKVLSDTPPIQNTRRFALRDTLVAGYRVLAGQDILILLCAGDESFAFGDGAHRCPGANWAKIIARYGIQHLSALGVDPQALNSFHWRVSQNARVPEFYL

>CYP1413A1(SerAS13_1503)Serratia sp. AS13

MTLCPYTRAVTAVTSDNPRADYLQMAPMQFQAQLQSWVAASPLAVREALNNRDLGVRPTGEPIPTLLLNTPAQSIFGALVRMQDGEVHPQLKAAIRQALAGIDEILIQQTTLTVAREIAPCLPDANQITRFNYALPVCVMASLLGVGADEWAELADEVLDFSRCIAPGGSESQRGKGILAAGRLSARFENRRGPLWLSLQRACAIRDMGHHTVLSNAIGLMFQACEGTAGLIGQTLLLMRNHDGDTQALIEKVLSDTPPIQNTRRFALRDTLVAGYRVLAGQDILILLCAGDESFAFGDGAHRCPGANWAKIIARYGIQHLSALGVDPQALNSFHWRVSQNARVPEFYL

>CYP1413B1(Z042_20910)Chania multitudinisentens

MMACPFSAATQQVVREALAHRDLGMRPPHEPVPAALLATPAQPLFAALVRMRDDAGHGELKAAISAALASFSDNELCQATHRVAQQLAPDMLTAEQLTRFNYALPIGVLADMLGVAYQERTVLVDNVLDFVRCIAPGGSEQQMARGVIAAGKLHEWMQAADGPLFIRLCQRIGDRSVAIANAIGLFFQACEGTAGLLGQTLLLMQKQDVTVEYGLSSVLQETPPIHTTRRFALRDTRLDGEPLVAGQTVLIALKTEGESFAFGYGSHQCPGSAWAHLIALGGIRHLLALNMESKLLTHFRWRVSQNAHVPEFFTAEEQ

>CYP1414A1(ECENHK_05600)Enterobacter cloacae subsp. cloacae ENHKU01

MAYVEKALRFNPASPVFQENLHNVYHHMRNHQPVARIGKTWVLTRYQDVYQTLKERAFVSSGITEDVHSEMEKECFSLSPPIRDLLYGIVLFEDGNVHRAHRQALQALFTGESWAALTQLISDESHTLVAELTTTGTFDGIRQIAAPLWGKLFTAWLNLPEAQQEVVEQEKSAIRLLLDPSAIDREGLQRLIVALSRLDDSFRQLAQAHSQGYDSLFYRSLLKGYGGDRDALATRFSTDCVTMLIGGSETSEALTGNLVYMLAQHPELQACVRNNTLRMKDVVSETMRFESPLQMGRRKVVAPVQFLGRELKAGDNILVCLGSANRDESVFEEAWRFIPGRKNAQRQLGFGAGVHQCIGQLLAQCQAETLAMALCERGTLSLDGEAKWSTRSLILRTLETLPVKIT

>CYP1414A2(A3UG_05565)Enterobacter cloacae subsp. dissolvens SDM

MAYVDKALRFNPASPVFQENLHNVYHHMRNHQPVARIGKTWVLTRYKDVYQALKERAFISSGIPEDVHRALEKERFSLSPPLLSLLYGIVLFEDGGVHRMHRQALQSLFMGESWEALTTIITRESQSLVAGLSTAYPFDGIRQIAAPLWGKLFSAWLNLPDELQAVVEEEKSAIRLLLDPSSIDRQGLERLLKALSHLDEGFSQLAQAHRKGYDSLFYRSLLNGYGGDDDALRERFSTDCVTMLIGGSETSEALTGNLLYMLAQHPELQERVQDNTVRMKDIVSETMRFESPLQMGRRKVAAPVEFLGRKLNVGDNVLLCLGSANRDETVFEEAGRFMPDRKNVQRQLGFGAGVHQCIGQLLAQCQAENLAMAVSERGIIALEDEAKWSNGSLILRTLESLPVKII

>CYP1414B1(AXX16_3285)Serratia rubidaea

MNSANDRTLRFNPASPSFNQYIYQIYQRMRQQQPLLRIGRTWVLTRYQDVSAALRAPQLSCSGIPRHLTAEFARLQGGLAPELAMLVQEMALFQDNGTHRQHRKPLMALFSREPLAQLRRLVVDEINRSIAALAKTDRLDVIAQLARPLWPRLFARWLNLSPQQSQVIEQEKESIRLLLDPSAIDRAGLERLASALRRLDTLFSQLYQECADGRPSLFFAALAQGYGDQQALMQRYFSADCVTILIGGSETTEALIGNLMQVVAQDEPLQQQLRQHPQWIAQAVQETLRYESPLQMARRTVIQPWTLHGRTLREQDAVLLCLGAANRDETQFCDAQQFDLHRENNSRHLGFGGSAHLCAGQLLARFQAESVCAALLQHFPRLRPLEQAQWQTDSLILRSLKSLPLALG

>CYP1415A1(Rahaq_4695)Rahnella sp. Y9602

MPDNAQAIPFFTPPSSQFARLGFLKIAEMACRQHGDKVWIGEKDNAVLLLAGARHVRLLIEQESQFVKEFEHLSSASTIGRILLGQSLTTSKEGEEWRLARKLTTPLVNPKSPLLKQSTDLSARWLLDILQDPQQTSLREICLHWALMCVAEGFFGREISLAQLNTLIGHFRDIYLQLIIAAPDADYEALCRHPALVAFRLEAESLLGPLLDDARGGNTTMLERLCQALSPGAHPEARERVISLLLGNLAASVDNTGIALLWTLTHLSQHLNYQHCVREEAAQGKRNMASAIVRESLRLTPVTAFFERRVAENIVVDDVVITAGTRVLFSPWLIHRHAACWAEPLCFRPERFLGEEKIAPEHFLPFSVGKRNCVGMTLALDQLTTAVATLCEHFQFSLAPSTSPAALTPLFALNVIPRGDLSFILASTNKAEQHDHIP

>CYP1415A1(Q7S_23996)Rahnella aquatilis HX2

MPDNAQAIPFFIPPSSQFARLGFLKIAEMACRQHGDKVWIGEKDNAVLLLAGARHVRLLIEQESQFVKEFEHLSSASTIGRILLGQSLTTSKEGEEWRLARKLTTPLVNPKSPLLKQSTDLSARWLLDILQDPQQTSLREICLHWALMCVAEGFFGREISLAQLNTLIGHFRDIYLQLIIAAPDADYEALCRHPALVAFRLEAESLLGPLLDDARGGNTTMLERLCQALSPGAHPEARERVINLLLGNLAASVDNTGIALLWTLTHLSQHLNYQHCVREEAVQGKRNMASAIVRESLRLTPVTAFFERRVAENIVVDDVVITAGTRVLFSPWLIHRHAACWAEPLCFRPERFLGEEKIAPEHFLPFSVGKRNCVGMTLALDQLTTAVATLCEHFQFSLAPSTSPAALTPLFALNVIPRGDLSFILTSTNKAEQHDHIP

>CYP51B1(MCA2711)Methylococcus_capsulatus

MSHPPSNTPPVKPGGLPLLGHILEFGKNPHAFLMALRHEFGDVAEFRMFHQRMVLLTGSQASEAFYRAPDEVLDQGPAYRIMTPIFGRGVVFDARIERKNQQLQMLMPALRDKPMRTYSEIIVAEVEAMLRDWKDAGTIDLLELTKELTIYTSSHCLLGAEFRHELNTEFAGIYRDLEMGIQPIAYVFPNLPLPVFKRRDQARVRLQELVTQIMERRARSQERSTNVFQMLIDASYDDGSKLTPHEITGMLIATIFAGHHTSSGTTAWVLIELLRRPEYLRRVRAEIDALFETHGRVTFESLRQMPQLENVIKEVLRLHPPLILLMRKVMKDFEVQGMRIEAGKFVCAAPSVTHRIPELFPNPELFDPDRYTPERAEDKDLYGWQAFGGGRHKCSGNAFAMFQIKAIVCVLLRNYEFELAAAPESYRDDYRKMVVEPASPCLIRYRRRDAPAAVDAKASAGEAPAETLRGAFRVTVDRDLCKGHGNCMAEAPEIFRVDEDGRLTLLSETPDPVLVGAALAAERFCPARAIKILPQRDPATRDRSLSPSGED

>CYP51B1(HDN1F_11100)Gamma_proteobacterium_HdN1

MTAAQPLPAPEPDLLAPPKLKGGVPLLGHIFPFARNPFQFMKRVSDELGEIAQFRIFNQRMVLLTGDAASTLFYRSSDDVLDQSAAYKLMTPIFGEGIVFDAPNERKNQQLKMLMPSLRAEAMRHHSDKIVQEVEDLAQRWGEQGEVDLVAEMKRLTINTASHCLLGREFRYELTDEFASIYHDLEKGVNALAYSFPNLPIPAFRARDRARVRLHQLVSEIVKKRESQAHKPNDMFQSLIDTHYEDGSKLTTDEITGILIAAIFAGHHTSSGTAAWVLLELLKHPHHLREVRNELDQLFGSDGVVSFQTLREIPHLENVLKEVLRLHPPLIVLMRQVVEPIRFKGYRIEAGDMVWASPPVTHRMSQLFPNPEVFDPTRYEGDHAADKNLMAWQPFGGGKHKCAGNAFALFQIKAIFSVLLRKYDFELVNRPDTYVDDYGEMIVQPKSPCMVRYKRRLPNSFASKFGTHASAEPVAAECPMHARSAKIPVTAVPDAKPASPENEVQGIRTFSVRVDRQLCQGHAICMGEAPELFRVDQNGYNHILQAHLDSEQLQKALNAAKYCPNQAISIHEDVRKENPQCENTE

>CYP101M1(PCA10_28250)Pseudomonas_resinovorans

MTAPLSELPDHVPADLVRHFDLYEPIEGDDDYQTWFTKLQAAGTPDIFWTRHNGGHWVVTRGEDFDHVLKTPETYSSRINVVPRERLMPIVSKPIQLDPPDHTRYRNLLIPAFSPKAVVPLGEKARALTIELIEGSLARGRCEFVSEFARRLPIGIFMDMVALPTADREQLLEWVDEMIRPTQVDSLEAGNQLIRYAFDQLQERRAEPGSDLLSELTRAQVGGNPLQDEELVGMFFLLLLGGLDTVAAMLSFIIRHLASHPEARRELIEHPERIPAAAEELMRRFPISTLVRVVTRDHEYKGINFREGDLVLMQTSAHSVDDRLFNDPLAVDFERKVVFHGTFGSGAHRCIGSMLARVEVRVFLEEWLKRIPEFRLKPGQPLRVEPGMVLAMPRLELEWDITESRP

>CYP107E27(B5T_02506)Alcanivorax_dieselolei

MEPPLSYPFNRFDGLELSERYAQALSRTGLTRITLPFDGDAWLATRYEDVRLVLSDRRFSRAEATQRERVPRAFPRVAGGIVIMDPPELTRLRKLALQAFTVRRVEGLRPHVREVADGLIDDMLRRGAPADLVADYALPIPMSVICELLGVPLADQPRFKIWNDSLLSTNALSAEETQQNLGELSHYIMALIDERRARPRDDLISAMIQARDQDDRLSQGELVLLCIAILVAGYEGVSSHIPNFLYTLLSRPDTLDRLRSDPDRMADAVEELLRLIPLASAAMFVHFAREDVQVGDTLVRAGEAVFASVGAANRDPHRFHQPDHLDIDRDASGHFAFGHGMHHCIGAGLARVELQEALGVLLRRLPEIHLCGEIEWKTKTFFRGPRRMPVAW

>CYP107E28 (VO64_5516)Pseudomonas_fluorescens_LBUM223

MTPLAYPFNAFTDLELAEPYRHAQQAPGLLRIQMPIGAPAWLATRYDDVRLVLGDRRFSRSEAFRRDDAPRAFPRIAGGIVMMDPPQLTRIRSRAAQAFTRRRVEALRPHARAYAHELIDRMLAAGPPADLVNDYALPLPLGLICELLGVPLQDRERFKLWNDSLLSTRPEDAALTQQHLGELAAYIKGLVAERRRQPQDDFMTALAQADDNGERLSEEQLLLLCIAILVAGYEGSASQIPNFIQVLLDNPAQWQQLKAHPEQIPQAVEELLRYIPLASAAMFVHYALEDIQVGETLVRQGEAVFASIGAANHDPARFADPQTLDLQRDASGHFGFGHGLHHCIGSALARVELQEALQALVERLPNLQRCGEVQWKTATFFRGAHCLPVTWS

>CYP107E29(PFLUOLIPICF724160)Pseudomonas_fluorescens_PICF7

MTPLDYPFNCFTDLKLAEPYRHAQQAPGLLRIQMPIGAPAWLATRYDDVRLVLGDRRFSRSEAFRRDDSPRAFPRIAGGIVMMDPPQLTRIRSRAAQAFTRRRVEALRPHARDYAHQLIDRMLAAGPPADLVNDYALPLPLALICELLGVPVQDRDRFKVWNDSLLSTRTEDAAQTQRHLGELAAYIKGLVAERRREPRDDFMTALTQTDDKGESLNEEQLLLLCIAILVAGYEGSAAQIPNFIQVLLDNPTQWQQLKADPEQIPQAVEELLRYIPLASAAMFVHYALEDIQVGETLVRQGDAVFASIGAANHDPARFENPQALDLHRDANGHFGFGHGLHHCIGSALARVELQEALHALVVRLPDLQRCGEVQWKTATFFRGAHCLPVTWS

>CYP107E30(PF1751_v1c25740)Pseudomonas_fluorescens_PCL1751

MTPLDYPFNCFTDLKLAEPYRHAQQAPGLLRIQMPIGAPAWLATRYDDVRLVLGDRRFSRSEAFRRDDSPRAFPRIAGGIVMMDPPQLTRIRSRAAQAFTRRRVEALRPHARDYAHQLIDRMLAAGPPADLVNDYALPLPLALICELLGVPVQDRDRFKVWNDSLLSTRTEDAAQTQRHLGELAAYIRGLVAERRREPRDDFMTALTQTDDKGESLNEEQLLLLCIAILVAGYEGSAAQIPNFIQVLLDNPTQWQQLKADPEQIPQAVEELLRYIPLASAAMFVHYALEDIQVGETLVRQGDAVFASIGAANHDPARFENPQALDLHRDANGHFGFGHGLHHCIGSALARVELQEALHALVVRLPDLQRCGEVQWKTATFFRGAHCLPVTWS

>CYP107S1(PA3331)Pseudomonas_aeruginosa_PAO1

MPDRKLRLGEELISPLHALYDGLQVDGAPRPAHRAAEHPVWVVTRYRDARKVLNHPGVRRDARQAAELYAKRTGSPRAGIGEGLSHHMLNLDPPDHTRLRSLVGRAFTPRQVERLQPHIERITEALLDAMAGREQADLMADFAIPLTIAVIFELLGIPEAEREHARQSWERQAELLSPEEAQALADAQVDYLRVLLEAKRRQPADDVYSGLVQAADESGQLSEAELVSMAHLLMMSGFETTMNMIGNALVTLLVNPEQLALLRAQPELLPNAMEELVRHDSPVRASMLRFTVEDVELDGVTIPAGEYILVSNLTANHDAERFDDPDRLDLTRNTDGHLGYGFGVHYCVGASLARLEGRIAIQRLLARFPDLQLAVPHAELQWLPITFLRALISVPVRTGCSAPANTASHANPIERIAQ

>CYP107S1(PLES_17331)Pseudomonas_aeruginosa_LESB58

MPDRKLRLGEELISPLHALYDGLQVDGAPRPAHRAAEHPVWVVTRYRDARKVLNHPGVRRDARQAAELYAKRTGSPRAGIGEGLSHHMLNLDPPDHTRLRSLVGRAFTPRQVERLQPHIERITEALLDAMAGREQADLMADFAIPLTIAVIFELLGIPEAEREHARQSWERQAELLSPEEAQALADAQVDYLRVLLEAKRRQPADDVYSGLVQAADESGQLSEAELVSMAHLLMMSGFETTMNMIGNALVTLLVNPEQLALLRAQPELLPNAMEELVRHDSPVRASMLRFTVEDVELDGVTIPAGEYILVSNLTANHDAERFDDPDRLDLTRNTDGHLGYGFGVHYCVGASLARLEGRIAIQRLLARFPDLQLAVPHAELQWLPITFLRALISVPVRTGCSAPANTASHANPIERIAQ

>CYP107S1(M062_17795)Pseudomonas_aeruginosa_RP73

MPDRKLRLGEELISPLHALYDGLQVDGAPRPAHRAAEHPVWVVTRYRDARKVLNHPGVRRDARQAAELYAKRTGSPRAGIGEGLSHHMLNLDPPDHTRLRSLVGRAFTPRQVERLQPHIERITEALLDAMAGREQADLMADFAIPLTIAVIFELLGIPEAEREHARQSWERQAELLSPEEAQALADAQVDYLRVLLEAKRRQPADDVYSGLVQAADESGQLSEAELVSMAHLLMMSGFETTMNMIGNALVTLLVNPEQLALLRAQPELLPNAMEELVRHDSPVRASMLRFTVEDVELDGVTIPAGEYILVSNLTANHDAERFDDPDRLDLTRNTDGHLGYGFGVHYCVGASLARLEGRIAIQRLLARFPDLQLAVPHAELQWLPITFLRALISVPVRTGCSAPANTASHANPIERIAQ

>CYP107S1(T223_08690)Pseudomonas_aeruginosa_LES431

MPDRKLRLGEELISPLHALYDGLQVDGAPRPAHRAAEHPVWVVTRYRDARKVLNHPGVRRDARQAAELYAKRTGSPRAGIGEGLSHHMLNLDPPDHTRLRSLVGRAFTPRQVERLQPHIERITEALLDAMAGREQADLMADFAIPLTIAVIFELLGIPEAEREHARQSWERQAELLSPEEAQALADAQVDYLRVLLEAKRRQPADDVYSGLVQAADESGQLSEAELVSMAHLLMMSGFETTMNMIGNALVTLLVNPEQLALLRAQPELLPNAMEELVRHDSPVRASMLRFTVEDVELDGVTIPAGEYILVSNLTANHDAERFDDPDRLDLTRNTDGHLGYGFGVHYCVGASLARLEGRIAIQRLLARFPDLQLAVPHAELQWLPITFLRALISVPVRTGCSAPANTASHANPIERIAQ

>CYP107S1(SCV20265_1717)Pseudomonas_aeruginosa_SCV20265

MPDRKLRLGEELISPLHALYDGLQVDGAPRPAHRAAEHPVWVVTRYRDARKVLNHPGVRRDARQAAELYAKRTGSPRAGIGEGLSHHMLNLDPPDHTRLRSLVGRAFTPRQVERLQPHIERITEALLDAMAGREQADLMADFAIPLTIAVIFELLGIPEAEREHARQSWERQAELLSPEEAQALADAQVDYLRVLLEAKRRQPADDVYSGLVQAADESGQLSEAELVSMAHLLMMSGFETTMNMIGNALVTLLVNPEQLALLRAQPELLPNAMEELVRHDSPVRASMLRFTVEDVELDGVTIPAGEYILVSNLTANHDAERFDDPDRLDLTRNTDGHLGYGFGVHYCVGASLARLEGRIAIQRLLARFPDLQLAVPHAELQWLPITFLRALISVPVRTGCSAPANTASHANPIERIAQ

>CYP107S1(AI22_25195)Pseudomonas_aeruginosa_YL84

MPDRKLRLGEELISPLHALYDGLQVDGAPRPAHRAAEHPVWVVTRYRDARKVLNHPGVRRDARQAAELYAKRTGSPRAGIGEGLSHHMLNLDPPDHTRLRSLVGRAFTPRQVERLQPHIERITEALLDAMAGREQADLMADFAIPLTIAVIFELLGIPEAEREHARQSWERQAELLSPEEAQALADAQVDYLRVLLEAKRRQPADDVYSGLVQAADESGQLSEAELVSMAHLLMMSGFETTMNMIGNALVTLLVNPEQLALLRAQPELLPNAMEELVRHDSPVRASMLRFTVEDVELDGVTIPAGEYILVSNLTANHDAERFDDPDRLDLTRNTDGHLGYGFGVHYCVGASLARLEGRIAIQRLLARFPDLQLAVPHAELQWLPITFLRALISVPVRTGCSAPANTASHANPIERIAQ

>CYP107S1(NCGM2_4461)Pseudomonas_aeruginosa_NCGM2.S1

MPDRKLRLGEELISPLHALYDGLQVDGAPRPAHRAAEHPVWVVTRYRDARKVLNHPGVRRDARQAAELYAKRTGSPRAGIGEGLSHHMLNLDPPDHTRLRSLVGRAFTPRQVERLQPHIERITEELLDAMAGREQADLMADFAIPLTIAVIFELLGIPEAEREHARQSWERQAELLSPEEAQALADAQVDYLRVLLEAKRRQPADDVYSGLVQAADESGQLSEAELVSMAHLLMMSGFETTMNMIGNALVTLLVNPEQLALLRAQPELLPNAMEELVRHDSPVRASMLRFTVEDVELDGVTIPAGEYILVSNLTANHDAERFDDPDRLDLTRNTDGHLGYGFGVHYCVGASLARLEGRIAIQRLLARFPDLQLAVPHAELQWLPITFLRALISVPVRTGCSAPANTASHANPIERIAQ

>CYP107S1(NCGM1900_2945)Pseudomonas_aeruginosa_NCGM_1900

MPDRKLRLGEELISPLHALYDGLQVDGAPRPAHRAAEHPVWVVTRYRDARKVLNHPGVRRDARQAAELYAKRTGSPRAGIGEGLSHHMLNLDPPDHTRLRSLVGRAFTPRQVERLQPHIERITEELLDAMAGREQADLMADFAIPLTIAVIFELLGIPEAEREHARQSWERQAELLSPEEAQALADAQVDYLRVLLEAKRRQPADDVYSGLVQAADESGQLSEAELVSMAHLLMMSGFETTMNMIGNALVTLLVNPEQLALLRAQPELLPNAMEELVRHDSPVRASMLRFTVEDVELDGVTIPAGEYILVSNLTANHDAERFDDPDRLDLTRNTDGHLGYGFGVHYCVGASLARLEGRIAIQRLLARFPDLQLAVPHAELQWLPITFLRALISVPVRTGCSAPANTASHANPIERIAQ

>CYP107S1(PADK2_07615)Pseudomonas_aeruginosa_DK2

MPDRKLRLGEELISPLHALYDGLQVDGAPRPAHRAAEHPVWVVTRYRDARKVLNHPGVRRDARQAAELYAKRTGSPRAGIGEGLSHHMLNLDPPDHTRLRSLVGRAFTPRQVERLQPHIERITEALLDAMAGREQADLMADFAIPLTIAVIFELLGIPEAEREHARQSWERQAELLSPEEAQALADAQVDYLRVLLEAKRRQPANDVYSGLVQAADESGQLSEAELVSMAHLLMMSGFETTMNMIGNALVTLLVNPEQLALLRAQPELLPNAMEELVRHDSPVRASMLRFTVEDVELDGVTIPAGEYILVSNLTANHDAERFDDPDRLDLTRNTDGHLGYGFGVHYCVGASLARLEGRIAIQRLLARFPDLQLAVPHAELQWLPITFLRALISVPVRTGCSAPANTASHANPIERIAQ

>CYP107S1(PA1R_gp1168)Pseudomonas_aeruginosa_PA1R

MPDRKLRLGEELISPLHALYDGLQVDGAPRPAHRAAEHPVWVVTRYRDARKVLNHPGVRRDARQAAELYAKRTGSPRAGIGEGLSHHMLNLDPPDHTRLRSLVGRAFTPRQVERLQPHIERITEALLDAMAGREQADLMADFAIPLTIAVIFELLGIPEAEREHARQSWERQAELLSPEEAQALADAQVDYLRVLLEAKRRQPADDVYSGLVQAADESGQLSEAELVSMAHLLMMSGFETTMNMIGNALVTLLVNPEQLALLRAQPELLPNAMEELVRHDSPVRASMLRFTVEDVELDGVTIPAGEYILVSNLTANHDAERFDDPDHLDLTRNTDGHLGYGFGVHYCVGASLARLEGRIAIQRLLARFPDLQLAVPHAELQWLPITFLRALISVPVRTGCSAPANTASHANPIERIAQ

>CYP107S1(U769_08190)Pseudomonas_aeruginosa_MTB-1

MPDRKLRLGEELISPLHALYDGLQVDGAPRPAHRAAEHPVWVVTRYRDARKVLNHPGVRRDARQAAELYAKRTGSPRAGIGEGLSHHMLNLDPPDHTRLRSLVGRAFTPRQVERLQPHIERITEALLDAMAGREQADLMADFAIPLTIAVIFELLGIPEAEREHARQSWERQAELLSPEEAQALADAQVDYLRVLLEAKRRQPADDVYSGLVQAADESGQLSEAELVSMAHLLMMSGFETTMNMIGNALVTLLVNPEQLALLRAQPELLPNAMEELVRHDSPVRASMLRFTVEDVELDGVTIPAGEYILVSNLTANHDAERFDDPDRLDLTRNTDGHLGYGFGVHYCVGASLARLEGRIAIQRLLARFPDLQLAVPHAELQWLPITFLRALISVPVRTGCSAPANTASHANPIERIVQ

>CYP107S1(BN889_03700)Pseudomonas_aeruginosa_PA38182

MLNLDPPDHTRLRSLVGRAFTPRQVERLQPHIERITEELLDAMAGREQADLMADFAIPLTIAVIFELLGIPEAEREHARQSWERQAELLSPEEAQALADAQVDYLRVLLEAKRRQPADDVYSGLVQAADESGQLSEAELVSMAHLLMMSGFETTMNMIGNALVTLLVNPQQLALLRAQPELLPNAMEELVRHDSPVRASMLRFTVEDVELDGVTIPAGEYILVSNLTANHDAERFDDPDRLDLTRNTDGHLGYGFGVHYCVGASLARLEGRIAIQRLLARFPDLQLAVPHAELQWLPITFLRALISVPVRTGCSAPANTASHANPIERIAQ

>CYP107S1(PA14_20970)Pseudomonas_aeruginosa_UCBPP-PA14

MPDRKLRLGEELISPLHALYDGLQVDGAPRPAHRAAEHPVWVVTRYRDARKVLNHPGVRRDARQAAELYAKRTGSPRAGIGEGLSHHMLNLDPPDHTRLRSLVGRAFTPRQVERLQPHIERITEELLDAMAGREQADLMADFAIPLTIAVIFELLGIPEAEREHARQSWERQAELLSPEEAQALADAQVDYLRVLLEAKRRQPADDVYSGLVQAADESGQLSEAELVSMAHLLMMSGFETTMNMIGNALVTLLVNPEQLALLRAQPELLPNAMEELVRHDSPVRASMLRFTVEDVELDGVTIPAGEYILVSNLTANHDAERFGDPDRLDLTRNTDGHLGYGFGVHYCVGASLARLEGRIAIQRLLARFPDLQLAVPHAELQWLPITFLRALISVPVRTGCSAPANTASHANPIERIAQ

>CYP107S1(PAM18_1634)Pseudomonas_aeruginosa_M18

MPDRKLRLGEELISPLHALYDGLQVDGAPRPAHRAAEHPVWVVTRYRDARKVLNHPGVRRDARQAAELYAKRTGSPRAGIGEGLSHHMLNLDPPDHTRLRSLVGRAFTPRQVERLQPHIERITEELLDAMADREQADLMADFAIPLTIAVIFELLGIPEAEREHARQSWERQAELLSPEEAQALADAQVDYLRVLLEAKRRQPADDVYSGLVQAADESGQLSEAELVSMAHLLMMSGFETTMNMIGNALVTLLVNPEQLALLRAQPELLPNAMEELVRHDSPVRASMLRFTVEDVELDGVTIPAGEYILVSNLTANHDAERFDDPDRLDLTRNTDGHLGYGFGVHYCVGASLARLEGRIAIQRLLARFPDLQLAVPHAELQWLPITFLRALISVPVRTGCSAPANTASHANPIERIAQ

>CYP107S1(G655_08105) Pseudomonas_aeruginosa_B136-33

MPDRKLRLGEELISPLHALYDGLQVDGAPRPAHRAAEHPVWVVTRYRDARKVLNHPGVRRDARQAAELYAKRTGSPRAGIGEGLSHHMLNLDPPDHTRLRSLVGRAFTPRQVERLQPHIERITEELLDAMAGREQADLMADFAIPLTIAVIFELLGIPEAEREHARQSWERQAELLSPEEAQALADAQVDYLRVLLEAKRRQPADDVYSGLVQAADESGQLSEAELVSMAHLLMMSGFETTMNMIGNALVTLLVNPEQLALLRAQPELLPNAMEELVRHDSPVRASMLRFTVEDVELDGVTIPAGEYILVSNLTANHDAERFDDPDRLDLTRNTDGHLGYGFGVHYCVGASLARLEGRIAIQRLLARFPDLQLAVPHAELQWLPITFLRALISVPVRTGCSAPANTASHANPIERIVQ

>CYP107DJ1(PSEBR_a5392)Pseudomonas_brassicacearum_subsp._brassicacearum_NFM421

MAYERTLDLNSDAFRGEAYRHYAALRQASPVFFSQAEGQLPMWYVTGAREVEEVLLDNERFARDPSRIDAQFAAMLGGEQSIAFLNDHMLNRDGDSHRRLRRLVNKAFTLKAVNALRPRIEQIAETLLEPVRSSGQMDVVGQYAFPLSITVIAELLGVPAQDRDDFRRWSHMIVQQVGHDLSELQRCYNEFAGYMLALIDRRRAQPGDDLVSALAQVEEEGSMLSQSELCSMIALLIIAGHETSASMIANAVHMLVQHPKALARLRDEPALMPGAVEEFLRYDSSVERAMVRFVTRDTELAGQRLLRGQLLIAVVGSANRDEALCANPDTLDITRPACPHMSFGKGTHYCLGASLARQELEIALNTLLRCCPGLQLAVDPGAVRWRYVPNFRGPEALPVCWSVG

>CYP107DJ1(PSF113_5612)Pseudomonas_fluorescens_F113

MAYERTLDLNSDAFRGEAYRHYAALRQASPVFFSQAEGQLPMWYVTGAREVEEVLLDNERFARDPSRIDAQFAAMLGGEQSIAFLNDHMLNRDGESHRRLRRLVNKAFTLKSVNALRPRIEQIAETLLEPVRSSGRMDVVSQYAFPLSITVIAELLGVPAQDRDDFRRWSHMIVQQVGHDLSELQRCYNEFAGYMLALIDQRRAQPGDDLVSALAQVEEEGSMLSQSELCSMIALLIIAGHETSASMIGNAVHMLVQHPKALARLRDEPALMPGAVEELLRYDSSVERAMVRFVTRDTELAGQRLQRGQLLIAVVGSANRDETLCANPDTLDITRPACPHMSFGKGTHYCLGASLARQELEIALNTLLRCCPGLQLAVDPGAVRWRYVPNFRGPEALPVRWSVD

>CYP107DJ2(NJ69_15465)Pseudomonas_parafulva

MANACTLDFNSDAFRSAAYQHYATLREAAPVFLSQPPGQLPIWYVTGAQDVETVLLDSERFARDPARISPQFAAMMGGDQSIAFLNDHMLNRDGERHRRLRRLVNKAFTLKAVNAMRPRIEQIAERLLDQAGPSGRLDVVSQYAFPLSIIVIAELLGVPAQDRDDFRRWSQLIVQQVGHDLSELQRCYAEFASYMLALIEQRRAAPTDDLVSALVEVEEDGNVLSSSELCSMIALLIVAGHETTASMIGNAVYLLLRHPHTLIQLRDSPDSMANAVEELLRYDSSVERAMVRFVTQDTELGGQRLQRGQLLMAVVGSANRDPALCAQPDILDVTRPACPHLAFGKGAHYCLGASLARQELEIALNTLLRRCPGLQLEGTPESVRWRYVPNFRGPQALPVRWSVA

>CYP107HT1(THITH_13855)Thioalkalivibrio_thiocyanoxidans

MIPIPATPATSQRVFDPRSPELVERPYDVYRHLRDEDPVHRSPFGMWVLSRYEDVYRALRDPRLSSKPSRYSVHAAHRNRSTPAAIAAQHMIMFLDAPEHTRLRGLLARVITDNLAANMRERIQKLVDDLLEGPLERGEMDIVRDLAIPLPLNVIAELLGIPAEDRSRLKEWSNWFFQIFSPVVSEEGRNRLNHAILEFREYLGALASERRRSPRQDIVSSLIAVSVDGDRLTDDEIFTSCLALFSNGQEALSHLVGNGMLALLQHPEQMRRLREDPGMIRNAVEELLRYDTPAQTVGRTATEAIELHGKVIPAGAPVYLLIGSANRDPCRFPNPDILDLSRPDCRHLSFGTGPHACLGAGLARVEAQAAILTLLTKTQDMELCGGPPTRLPNIFVRGLEALPVRFRAC

>CYP108R1(IMCC21906_02686) Spongiibacter_sp._IMCC21906

MNSETLKSVEPVVPEDIARIVVSGKSYAHDDVIYPAFKWLRGNMPLGKAYLDEYDPIWLVTKYDDVMEISRDADTYKNGIHNVLLQTRESDEFTRKMMNGKIRSLNSLAFMDAHEHKTYRDITAKWFMPNLIKRYEQRIREIAKESVDEFFALGGECDFVKDFALYYPLRVIMDLIGIPREDEAIMLKLTQELFAGEDPDERREGVDLGPDAVARAWHATMMEFYDYFRGLSAERQKNPKDDLISLIANHRVDGERIDEAHEFDYYIAVATAGHDTTSTAASGGVLGLIQYPEQFDLLKSDIGLVDKFVTESIRWTTPIKHFMRTASRDVELRGQLIHKNDRLMLCYPSANRDPDRFTDPDAFVIDRRPNQHLAFGNGLHMCLGQHLARLDMRILFQELIPRLNSIELTGTPKFIEATFTSGLKTMPVRYTGG

>CYP111A3(ACIAD1575)Acinetobacter_sp._ADP1

MNIKEQGMSRIDLKDPDLYQQRVPHDLFAQLRKEQPVYWNDEVDGSGFWAVMKHKDICEVSRNPAVFSSAYENGGHRIFNENEVGLTGAGESAVGIPFISIDPPLHTQYRKAIMPALSPARLGDIENRIRERAKLLIEQIPFDQEVDIVQLLSAPLPLLTLAELLGVKPDVWKDLYHWTNAFVGEDDPEFRQSPEAMQQTMIEFMSFCQALFEERRANPGHDIATLLATVPIDGKAPVLRDFIGNLILTLVGGNETTRNSMSHTIVNFCKNPEQWDKIKQNPELLKTATAEMVRHASPVLHMRRTATQDTMIGEQKIAKGDKVVLWYSSANRDEDVFERPDEFDVTRKGFQHVGFGFGQHVCVGSRLAEMQLRVVFELLAERVERFELKSEPRRFRSNFLNGLKNLNVVLVAK

>CYP112A5(XOC_0084) Xanthomonas_oryzae_pv._oryzicola_BLS256

MSDDPLPMLPMWRVDHIEPSPAMLALRANGPIHRVRFPSGHEGWWVTGYDEAKAVLSDAAFRPAGMPPTEFTPDSVILGSPGWLVSHEGIEHARLRTIVAPAFSNRRVKLLAEQVETIAAQLFETLAAQAQPADLRRHLSFPLPAMVISALMGVPYEDHTFFAGLSDEVMTHQHESGPRRASRVAWEELRAYIRGKMRGKREEQGDDLLTDLLAAVDQGKASEEEAIGLAAGMLVAGHESTVAQIEFGLLALLRHPQQRERLVTDPSLAESAVEEILRMYPPGAGWDGIMRYPRVDVDIAGVHIPAESKVLVGLPATAFDPRHFDDPEIFDIGRDAKPHLAFSYGPHYCIGVALARLELKVVFGSIFQRFPALRLAVAPEALTLRKEIITGGFEAFPVRW

>CYP112A5(BE73_23905)Xanthomonas_oryzae_pv._oryzicola_CFBP7342

MSDDPLPMLPMWRVDHIEPSPAMLALRANGPIHRVRFPSGHEGWWVTGYDEAKAVLSDAAFRPAGMPPTEFTPDSVILGSPGWLVSHEGIEHARLRTIVAPAFSNRRVKLLAEQVETIAAQLFETLAAQAQPADLRRHLSFPLPAMVISALMGVPYEDHAFFAGLSDEVMTHQHESGPRRASRVAWEELRAYIRGKMRGKREEQGDDLLTDLLAAVDQGKASEEEAIGLAAGMLVAGHESTVAQIEFGLLALLRHPQQRERLVTDPSLAESTVEEILRMYPPGAGWDGIMRYPRVDVDIAGVHIPAESKVLVGLPATAFDPRHFDDPEVFDIGRDAKPHLAFSYGPHYCIGVALARLELKVVFGSIFQRFPALRLAVAPEALTLRKEIITGGFEAFPVRW

>CYP112A6(FD63_13540)Xanthomonas_translucens

MSQSELVALPMWRVEHIEPSPAMLMLCADGPIHRVRFPSGHEGWWVTGYDEAKAVLSDERFRPAGMPPAEFTPDSVILGSPGWLVSHEGIEHARLRTIVAPAFSNRRVKLLAQQVEAIATQLFDALEAQPQPADLRHALSFPLPAMVISALMGVPYEDHAFFAGLSDEVMTHQHESGPRNASRLAWEELRAYIRGKMREKRQAPGGNLLTDLMQAVDRGEATEEEAIGLAAGMLVAGHESTVAQIEFGLLALFRYPQQRERLVGDPSLVEKAVEEILRMYPPGAGWDGIMRYPRTDVTIAGVHIPAESKVLVGLPATAFDPRHFNDPEVFDIGRDEKPHLAFSYGPHYCIGVALARLELKVVFGSIFQRFPGLRLAVAPDELRLRKEIITGGFEEFPVRW

>CYP114A(XOC_0082)Xanthomonas_translucens

MAEMRVVVDQALCATTGQCALTLPAVFRQRVSDGVAEVCVAEVPPALHAAARLAASQCPVAAIRIIDADADAAGTGGGPASSQAEPSIASAPRNSGGHDGTM

>CYP114A5(FD63_13535)Xanthomonas_oryzae_pv._oryzicola_BLS256

MDVQNTTAVCRDAFAELASPACIRDPYPFMHWLREHDPVHRAASGIFLLSRHADIYWALKATGDAFRGPAPGELARYFPRAATSLSLNLLASTLAMKEPPTHTRLRRLVSRDFTVRQIDSLRPSIVRIVEARLDGMAPALARGETVDLHREFALALPMLVFAELFGMPQEDMSGLAAGIGTILEGLSPHASDAQLAAADATSAKVQAYFGALIERKRTESRQDIVSLLVGAHDDDADALSDAELISMLWGMLLGGFATTAATIDHAVLAMLAYPEQRHWLQGDAAAVKAFVEEVLRCEAPAMFSSIPRIAQRDIALDGGVIPKNADVRVLIAAGNRDPNAFADPDRFDPARFHHTSPGMSTEGNIMLSFGHGIHFCLGAQLARVQLAESLPRIHARFPTLVLAEEPTREPSAFLRTFRVLPVRLHSVGA

>CYP114A6(XOC_0083) Xanthomonas_oryzae_pv._oryzicola_CFBP7342

MAVQAAHSATCRDAFAELASPACIDDPYPFMRWLREHDPVHRAASGLFLLSRHADIYWALKATGDAFGGPAPGELARHFPRAATSLSLQLLASTLAMKEPPTHTRLRRLISRDFTMRQIEALRPSIARIVAARLDAMAPALQRGETVDLHREFALAVPMLVFAELFGVPQDDMFALATGVGTVLGGLSPHASDSQLATADTASAAVMRYFGDLIERKRAAPGQDMVSMLVGAHDDDADMLSDTELISMLWGMLLGGFATTAATFDHAVLAMLAYPEQLHWLQGDAAGVKAFVEEVLRCDAPAMFSSIPRIAQRDIELDGVVIPKDSDVRVLIAAGNRDPEAFADPDRFDPARFHGTSPGMSTDGKVMLSFGHGIHFCLGAQLARVELAESLPQIHARFPALALAAPPTREPSAYLRTFRALPVRLAVHDG

>CYP114A6(BE73_23910)Xanthomonas_oryzae_pv._oryzicola_BLS256

MAVQAAHSASCRDAFAELASPACIDDPYPFMRWLREHDPVHRAASGLFLLSRHADIYWALKATGDAFGGPAPGELARHFPRAATSLSLQLLASTLAMKEPPTHTRLRRLISRDFTMRQIEALRPSIARIVAARLDAMAPALQRGETVDLHREFALAVPMLVFAELFGVPQDDMFALATGVGTVLGGLSPHASDSQLATADTASAAVMRYFGDLIERKRAAPGQDMVSMLVGAHDDDADMLSDTELISMLWGMLLGGFATTAATFDHAVLAMLAYPEQLHWLQGDAAGVKAFVEEVLRCDAPAMFSSIPRIAQRDIELDGVVIPKDSDVRVLIAAGNRDPEAFADPDRFDPARFHGTSPGMSTDGKVMLSFGHGIHFCLGAQLARVELAESLPQIHARFPALALAAPPTREPSAYLRTFRALPVRLAVHDG

>CYP115A4(BE73_23900)Xanthomonas_oryzae_pv._oryzicola_CFBP7342

MARVALPGNVVTWAAGHHSTLGRLLADQRFNRDWRHWRALQDGEIPENHPLIGMCKLDNMVTAHGADHRRLRGLLARSFAPSRIALLAPRIERCVDALLTEMLRRGSAELMGEFAIPLPTSVIADLFGLPDDQREEIVLLTNSLANTSASEEEVRQTRQRIPEFFHRLIARKRREPGDDLASALIAGRDNGQLVSDTELIDMLFMVLSAGFVTTSGVIGNGVLALLTQPQQLHLVLSGQVPWSQAIEEILRWGSSVTNLPFRYATQGMQIAGVSLRRGDAVLMAFHAANRDEEAFGPGAAALDVTRRHNPHLSFGQGPHFCLGAALARLELRCAFPALFTRLEDLALAIAAEDVAYMPSYVIRCPQRLPITFRPSAV

>CYP115A4(XOC_0085) Xanthomonas_oryzae_pv._oryzicola_BLS256

MSLAILQRPGMARVALPGNVVTWAAGHHSTLGRLLADQRFNRDWRHWRALQDGEIPENHPLIGMCKLDNMVTAHGADHRRLRGLLARSFAPSRIALLAPRIERCVDALLTEMLRRGSAELMGEFAIPLPTSVIADLFGLPDDQREEIVLLTNSLANTSASEEEVRQTRQRIPEFFHRLIARKRREPGDDLASALIAGRDNGQLVSDTELIDMLFMVLSAGFVTTSGVIGNGVLALLTQPQQLHLVLSGQVPWSQAIEEILRWGSSVTNLPFRYATQGMQIAGVSLRRGDAVLMAFHAANRDEEAFGPGAAALDVTRRHNPHLSFGQGPHFCLGAALARLELRCAFPALFTRLEDLALAIAAEDVAYMPSYVIRCPQRLPITFRPSAV

>CYP115A5(FD63_13545)Xanthomonas_translucens

MRVEDDRGDVINISVAQSQLDNASSSIRQRNGMARVALPGNVVTWAAGRHQTLKCILADQRFSRDWRHWRALQDGEIPEDHPLIGMCRLDNMVTAHGADHQRLRGLLARSFAPSRIALLAPDIERRVDQLLAGIAARGSADLMQEFAVPLPASVIAELFGLPEENRAEIILLTNSLASTSASVAEVQQTRQRIPDFFRWLIALKRRRPGDDLASALIAARDSGELVSDTELIDMLFMVLSAGFVTTTGVIGNGVLALLTHPQQLRLVLSGQVTWAQAIEEILRWGSSVTNLPFRYATQDVEIEGCTIRRGEAILMAFHAANRDERAFGPGAEAFDVTRQPNPHLSFGQGPHFCLGAALARLELLYAFPALFARLHELALAVALEDIAYQPSYVIRCPQRLPVTFRPSIARK

>CYP116B45(FF32_18265)Halomonas_campaniensis

MTTPARQPDKMSEGCPFHQTAYATSHATSQATVTSPTGCPVSRDAAAFDPFGASYQLDPAEALRWSREQEPVFFSPKLGYWVVSRYEEVKAVFRDNLTFSPAIALEKLTPAPPEAVKILESYGFAMRRTMVNEDEPDHMERRRLLMDAFLPENLEKHEVWVRELARSYMDHFIDKGRADLVEEMFREIPMTVALRFLGVPSEDAKELRKFSVAHTLNTWGRPSPEQQLKIAEDVGQFWQTAQAILDRMREEPTGEGWMYDSIRMHNQHPEIVPESYLRSMMMAILVAAHETTAFATTNAFRILLSNRASWNDICENPTLIPSAIEECLRAAGSVVAWRRIATEDADVGGVTIPKGGKLLLVQASANRDSRHFENPDEFDIYRHNSAEHFTFGYGAHQCMGKNIARMEMRIILDEFVRRLPHIHLIEDQTFEYLPNTSFRGPTSLWVEWNPEQNPERHNRAVLENPTHFHIGAPIKEDIVRRVVVAEVEREAEEVVRIDLVDPYGRELPDWSPGSHIELVSGKWRRYYSLCGKRNDQHRLSIAILREPTGRGGSLYFHEMIKPGDVLHIAGPKNHFHLDETAERYTLIAGGIGITPILTMADQLKALGKPYTLHYCGAGRQTMAFLERVERDHSEALTVHASDEGCRLDISTALSNVAKGDQVYCCGPERMLEALEVLAQNWPEGILHVEHFSARSNILDPEQEHTFEVVLSDSYVTLQVGNDQTLLEALTAFGVDVPSDCCEGLCGTCEVAVVEGDIDHRDVVLSRAERAASDRMMACCSRAKGDRLVLAL

>CYP116B176(LOKO_00991)Halomonas_chromatireducens

MTTNSRRAGAEAGGCPIHQPGRQQDLAPNGCPISPRAAAFDPFDRPYQLDPAEALRWSREQEPVFYSPRLGYWVVSRYDDIKAIFRDNITFSPSIALEKITPASKEAQAVLERYDYGMNRTLVNEDEPAHMARRRELLEAFSPEALEAHAPMVRRLVREKLDAIVDRGRADLVDEMFWEVPLTVALHFLGVPEEDMEQLRRFSVAHTLNTWGRPSPEQQVEVAEGVGKFWQYSGEVLVKMQRQTRGKGWMYDMIEKNRQKPDVVTDNYLHSMMMAIIVAAHETTALATANAFRQLLSRPAVWDELCDNPELIPAAAEECLRHSGSVVAWRRRATREVAVGGVTIPEDGKILMVTASGNHDPSHFENPDELDIYRDNAVDHLTFGYGSHQCMGKNLGRMEMRIFLEEFTRRLPHLELEEQEFTFLPNTSFRGPEALWVRWDPAKNPERQDPAVRTAQRDFAVGAPSRQDIARTMVVAKVQAAADGVLQIALEDPRGRRVPAWSPGSHVDLIMGDYVRKYSLCGETDDPYWLQVAVLREEAGRGGSAWIHEHFEPGMTLRLRGPKNHFRLDESAQHYVLIAGGIGITPIIAMADRLRRLGKSYELHYAGRSRSSMAFIERLERDHGEALQLYPKDEGRRLDLAGLLAEPREATLLYACGPERLLTALEGGTAHWPEGSLHVEHFTAEGALLDPENEHAFEVELTDSELTVEVPPERTLLQVLRTAGIDVPSDCEEGLCGSCQVEVVEGEVDHRDKVLTAAERASQDRLMSCCSRARGRKLVLAL

>CYP117A5(FD63_13520)Xanthomonas_translucens

MAMLLNPLDRRPRLRHDIPVMRGAFPLVGHLPAIVCDLPNLLQRAEQTLGNHFWLDFGPAGQLMTCLDPEAFALLRHKDVSSALIEEIAPELLGGTLVAQDGAVHRQARDAIKAAFLPKGLTQAGIGELFAPVIQTRLQTWRDRGEVAILRETGDLMLKLIFSLMGIPAQDLPGWRRKYHQLLQLIVAPPVDLPGLPLRRGRAAREWIDAQLRQFIRDARAHAARNGLLNDMVNAFDHGDDALSDDVLVANIRLLLLAGHDTTASTMAWMVIELARQPALWDALVEEARRVGEVPTQPADLAQCRVAEALFRETLRVHPATTLLPRRTLQELQLGQRRVPAGTRLCIPLLYFSTSALLHEEADLFRLERWLQRTEPIRPVDMLQFGTGPHVCIGYHLVWLELVQFCIALALTMDKAGVRPRLLGDVEKGRRYYPTAHPSMGIHIGFS

>CYP117A6(BE73_23925)Xanthomonas_oryzae_pv._oryzicola_CFBP7342

MDVLRNPLNRRQRLRQDIPVVPGAYPLVGHLPAIVCDLPRLLQQAEHTLGRHFWLDFGPAGQFMTCVDPDAFALLRHKDVSSALIEEIAPELLGGTLVAQDGSAHRQARDAIKAAFLPKGLTRAGIGELFAPLIQARVEAWRERGDVFILRETGELMLTLIFSLMGIPAHDLPGWQRKYRQLLQLIVAPPLNLPGLPLRRGRAARDWIDAQLRQFVRDARSHAARTGLINDMVSAFDRSNDALADDVLVANIRLLLLAGHDTTASTMAWMVIELARQPMLWEALVDESQRMGAVPTQPADLAACPVAEALFRETLRMHPATTLLPRRAVQDLQLGQQRIPAGTHLCIPLLHFSSSPLLHEAPEQFRLARWLQRSEPIRPLDMLQFGTGAHVCIGYHLVWLELVQFCIALALTMHKAGLRPRVLNDVDKGRRYYPTAHPSMAVHIGFA

>CYP117A6(XOC_0080) Xanthomonas_oryzae_pv._oryzicola_BLS256

MDVLRNPLNRRQRLRQDIPVVPGAYPLVGHLPAIVCDLPRLLQQAEHTLGRHFWLDFGPAGQFMTCVDPDAFALLRHKDVSSALIEEIAPELLGGTLVAQDGSAHRQARDAIKAAFLPKGLTRAGIGELFAPLIQARVEAWRERGDVFILRETGELMLTLIFSLMGIPAHDLPGWQRKYRQLLQLIVAPPLNLPGLPLRRGRAARDWIDAQLRQFVRDARSHAARTGLINDMVSAFDRSNDALADDVLVANIRLLLLAGHDTTASTMAWMVIELARQPMLWEALVDESQRMGAVPTQPADLAACPVAEALFRETLRMHPATTLLPRRAVQDLQLGQQRIPAGTHLCIPLLHFSSSPLLHEAPEQFRLARWLQRSEPIRPLDMLQFGTGAHVCIGYHLVWLELVQFCIALALTMHKTGLRPRVLNDVDKGRRYYPTAHPSMAVHIGFA

>CYP133B1(XF_0377)Xylella_fastidiosa_9a5c

MKLTDLSNPAFLENPYPLYETLRAQAPFVSIGPNALMTGRYSLVDSLLHNRNMGKKYMESMRVRYGDSAADMPLFQAFSRMFITINPPAHTHLRGLVMQAFTGRESESMRPLAIDTAHQLIDNFEQKPSVDLVAEFAFPFPMQIICKMMDVDIGDAVTLGIAVSKIAKVFDPSPMSADELVHASTAYEELAQYFTKLIELRRTHPGTDLISMFLRAEEDGEKLTHDEIVSNVIMLLIAGYETTSNMIGNALIALHRHPEQLALLKSDLSLMPQAVSECLRYDGSVQFTMRAAMDDIEVEGELVPRGTVVFLMLGAANRDPAQFTHPDQLDITRKQGRLQSFGAGIHHCLGYRLALIELECALTTLFERLPHLRLAHLDALNWNQRSNLRGVNTLIVDLHAKN

>CYP133B1(PD_1688)Xylella_fastidiosa_Temecula1

MKLTDLSNPAFLENPYPLYETLRAQAPFVSIGPNALMTGRYSLVDSLLHNRNMGKKYIESIRLRYGDTAADMPLFQAFSRMFITINPPAHTHLRGLVMQAFTGRESESMRPLAIDTAHQLIDNFEQKPSVDLVAEFAFPFPMQIICKMMDVDIGDAVTLGMAVSKIAKVLDPSPMSADELVHASTAYEELAQYFTKLIELRRTHPGTDLISMFLRAEEDGEKLTHDEIVSNVIMLLIAGYETTSNMIGNALIALHRHPEQLALLKSDLSLMPQAVSECLRYDGSVQFTMRAAMDDIEVEGELVPRGTVVFLMLGAANRDPAQFTHPDQLDITRKQGRLQSFGAGIHHCLGYRLALIELECALTTLFERLPHLRLAHLDALNWNQRSNLRGVNTLIVDLHAKN

>CYP133B1(XfasM23_1782)Xylella_fastidiosa_M23

MKLTDLSNPAFLENPYPLYETLRAQAPFVSIGPNALMTGRYSLVDSLLHNRNMGKKYIESIRLRYGDTAADMPLFQAFSRMFITINPPAHTHLRGLVMQAFTGRESESMRPLAIDTAHQLIDNFEQKPSVDLVAEFAFPFPMQIICKMMDVDIGDAVTLGMAVSKIAKVLDPSPMSADELVHASTAYEELAQYFTKLIELRRTHPGTDLISMFLRAEEDGEKLTHDEIVSNVIMLLIAGYETTSNMIGNALIALHRHPEQLALLKSDLSLMPQAVSECLRYDGSVQFTMRAAMDDIEVEGELVPRGTVVFLMLGAANRDPAQFTHPDQLDITRKQGRLQSFGAGIHHCLGYRLALIELECALTTLFERLPHLRLAHLDALNWNQRSNLRGVNTLIVDLHAKN

>CYP133B1(XFLM_02730)Xylella_fastidiosa_subsp._fastidiosa_GB514

MKLTDLSNPAFLENPYPLYETLRAQAPFVSIGPNALMTGRYSLVDSLLHNRNMGKKYIESIRLRYGDTAADMPLFQAFSRMFITINPPAHTHLRGLVMQAFTGRESESMRPLAIDTAHQLIDNFEQKPSVDLVAEFAFPFPMQIICKMMDVDIGDAVTLGMAVSKIAKVLDPSPMSADELVHASTAYEELAQYFTKLIELRRTHPGTDLISMFLRAEEDGEKLTHDEIVSNVIMLLIAGYETTSNMIGNALIALHRHPEQLALLKSDLSLMPQAVSECLRYDGSVQFTMRAAMDDIEVEGELVPRGTVVFLMLGAANRDPAQFTHPDQLDITRKQGRLQSFGAGIHHCLGYRLALIELECALTTLFERLPHLRLAHLDALNWNQRSNLRGVNTLIVDLHAKN

>CYP133B1(Xfasm12_1853)Xylella_fastidiosa_M12

MKLTDLSNPAILENPYPLYETLRAQAPFVSIGPNALMTGRYSLVDSLLHNRNMGKNYMESMRVRYGDSAADMPLFQAFNRMFITINPPAHTHLRGLVMQAFTGRESESMRPLVIDTAHQLIDNFEQKPSVDLVAEFAFPFPMQIICKMMDVDIGDAVTLGMAVSKIAKVFDPSPMSADELVHASTAYEELAQYFTKLIELRRTHPGTDLISMFLRAEEDGEKLTHDEIVSNVIMLLIAGYETTSNMIGNALIALHRHPEQLTLLKSDLSLMPQAVSECLRYDGSVQFTMRAAMDDIEVEGELVPRGTVVFLMLGAANRDPAQFTHPDQLDITRKQGRLQSFGAGIHHCLGYRLALIELECALTALFERLPHLRLAHLDALNWNQRSNLRGVNTLIVDLHAKN

>CYP133B1(P303_08560) Xylella_fastidiosa_MUL0034

MKLTDLSNPAFLENPYPLYETLRAQAPFVSIGPNALMTGRYSLVDSLLHNRNMGKKYMESIRLRYGDSAADMPLFQAFSRMFITINPPAHTHLRGLVMQAFTGRESESMRPLAIDTAHQLIDNFEQKPSVDLVAEFAFPFPMQIICKMMDVDIGDAVTLGMAVSKIAKVLDPSPMSADELVHASTAYEELAQYFTKLIELRRTHPGTDLISMFLRAEEDGEKLTHDEIVSNVIMLLIAGYETTSNMIGNALIALHRHPEQLALLKSDLSLMPQAVSECLRYDGSVQFTMRAAMDDIEVEGELVPRGTVVFLMLGAANRDPAQFTHPDQLDITRKQGRLQSFGAGIHHCLGYRLALIELECALTTLFERLPHLRLAHLDALNWNQRSNLRGVNTLIVDLHAKN

>CYP133B2(D934_08745)Xylella_fastidiosa_subsp._sandyi_Ann-1

MKLADLSSPAFLENPYPLYETLRRQGPFVSIGPNALMTGRYSIVDGLLHNRNMGKSYMESIRVRYGDDALDMPLFQGFNRMFLMLNPPVHTHLRGLVMQAFTGRESESMRPLATDTAHRLIDDFEQKSSVDLVTEFSFPLPMRIICRMMDVDISDAISLSVAVSNIAKVFDPAPMSPDELVHASAAYEELAHYFTRLIELRRAQPGTDLISMLLRAEEEGQKLTHDEIVSNVILLLLSGYETASNMIGNALIALHRHPKQLARLKSDLSLMPQTVLECLRYDGSVQFTVRAAMDDVSIEGDVVPRGTIVFLMLGAANRDPAQFTDPDHLEITRKQGRLQSFGAGVHHCLGYRLALVELECALTVLLERLPHLRLANLDTLSWNQRGNLRGVNALIADLHP

>CYP133B2(XfasM23_1797)Xylella_fastidiosa_M23

MKLADLSSPAFLENPYPLYETLRRQGPFVSIGPNALMTGRYSIVDGLLHNRNMGKSYMESIRVRYGDDALDMPLFQGFNRMFLMLNPPVHTHLRGLVMQAFTGRESESMRPLAIDTAHRLIDDFEQKSSVDLVTEFSFPLPMRIICRMMDVDISDAISLSVAVSNLAKVFDPAPMSPDELVHASAAYEELAHYFTRLIELRRAQHGTDLISMLLRAEEEGQKLTHDEIVSNVILLLLGGYETTSNMIGNALIALHRHPKQLARLKSDLSLMPQAVLECLRYDGSVQFTIRAAMDDVSIEGDVVPRGTIVFLMLGAANRDPAQFTDPDHLEITRKQGRLQSFGAGVHHCLGYRLALVELECALTVLLERLPHLRLANLDTLSWNQRGNLRGVNALIADLH

>CYP133B2(XFLM_02805)Xylella_fastidiosa_subsp._fastidiosa_GB514

MKLADLSSPAFLENPYPLYETLRRQGPFVSIGPNALMTGRYSIVDGLLHNRNMGKSYMESIRVRYGDDALDMPLFQGFNRMFLMLNPPVHTHLRGLVMQAFTGRESESMRPLAIDTAHRLIDDFEQKSSVDLVTEFSFPLPMRIICRMMDVDISDAISLSVAVSNLAKVFDPAPMSPDELVHASAAYEELAHYFTRLIELRRAQHGTDLISMLLRAEEEGQKLTHDEIVSNVILLLLGGYETTSNMIGNALIALHRHPKQLARLKSDLSLMPQAVLECLRYDGSVQFTIRAAMDDVSIEGDVVPRGTIVFLMLGAANRDPAQFTDPDHLEITRKQGRLQSFGAGVHHCLGYRLALVELECALTVLLERLPHLRLANLDTLSWNQRGNLRGVNALIADLH

>CYP133B2(P303_08635)Xylella_fastidiosa_MUL0034

MKLADLSNPAFLENPYPLYETLRRQGPFVSIGPNALMTGRYSIVDGLLHNRNMGKSYMESIRVRYGDDALDMPLFQGFNRMFLMLNPPVHTHLRGLVMQAFTGRESESMRPLAIDTAHRLIDDFEQKSSVDLVTEFSFPLPMRIICRMMDVDISDAISLSVAVSNLAKVFDPAPMSPDELVHASAAYEELAHYFTRLIELRRAQHGTDLISMLLRAEEEGQKLTHDEIVSNVILLLLGGYETTSNMIGNALIALHRHPKQLARLKSDLSLMPQTVLECLRYDGSVQFTIRAAMDDVSIEGDVVPRGTIVFLMLGAANRDPAQFTDPEHLEITRKQGRLQSFGAGVHHCLGYRLALVELECALTVLLERLPHLRLANLDTLSWNQRGNLRGVNALIADLHP

>CYP133B3(XCAW_0345)Xanthomonas_citri_subsp._citri_Aw12879

MSQAMLLSDLATPQFRHDPYPTYARLREEGPLVQVADGRLMSGRYAVVDRLLSDRRVGRDYLQSVRLRYGEAAVHLPLFQGMSRMFLLLNPPLHTQLRGLMTQAFGARQMESMREVASDIAAGLIDAFQANGHCDLLTEFAFPLPIAIICRMLDIAAADVTALSHATSALAKVFDPMMTAEELQATSVAYDQLATYFHGVIAQRRSAGGDDLIARFIQAEDNGRRLSEEEIVSNVILLFFAGHETTSNMICNALVALHRHPQQLRLLQETPGLLPNAVLECMRYDSSVQMATRTALQDFEIEGVAVPRGTMLYLMLGAANHDTLQFTDPQVLDIRRQQGRALSLGGGIHHCLGNRLALIEVEAALACLLARLPALRLEQLDTLSWNDRANLRGVDALLASW

>CYP133B3(J151_03352)Xanthomonas_citri_subsp._citri_A306

MLLSDLATPQFRHDPYPTYARLREEGPLVQVADGRLMSGRYAVVDRLLSDRRVGRDYLQSVRLRYGEAAVHLPLFQGMSRMFLLLNPPLHTQLRGLMTQAFGARQMESMREVASDIAAGLIDAFQANGHCDLLTEFAFPLPIAIICRMLDIAAADVTALSHATSALAKVFDPMMTAEELQATSVAYDQLATYFHGVIAQRRSAGGDDLIARFIQAEDNGRRLSEEEIVSNVILLFFAGHETTSNMICNALVALHRHPQQLRLLQETPGLLPNAVLECMRYDSSVQMATRTALQDFEIEGVAVPRGTMLYLMLGAANHDTLQFTDPQVLDIRRQQGRALSLGGGIHHCLGNRLALIEVEAALACLLARLPALRLEQLDTLSWNDRANLRGVDALLASW

>CYP133B3(J159_03327)Xanthomonas_citri_subsp._citri_UI7

MLLSDLATPQFRHDPYPTYARLREEGPLVQVADGRLMSGRYAVVDRLLSDRRVGRDYLQSVRLRYGEAAVHLPLFQGMSRMFLLLNPPLHTQLRGLMTQAFGARQMESMREVASDIAAGLIDAFQANGHCDLLTEFAFPLPIAIICRMLDIAAADVTALSHATSALAKVFDPMMTAEELQATSVAYDQLATYFHGVIAQRRSAGGDDLIARFIQAEDNGRRLSEEEIVSNVILLFFAGHETTSNMICNALVALHRHPQQLRLLQETPGLLPNAVLECMRYDSSVQMATRTALQDFEIEGVAVPRGTMLYLMLGAANHDTLQFTDPQVLDIRRQQGRALSLGGGIHHCLGNRLALIEVEAALACLLARLPALRLEQLDTLSWNDRANLRGVDALLASW

>CYP133B3(J169_03350)Xanthomonas_citri_subsp._citri_NT17

MLLSDLATPQFRHDPYPTYARLREEGPLVQVADGRLMSGRYAVVDRLLSDRRVGRDYLQSVRLRYGEAAVHLPLFQGMSRMFLLLNPPLHTQLRGLMTQAFGARQMESMREVASDIAAGLIDAFQANGHCDLLTEFAFPLPIAIICRMLDIAAADVTALSHATSALAKVFDPMMTAEELQATSVAYDQLATYFHGVIAQRRSAGGDDLIARFIQAEDNGRRLSEEEIVSNVILLFFAGHETTSNMICNALVALHRHPQQLRLLQETPGLLPNAVLECMRYDSSVQMATRTALQDFEIEGVAVPRGTMLYLMLGAANHDTLQFTDPQVLDIRRQQGRALSLGGGIHHCLGNRLALIEVEAALACLLARLPALRLEQLDTLSWNDRANLRGVDALLASW

>CYP133B3(J162_03331)Xanthomonas_citri_subsp._citri_MN10

MLLSDLATPQFRHDPYPTYARLREEGPLVQVADGRLMSGRYAVVDRLLSDRRVGRDYLQSVRLRYGEAAVHLPLFQGMSRMFLLLNPPLHTQLRGLMTQAFGARQMESMREVASDIAAGLIDAFQANGHCDLLTEFAFPLPIAIICRMLDIAAADVTALSHATSALAKVFDPMMTAEELQATSVAYDQLATYFHGVIAQRRSAGGDDLIARFIQAEDNGRRLSEEEIVSNVILLFFAGHETTSNMICNALVALHRHPQQLRLLQETPGLLPNAVLECMRYDSSVQMATRTALQDFEIEGVAVPRGTMLYLMLGAANHDTLQFTDPQVLDIRRQQGRALSLGGGIHHCLGNRLALIEVEAALACLLARLPALRLEQLDTLSWNDRANLRGVDALLASW

>CYP133B3(J163_03327)Xanthomonas_citri_subsp._citri_MN11

MLLSDLATPQFRHDPYPTYARLREEGPLVQVADGRLMSGRYAVVDRLLSDRRVGRDYLQSVRLRYGEAAVHLPLFQGMSRMFLLLNPPLHTQLRGLMTQAFGARQMESMREVASDIAAGLIDAFQANGHCDLLTEFAFPLPIAIICRMLDIAAADVTALSHATSALAKVFDPMMTAEELQATSVAYDQLATYFHGVIAQRRSAGGDDLIARFIQAEDNGRRLSEEEIVSNVILLFFAGHETTSNMICNALVALHRHPQQLRLLQETPGLLPNAVLECMRYDSSVQMATRTALQDFEIEGVAVPRGTMLYLMLGAANHDTLQFTDPQVLDIRRQQGRALSLGGGIHHCLGNRLALIEVEAALACLLARLPALRLEQLDTLSWNDRANLRGVDALLASW

>CYP133B3(J164_03327)Xanthomonas_citri_subsp._citri_MN12

MLLSDLATPQFRHDPYPTYARLREEGPLVQVADGRLMSGRYAVVDRLLSDRRVGRDYLQSVRLRYGEAAVHLPLFQGMSRMFLLLNPPLHTQLRGLMTQAFGARQMESMREVASDIAAGLIDAFQANGHCDLLTEFAFPLPIAIICRMLDIAAADVTALSHATSALAKVFDPMMTAEELQATSVAYDQLATYFHGVIAQRRSAGGDDLIARFIQAEDNGRRLSEEEIVSNVILLFFAGHETTSNMICNALVALHRHPQQLRLLQETPGLLPNAVLECMRYDSSVQMATRTALQDFEIEGVAVPRGTMLYLMLGAANHDTLQFTDPQVLDIRRQQGRALSLGGGIHHCLGNRLALIEVEAALACLLARLPALRLEQLDTLSWNDRANLRGVDALLASW

>CYP133B3(J172_03343)Xanthomonas_citri_subsp._citri_mf20

MLLSDLATPQFRHDPYPTYARLREEGPLVQVADGRLMSGRYAVVDRLLSDRRVGRDYLQSVRLRYGEAAVHLPLFQGMSRMFLLLNPPLHTQLRGLMTQAFGARQMESMREVASDIAAGLIDAFQANGHCDLLTEFAFPLPIAIICRMLDIAAADVTALSHATSALAKVFDPMMTAEELQATSVAYDQLATYFHGVIAQRRSAGGDDLIARFIQAEDNGRRLSEEEIVSNVILLFFAGHETTSNMICNALVALHRHPQQLRLLQETPGLLPNAVLECMRYDSSVQMATRTALQDFEIEGVAVPRGTMLYLMLGAANHDTLQFTDPQVLDIRRQQGRALSLGGGIHHCLGNRLALIEVEAALACLLARLPALRLEQLDTLSWNDRANLRGVDALLASW

>CYP133B3(J158_03331)Xanthomonas_citri_subsp._citri_UI6

MLLSDLATPQFRHDPYPTYARLREEGPLVQVADGRLMSGRYAVVDRLLSDRRVGRDYLQSVRLRYGEAAVHLPLFQGMSRMFLLLNPPLHTQLRGLMTQAFGARQMESMREVASDIAAGLIDAFQANGHCDLLTEFAFPLPIAIICRMLDIAAADVTALSHATSALAKVFDPMMTAEELQATSVAYDQLATYFHGVIAQRRSAGGDDLIARFIQAEDNGRRLSEEEIVSNVILLFFAGHETTSNMICNALVALHRHPQQLRLLQETPGLLPNAVLECMRYDSSVQMATRTALQDFEIEGVAVPRGTMLYLMLGAANHDTLQFTDPQVLDIRRQQGRALSLGGGIHHCLGNRLALIEVEAALACLLARLPALRLEQLDTLSWNDRANLRGVDALLASW

>CYP133B4(XCC3047)Xanthomonas_campestris_pv._campestris_ATCC_33913

MQLSDFATPAFRQDPYPMYARLRAAGPLVQISDNGWVSGHYTVVDALLSDRRVGRNYLDSIRVRYGANAAEMPLFQGMSRMFLLLNPPVHTQQRALMTKAFGARQLEALREVAVDTADALLDQHEDRRSCDLLNDFAMPMTISLICRMLGLAVTDVAALGQASSALAKVFDPLMRPEDMAQATAAYTTLEQYFRAIVLQRRDTQEDDLIARLIAAEDHGQRMPVDDIVSNVIMLFTAGHETTANMICNALIALHRHPEQLQLLRDTPTLMPNAVLECMRYDSSVQVAMRSVLQPLQVEGTTLPVGAILYLMLGSANHDAEQFTAPQQLDLRRQQGRALSFGGGVHHCLGNRLALIELETALERLLQRAPALRLPELDNLSWNERANLRGIQALHATW

>CYP133B4(XC_1111)Xanthomonas_campestris_pv._campestris_8004

MQLSDFATPAFRQDPYPMYARLRAAGPLVQISDNGWVSGHYTVVDALLSDRRVGRNYLDSIRVRYGANAAEMPLFQGMSRMFLLLNPPVHTQQRALMTKAFGARQLEALREVAVDTADALLDQHEDRRSCDLLNDFAMPMTISLICRMLGLAVTDVAALGQASSALAKVFDPLMRPEDMAQATAAYTTLEQYFRAIVLQRRDTQEDDLIARLIAAEDHGQRMPVDDIVSNVIMLFTAGHETTANMICNALIALHRHPEQLQLLRDTPTLMPNAVLECMRYDSSVQVAMRSVLQPLQVEGTTLPVGAILYLMLGSANHDAEQFTAPQQLDLRRQQGRALSFGGGVHHCLGNRLALIELETALERLLQRAPALRLPELDNLSWNERANLRGIQALHATW

>CYP133B4(XCR_3377)Xanthomonas_campestris_pv._Raphanin

MQLSDFATPAFRQDPYPMYARLRAAGPLVQISDNGWVSGHYTVVDALLSDRRLGRNYLDSIRVRYGANAAEMPLFQGMSRMFLLLNPPVHTQQRALMTKAFGARQLEALREVAVDTADALLDQHEDRRSCDLLNDFAMPMTISLICRMLGLAVTDVVALGQASSALAKVFDPLMRPEDMAQATAAYTTLEQYFRAIVLQRRDTQEDDLIARLIAAEDHGQRMPVDDIVSNVIMLFTAGHETTANMICNALIALHRHPEQLQLLRDTPTLMPNAVLECMRYDSSVQVAMRSVLQPLQVEGTTLPVGAILYLMLGSANHDAEQFTAPQQLDLRRQQGRALSFGGGVHHCLGNRLALIELETALERLLQRAPALRLPELDNLSWNERANLRGIQALHATW

>CYP134B2(Marme_0277)Marinomonas_mediterranea

MSHIETFDVLNPGFVADPYPFYEYLHRSNCIFQDNQTSAYFIGKYDDVKTVLTTPVFTTAPLSVRAQPVMGDRVLAQMEGQEHLHKRKAVLHGLSGKYFKEKYSVLISRVTQKLLQPYLEKGEIDLVLDFGKDYAVLVTLGILGLPSENYQQIAEWHVGVANFITQLNQNELEKMHSLECSRSLRAFLAPIVEERRLSPGSDLISLLCLCDEENAMSTKEIVALCLNILLAATEPADKTLAMLFKCLLDDPELFDVVNRDRKLMRRAIEETLRLHSPVQLIPRQASTDIELSGVLIKKGALVFNMIGAANRDPNIFPEPSKFKLNRKVDQSKVPIKKHHLAFGAGLHICLGAEFSIRQIEITANILMDLLLDMRIPNDFSYVERGLYTRGPESLKLLFNRAPHQGDMYRFSLEVDVGAI

>CYP136F7(S7S_09490)Alcanivorax_pacificus

MSSSVPFSPIPVGSDLKPIPGDPGWPLVGLTLHLMRNPLGIARERYDRYGAASWTNAFGLRMVSLIGPDANEFVLLNKGDLFSNHQGWDYFIGRFFHRGIMLLDFEEHRWHRKIMQQAFKTDVLREYITRMGPGIHKGLDAWQPASGFLMLPAIKQLTLDLATDVFMGHSLGDEADQVNQAFVDTVRAGTAILRFNVPGGRWWRGLRGRRVLEAFFRRELPAKRRSQGHDLFSALCRAETEDGERFSDEDVVNHMIFLMMAAHDTTTITLCNMVYWLARHPEWQQRLRAESLALGKQALEYDDLAQLEGASLVMKEALRLCAPVPSIPRKTVRDAEFRGYHLPAGTLVSISPFFTHHMHEYWPEPERFDPERFAPDRREDKVHPYAWVPFGGGAHKCIGLHFAELQVKAVLHQLLQQFEWSVAPDYEMPLDMTSLPVPSDGLPVVLKRRA

>CYP136F8(B5T_02349)Alcanivorax_dieselolei

MATMTPLAPVPAGSALKPVPGDTGLPLIGNTLPMMRDPVTTLRQRYDRFGPVSWTHLFGLKMVQMLGPDANQFVLMNRGDLFSNHQGWSYFIGPFFHRGIMLLDFEEHRWHRRIMQQAFQREALRGYLQRMAPRTEEGLSHWSEGPIKLLPALKQLTLDLATDVFMGRELGDQTDRINRAFIDTVRAGTSLIRFPVPGLGWSKGLRGRRVLERLFMDDIAAKRAAPDSDLFSMLCQARTEDGHEFSDEDVVSHMIFLMMAAHDTTTITLCTLLYHLAREPQWQQRLREESLALGKPWVEHEDLDKLSGIGMAMKEALRLCAPVPSMPRRTVKDVEYDGFYIPAGTFINIAPFFTHSMEEYWPDPERFDPERFSDARREDRVHPYAWVPFGGGAHKCIGLHFAEMQVKSVLHQMLLRFRWAVPDGYEMPLDTRSLPVPADGLPVTLERL

>CYP151A13(PCA10_49550)Pseudomonas_resinovorans

MSSSAPTTLEAAPYLDVADPSFSMRSEAVAQAREQSWFARTPYGIAVLRYDEVNALIRDQRLRQGSYAWPAHNKATGSFADWWVRMLLSREGADHSRLRRLANPAFSPKLVRKMTPEFQEMANEIIDGFIDAGHCEFVSEFSEPYATQVICSLLGLPRSEWRGLAELAVDMGLALGVTFKQDEAQINAATDKMYGYAKTAVETLKQNGLSEDFLSMLVRANEEDKAALSDQELYDMIILAIFGGIDTTRNQIALAMDTFVQHPDQWKLLGEQPDLARAAVEEVMRVRPTVTWVTREALEDFNYQGLDIKKGTTVHLFSQAAGTDPKAFENPGFDITAKRQPHFGFGAGAHHCIGHFIARGDMTEALSLLAQRLHNPAYDGDVKWLPDSGNTGAISMPMKFDRGA

>CYP151A14(PcP3B5_54780)Pseudomonas_citronellolis

MTTSAPTRLDDAPYLDVSDPSFSIRSQAVMDARAQSWFARTPYGIAVLRYDEVNKLLRDQRLRQGSYAWPAHNNASGSFADWWMRMLLSKEGADHSRLRRLANPAFAPKLVKQMMPDFQRLAGDLIAQFEGRGECEFVSEFAEPYATQVICLLLGLPISQWKGLADLAVEMGLALGVTFKRDEARINAATDKLFGYARQAVEALKRNGLGEDFLSSLVRANEEDKNALSDQELYDMIVLAIFGGIDTTRNQLSLAMDTFLQHPAQWELLGNDAELARAAVEEVMRVRPTVTWVTREALEDFEYQGLHIAKGTTVHLFSQAAGSDPHAFEDASFDITAKRLPHFGFGAGAHHCIGHFIARGDMTAALALLAQHLKHPAPNGEAEWLPDSGNTGATRLPIRFDAVPAK

>CYP152G2(amad1_16670)Alteromonas_mediterranea_DE1

MNLILMYFKLDCYYRKLSVCEVKKRREFFYNTEYFSRTGVAPSRIKKTLFGEGGVQGLDEEEHFQRKKMFMSFLRADKIGKLSMITCDIWKSRVKDWSSKEKINLYQQSCELLTQSVCMWAGVPLKESEVSQRSEELTALFNYAGNIGPKHWKARIARKRNEAWLISIIESIREGTFRPSTNSAAYIVANHRDLDGCLLESQIAAVELNNLLRPTVAVAVYIVYCAHALHQNPLIKHRLVNGSDKDYECFVQEVRRLYPFFPFTAATVKRTFEWRGYKFPKGRRVFLDLFGTNHDARTWENPNNFDMERFRDCKVNDYVFIPQGGGDHFRNHRCPGEWVAIEQMKIATKILVSECDYTVPEQDLDLRMGNLPALPKSNFIISDVRPIDKDEDLNDHI

>CYP152G2(I636_15975)Alteromonas_mediterranea_UM4b

MTGFPKLGILDSTISLIRDPYRYISKHCDELNTDVFQARLLLQKTVCMRGEEAARIFYNTEYFSRTGVAPSRIKKTLFGEGGVQGLDEEEHFQRKKMFMSFLRADKIGKLSMITCDIWKSRVKDWSSKEKINLYQQSCELLTQSVCMWAGVPLKESEVSQRSEELTALFNYAGNIGPKHWKARIARKRNEAWLISIIESIREGTFRPSTNSAAYIVANHRDLDGCLLESQIAAVELNNLLRPTVAVAVYIVYCAHALHQNPLIKHRLVNGSDKDYECFVQEVRRLYPFFPFTAATVKRTFEWRGYKFPKGRRVFLDLFGTNHDARTWENPNNFDMERFRDCKVNDYVFIPQGGGDHFRNHRCPGEWVAIEQMKIATKILVSECDYTVPEQDLDLRMGNLPALPKSNFIISDVRPIDKDEDLNDHI

>CYP152G2(I635_16630)Alteromonas_mediterranea_UM7

MTGFPKLGILDSTISLIRDPYRYISKHCDELNTDVFQARLLLQKTVCMRGEEAARIFYNTEYFSRTGVAPSRIKKTLFGEGGVQGLDEEEHFQRKKMFMSFLRADKIGKLSMITCDIWKSRVKDWSSKEKINLYQQSCELLTQSVCMWAGVPLKESEVSQRSEELTALFNYAGNIGPKHWKARIARKRNEAWLISIIESIREGTFRPSTNSAAYIVANHRDLDGCLLESQIAAVELNNLLRPTVAVAVYIVYCAHALHQNPLIKHRLVNGSDKDYECFVQEVRRLYPFFPFTAATVKRTFEWRGYKFPKGRRVFLDLFGTNHDARTWENPNNFDMERFRDCKVNDYVFIPQGGGDHFRNHRCPGEWVAIEQMKIATKILVSECDYTVPEQDLDLRMGNLPALPKSNFIISDVRPIDKDEDLNDHI

>CYP152R2(UIB01_16925)Pseudomonas_stutzeri_19SMN4

MTDIPRDEHLESSLALLGEGYPFIRDRCQRLHSNLFQTRLLMQNTICLSGQEAARLFYDERYLQRDKAMPRMLKKTLIGEGGVQGLDGEAHRQRKRMFMQLLDSAAVDEVVRLTEQGWCRAIDEWQARSDIELMSEVQSIFTDSVCRWAGVPLPSAELPERRDQLVAMIDGAGGIGARHWAARKARREAEIWLQQLIKQARSGELQAAPTTALMVVAHHRNLDGSRLDSRVAAVELLNLLRPTVAVSYFIIYGALELLAHPQWRERLRSDDAMLEPFAQEVRRLHAFFPFTAARVREGFEWQGYHFPAGTRVMLDLWGTNREASRWSEPDAFQPERFVDWQDNAFSFVTQGGGDPAEGHRCPGERLAIELLKVALRTLTREMEYAVPAQDLRIDLTRMPAKPQSGLLISDVKRLAG

>CYP152R3(CH92_16810)Pseudomonas_stutzeri_28a24

MPDIPHDSQTESSLSLLSEGYSFISSRCQRLGSDLFQVRLLMQNTICMSGEEAAKLFYDEQLFQREHAAPRMLQKTLFGQGGVQGLDGEAHRHRKQLFLSLLTEAAVAELVRLSEANWQAAIETWQQCDRVVFMPEVQAILTRTACDWAGVPLEEGELLHRRDQLAAMIDGAGGVGARHWRARKARKEAESWMIELISRVRAGTLVIDESRPLAVVARHLDLDGAVLKERIAAVEMLNLLRPTVAVARFVTYAALELLAHPQWHQRLQQEDAVLEPFAQEVRRLHAFFPFTAARVRKDFEWRGYHFPKDTRVLLDLYGTNRDARLWVQPEAFRPERFAAWDGGAFNFITQGGGDAASGHRCPGEPLAIALLKSALCMLTRRMSYAVPAQSLHMDPSRMPEQPESLLIICDVRPLSPAG

>CYP152W1(xcc-b100_2293)Xanthomonas_campestris_pv._campestris_B100

MRFTCCCVHRAQRARHHRNKSRQAMTMHASTTDHVGHAAQRLRDDAADATAETQGRIAQAPQRDVLPALLRDGYAFVSRHCDALGSDAFQARLALQQVVFARGPDALATFYHPGRFTRVGAMPPTTLRLLQGRGSVQQLDGDAHLQRKRLFLSVLTPAETSRLVACFEEEWSRQADTWARSAHIILQQEAEHVLCRAACRWAGLPLRAQQSRPLARDLGAMIDGAGAFGPRWVRGWRGRRRVERWVARAVRNVRRAGTAPEASIAATVAWHRDADGVLLSVHTAVTELINLLRPIVAVARWISFCALALHEHPPLRSRLRAGEPGLLQNTVQEVRRFYPFFPLIGGRVRMPFVWRDRHFKQGDWMMVDLYGTNHHPAVWRDPERFDPSRFEHWKGSRYDFVAQGGGSVERDHRCPGENPSIALLMSALSLLASSDYTLPPQDLRYPLNRFPTLPRSGVVLRDFIPPVSVGGVVQSA

>CYP152W1(XCR_2273) Xanthomonas_campestris_pv._Raphanin

MHSSTTDHVGQAAQRLRDDAADATAETEGRIAQAPQSDVLPALLRDGYAFVSRHCEALGSDAFQARLALQQVVFARGPDALATFYHPGRFTRVGAMPPTTLRLLQGRGSVQQLDGDAHLQRKRLFLSVLTPAETSRLVACFEEEWSRQADTWARSAHIILQQEAEHVLCRAACRWAGLPLRAQQSRPLARDLGAMIDGAGAFGPRWVRGWRGRHRMERWVARAVRNVRRAGTAPEASIAATVAWHRDADGVLLSVHTAVTELINLLRPIVAVARWISFCALALHEHPPLRSRLRAGEPGLLQNTVQEVRRFYPFFPLIGGRVRMPFVWRDRHFKQGDWMMVDLYGTNHHPAVWRDPERFDPSRFEQWTGSRYDFVVQGGGSVERDHRCPGENPSIALLMSALSLLASSDYTLPPQDLRYPLNRFPTLPRSGVVLRDFIPPVSVGGVVQSA

>CYP152X1(UIB01_17410)Pseudomonas_stutzeri_19SMN4

MPNIPRDTGLDSTWAFLRDPYRFISTRSKFHQSPVFQTRLILQKTLCLTGAEAARLICDPDRFVRQNAAPKRLQKTLFGQDGVQGLDGDAHRHRKALFMGVLTPANVQELAELSEVRWREYARTWRPGESIVLYEIAREILCRTVCDWAGAPIAERDVQQWTQDLAALYEHAGAIGLQHWQARKARRRLEQWAAELVESTRAAPPTPEQSPLERIAHYKDQHGQPLDLHTASVELLNLLRPTVAVSVFITFAALALHKHPFCLRNLQSGDERDIGCFVQEVRRFYPFFPAISARVKEDFLWEGFSFGRGTLVLLDLYGTNHDSQLWEEADRFKPERFRSNSPSPYCFIPQGPGDPHVNHRCPGEGVAVALMSVAVRFLARSLQYEVPEQDLSITWDRLPALPRSHFVMRNARITM

>CYP152Y1(Q7A_2994) Methylophaga_nitratireducenticrescens

MALMTKLSLDELVQITHAYWLTAINDWQLRDSPIVLKQAAAEVLTQSICQWTGVPLEPNEVKHRTQQFIHMIESASKIGFRHWQGRQARRLMERWCRKLIHQTRTKQLRVEPDKSLYKIAMHQQLDDNLLSEQVAAVELLNILRPTVAITYYIVLTALALHHYPHEAKRLDSDEARHRFVQEVRRFYPFFPATVAEVRKTFEWQGYTFPQGSRVMLDLYGTNHDERLWQNPEQFWPDRFLYNDTDKFSLIPQGGGDYWQHHRCAGEWLTLAMMELALKVLTQEMQYEVPGQNLFLPHNRMPTLPESGFIICKVAPYSVATRVESTIMGRKVHSSP

>CYP153A12(AS19_23510)Alcanivorax_sp._NBRC_101098

MHLQIKALKNLMKVKRKTIGTSRPQVHFVETDLPDVNDVAIEDIDTSNPFLYRQSKSNSYFKRLRDEAPVHYQKDSAFGPFWSITRYEDIVFVDKNHELFSSEPQITLGEFPEGLSVEMFIAMDPPKHDVQRRAVQGVVAPKNLKEMEGLIRKRTGDVLDSLPLDTPFNWVPVVSKELTGRMLASLLDFPYDEREKLVGWSDRLSGASSATGGEFTNEDVFFDDAADMAWSFSKLWRDKEARQKAGEEPGFDLISMLQSNDDTKDLINRPLEFIGNLALLIVGGNDTTRNSMSGGVLAFNQFPEQFEKLKANPKLIPNMVSEIIRWQTPLPHMRRVATQDVELNGQTIKKGDRVLMWYASGNQDERKFENPEQFIIDRKEARNHVAFGYGVHRCMGNRLAELQLRILWEEILPRFEKLEVIGEPERVQSNFVRGYSKMVVKLTAKK

>CYP153A13a(ABO_0201)Alcanivorax_borkumensis

MSTSSSTSNDIQAKIINATSKVVPMHLQIKALKNLMKVKRKTIGTSRPQVHFVETDLPDVNDLAIEDIDTSNPFLYRQGKANAYFKRLRDEAPVHYQKNSAFGPFWSVTRYEDIVFVDKSHDLFSAEPQIILGDPPEGLSVEMFIAMDPPKHDVQRRAVQGVVAPKNLKEMEGLIRKRTGDVLDSLPLDTPFNWVPVVSKELTGRMLASLLDFPYDEREKLVGWSDRLSGASSATGGEFTNEDVFFDDAADMAWAFSKLWRDKEARQKAGEEPGFDLISMLQSNEDTKDLINRPLEFIGNLALLIVGGNDTTRNSMSGGVLALNQFPEQFEKLKANPKLIPNMVSEIIRWQTPLAYMRRVAKQDVELNGQTIKKGDRVLMWYASGNQDERKFENPEQFIIDRKDTRNHVSFGYGVHRCMGNRLAELQLRILWEELLPRFENIEVIGEPERVQSNFVRGYSKMMVKLTAKK

>CYP153A13a(ABO_2288)Alcanivorax_borkumensis

MSTSSSTSNDIQAKIINATSKVVPMHLQIKALKNLMKVKRKTIGTSRPQVHFVETDLPDVNDLAIEDIDTSNPFLYRQGKANAYFKRLRDEAPVHYQKNSAFGPFWSVTRYEDIVFVDKSHDLFSAEPQIILGDPPEGLSVEMFIAMDPPKHDVQRRAVQGVVAPKNLKEMEGLIRKRTGDVLDSLPLDTPFNWVPVVSKELTGRMLASLLDFPYDEREKLVGWSDRLSGASSATGGEFTNEDVFFDDAADMAWAFSKLWRDKEARQKAGEEPGFDLISMLQSNEDTKDLINRPLEFIGNLALLIVGGNDTTRNSMSGGVLALNQFPEQFEKLKANPKLIPNMVSEIIRWQTPLAYMRRVAKQDVELNGQTIKKGDRVLMWYASGNQDERKFENPEQFIIDRKDTRNHVSFGYGVHRCMGNRLAELQLRILWEELLPRFENIEVIGEPERVQSNFVRGYSKMMVKLTAKK

>CYP153A13a(AS19_02080)Alcanivorax_sp._NBRC_101098

MHLQIKALKNLMKVKRKTIGTSRPQVHFVETDLPDVNDLAIEDIDTSNPFLYRQGKANAYFKRLRDEAPVHYQKNSAFGPFWSVTRYEDIVFVDKSHDLFSAEPQIILGDPPEGLSVEMFIAMDPPKHDVQRRAVQGVVAPKNLKEMEGLIRKRTGDVLDSLPLDTPFNWVPVVSKELTGRMLASLLDFPYDEREKLVGWSDRLSGASSATGGEFTNEDVFFDDAADMAWAFSKLWRDKEARQKAGEEPGFDLISMLQSNEDTKDLINRPLEFIGNLALLIVGGNDTTRNSMSGGVLALNQFPEQFEKLKANPKLIPNMVSEIIRWQTPLAYMRRVAKQDVELNGQTIKKGDRVLMWYASGNQDERKFENPEQFIIDRKDTRNHVSFGYGVHRCMGNRLAELQLRILWEELLPRFENIEVIGEPERVQSNFVRGYSKMMVKLTAKK

>CYP153A65(DABAL43B_1552)Psychrobacter_sp._DAB_AL43B

MTKTKIVNAVKIALKTALPSSLTTAIDKIAVQAITKVSTVVPIHIQIKGAHLFQTVKKRALNQSDFPDFIEKPIPEVSTLALADIDLSNPFLFRQHRWQSYYKRLRDEDPVHYQANSPFGAFWSVMRYEDIVFVDKHYELFSSEPVILMGNQPEGLKAEMFIAMDPPKHDIQRQAVQGVVAPKNLLEMEALIRTRTQEVLDSLPIGVEFDWVETVSIELTARMLATLLDFPYEKRRKLVYWSDLAAGAPEMTGGSNNMDETFIAVADMAKQFAELWHDKAARTAAGEAKGFDLITLLQSNDDTKDLVRRPMEFIGNLILLIVGGNDTTRNSMTGGVLALHDFPKQFTKLKNNPSLIPNMVSEIIRWQTPLAYMRRIATQDVELNGKLIKKGDKVVMWYASGNRDERSIDQPNEFIIDRKNARNHLSFGFGVHRCMGNRLAEMQLRILWEELLQRFENIEVVSKPEIVQSNFIRGYSKMMVKLTAKG

>CYP153A66(MARHY2838)Marinobacter_hydrocarbonoclasticus_ATCC_49840

MNIGGRMPTLSKTFDDIQSRVINATSRVVPMHRQIQGLKLLMGAKKKAFGPRRPVPEFVESAIPDVNTLALEDIDVSNPFLYRQDQWRAYFKRLRDEAPVHYQKNSPFGPFWSVTRFEDIMFVDKSHDLFSAEPQIILGDPPEGLSVEMFIAMDPPKHDVQRSSVQGVVAPKNLKEMEELIRSRTGDVLDSLPLGQPFNWVPTVSKELTGRMLATLLDFPYEERHKLVEWSDRMAGAASATGGEYADEEIMFDDAADMAWSFSRLWRDKEARRAAGEEPGFDLISLLQSNDDTKDLINRPMEFIGNLTLLIVGGNDTTRNSMSGGLVAMNEFPKELEKLKAKPELIPNMVSEIIRWQTPLAYMRRVAKQDVELGGQTIKKGDRVVMWYASGNRDERKFEDPDHFIIDRKDARNHMSFGYGVHRCMGNRLAELQLRILWEEILKRFDKIEVVGEPERVQSNFVRGYSELMVQLTPKS

>CYP153A66(Maqu_0600)Marinobacter_hydrocarbonoclasticus_VT8

MPTLPRTFDDIQSRLINATSRVVPMQRQIQGLKFLMSAKRKTFGPRRPMPEFVETPIPDVNTLALEDIDVSNPFLYRQGQWRAYFKRLRDEAPVHYQKNSPFGPFWSVTRFEDILFVDKSHDLFSAEPQIILGDPPEGLSVEMFIAMDPPKHDVQRSSVQGVVAPKNLKEMEGLIRSRTGDVLDSLPTDKPFNWVPAVSKELTGRMLATLLDFPYEERHKLVEWSDRMAGAASATGGEFADENAMFDDAADMARSFSRLWRDKEARRAAGEEPGFDLISLLQSNKETKDLINRPMEFIGNLTLLIVGGNDTTRNSMSGGLVAMNEFPREFEKLKAKPELIPNMVSEIIRWQTPLAYMRRIAKQDVELGGQTIKKGDRVVMWYASGNRDERKFDNPDQFIIDRKDARNHMSFGYGVHRCMGNRLAELQLRILWEEILKRFDNIEVVEEPERVQSNFVRGYSRLMVKLTPNS

>CYP153A67(IMCC21906_02560)Spongiibacter_sp._IMCC21906

MKIVDLVAEKGAASVPMHLQIKAGHMLYKAKNKLGMYTKFPEFVEAPVPEVSTLAIDDIDVSNPFLFKQNRWESYFKRLRDECPVHYQKKSPFGPFWSITRYEDIVYVDKHHDLFSAEPFIIIGATPKELALEMFIAMDPPKHDVQRQAVQGVVAPKNLKEMEGLIRSRTQEVLDQLPLDTPFDWVEDVSIELTARMLATLLDFPYEERRKLVYWSDLAGGGAEMTGGSTNTDELFEGMRDMAKHFSQLWRVKEEKIAAGGEVGFDLIGMLQNNPDTKDLINKPMEFLGNLVLLIVGGNDTTRNSMSGGVLALNRFPDEFAKLKSDPSLIPNMVSEIIRWQTPLAYMRRIAKQDVELNGKTIKKGDKVVMWYASGNRDERAIERPDEFIVDRKGARNHLSFGFGVHRCMGNRLAELQLRILWEELLARFDKIEVVGEPEYVQSNFVKGYSKMMVKLTSK

>CYP153A68(HP15_p187g148)Marinobacter_adhaerens

MKLQTRAMNLSARFVPIQWQVRAAHLAQQALERVGLAPQTPKFSEHPLPDVSSLAIEDVDVSNPFLWRQGLWDAYFRRLRNEKPVHFQQNSAFGPFWSVTRFSDILFVDKRHDLFSAEPIITLGDQPAGLAIETFIAMDPPKHDRQRQAVQGVVAPKNLRELEGLIRQRTQDVLDQLPVDEPIDWVNDVSIELTARMLATLLDFPYDQRRKLVYWSDLAAASPETTGGDVGRDEFFPAVADVARSFSMLWREKEALRDSGEKPGFDVISMMVNNEDTKDLIHRPMEFLGNLALLIVGGNDTTRNSMTGGVLALHKFPKEFDKLKANPALIPNMVSEIIRWQTPLAYMRRVAKKDVELNGEKIRKGDKVVMWYASGNRDERAIDKPDQFIIDRKGARNHLSFGFGVHRCMGNRLAEMQLRILWEEVLARFDQIEVLDEPEYVQSNFVRGYTRMMVKLTKKNQE

>CYP153A69(HDN1F_17560)Gamma_proteobacterium_HdN1

MSTKSSATDVIQAKMINATSRVVPMHLQIQALKMLVKAKKKIIGARRPPLNFVEASVPDVNTLALEDIDLSNPFLYRQDQWRAYFKRLRDEAPVHYQKNSLFGPFWSVTRYEDILFVDKNHELFSSEPQIVLGDPPEGLSVEMFIAMDPPNHDVQRRAVQGVVAPQNLKEMEGLIRTRAGDVLDSLPMGEAFNWVPTVSKELTGRMLATLLDFPYEERHKLVDWSDRLSGAASATGGEFTDEDGMFDDAADMAWSFSRLWRDKEARRAAGEEPGFDLISMLQSSADTKDLINRPMEFIGNLALLIVGGNDTTRNSMSGGVLALNQFPEEFAKLKANPELIPNMVSEIIRWQTPLAHMRRVATQDVEMHGNTIRKGDRVVMWYASGNRDERKFENPDQFIIDRKDARNHMSFGYGVHRCMGNRLAELQLRILWEELLKRFDDIEVVGEPVRVQSNFVRGYSELMVKLTPKQK

>CYP153A70(C427_3047)Paraglaciecola_psychrophila

MNSLTNTTAEPVATSSITPTPAVKFIEQPIADVSTVALEDIDVSNPFMFRQNKWQSYFKRLRDECPVHYQKNSPFGAFWSVTRFEDIMFVDKNHTLFSSEPAIVIGDRPADYMLDMFIAMDPPKHDAQRQAVQSAVAPKNLAEMEELIRERTVDVLNDLPVGESFDWVEKVSVELTTRMLATLFDFPYEKRHKLPYWSDLASGSPEMTGGLVQDDERVAGITDLMTEFSQLWHIKAAQKAAGEQGGFDLISLMQANDNTKNMVDTPLEFLGNLVLLIVGGNDTTRNSMTGGVDALNEFPQEFIKLKNDPSLIPNMVSEIIRWQTPLAHMRRIATEDVELNGKTIKKGDKVVMWYVSGNRDERVINNPDQFVIDRDKARNHLSFGFGIHRCMGNRLAEMQLRILWEEILQRFENIEVINKPKYVQSNFVKGYTELRVKLTAKV

>CYP153A71(B5T_02075)Alcanivorax_dieselolei

MHLQIKALKSLMKAKKKALGSTRPQVKFLERPVPDVNTLALEDIDTSNPFLYRQDQWGAYFKRLRDEAPVHFQKSSQFGPFWSVTRYEDILFVDKNHELFSSEPQIILGDPPEGLSVEMFIAMDPPKHDVQRRAVQGVVAPQNLKEMEGLIRQRAAEVLDSLPLDKAFNWVPAVSKELTGRMLATLLDFPYEQRHKLVDWSDRLSGASSATGGEFTDEDIMFDDAADMAWSFSRLWRDKEARRKAGEPPGFDLISMLQSNKDTRDLINRPMEFIGNLALLIVGGNDTTRNSMSGGVLALNQFPEEFIKLKKNPELIPNMVSEIIRWQTPLAHMRRVATQDVELRGQTIKKGDRVLMWYASGNRDERKFENPDQLIIDRKDARNHISFGYGIHRCMGNRLAELQLRILWEELLKRFDNIEVVGEPERVQSNFVRGYSKLMVKLTAKN

>CYP153A72(S7S_11260)Alcanivorax_pacificus

MPNKQNMTTTQTKIVNATSRIIPMHLQIRALKNVMAAKKKIFGASRPAPNFIEKPVADVTTLKLEDIDLSNPFLYRQNQWDTYAKRLRDEAPVHYQKSSYFGPFWSVMRYEDILFVDKNHQLFSSEPQIVLGDPPDGLSVEMFIAMDPPKHDIQRRAVQSVVAPKNLKEMETLIRGRTEEVLDSLPLGVPFNWVPVVSKELTGRMLATLLDFPYEERHKLVDWSDRLSGAAQATGGEFSDEGAMFDDAADMAQAFSTLWRSKEARRAAGEEPGFDLISLMQSNDDTKDLINRPMEFIGNLALLIVGGNDTTRNSMSGGVLALNQFPEEFTKLKANPDLIPNMVSEIIRWQTPLAHMRRIATQDVEVGGQTIRKGDRVIMWYASGNRDERKFENPDRLVIDRKEARNHIAFGYGIHRCMGNRLAELQLRILWEELLKRFDNIEVVGEPERVQSNFVRGYSTLMVKLTAKD

>CYP153A73(MARHY3773)Marinobacter_hydrocarbonoclasticus_ATCC_49840

MPEFRETPLPPASELALEQIDVSNPFLYRQGLWESYFKRLRDECPVHYQASSPFGPFWSITRYEDILFVDKHHELFSAEPIISIGPQPEGLEIETFIAMDPPRHDVQRQAVQGVVAPKNLAQMESLIRQRTAGVLDQLPVDEPFDWVQRVSIDLTSRMLATLFDFPFDQRHKLAYWSDLATAAPETTGGQVDRDEGFRAAADMARHMSLLWREKEARKAAGKPMGFDLISLLLENEHTRDMINRPMEFIGNMVLLIVGGNDTTRNSMTGGVLALNRFPQEFDRLKQDPSLIPSMVSEIIRWQTPLAYMRRVATQDVELRGQTIRKGDKVVMWYASGNRDERAIEDPDQFRIDRKDARKHLAFGFGVHRCMGNRLAEMQLRILWEELLARFDRIEVVDEPEYVQSNFVRGYTRMNVRLGKRDAAG

>CYP153A74(PS2015_2879)Pseudohongiella_spirulinae

MDSATATSGSPPKTRSLVGRDPWTTPLDEIDLAHPGIWQANEFLPFLDRLRRDDPVHYCAHSAVGPYWSVMKYQDIMAVEANTAVFSSEPTIGIVDVLPEYTLPMFIAMDPPKHDEQRKTVQGVVAPANLKNLEGLIRRRVCTILDNLPLDEEFNWVDRVSRELTTQMLATLFDFPFEDRHKLTYWSDVATAIPGAGLIDSYEESLVILKECLSYFSRLWQERASKPPGNDLISMLAHGPATRNMPPMEFLGNLILLIVGGNDTTRNSISGGVLALNENPDEYQKLRDNPALIPSMVSEIVRWQTPLAYMRRTATQDSELGGKTIRKGDKVLLWYASANRDEDAIEHADRFWIDRPRVRQHLSFGTGIHRCMGNRLAEMQLRILWEEILPRFHKVEVTGEPKRIYSSFVKGYTELPVRLIK

>CYP153A75(IMCC21906_00974)Spongiibacter_sp._IMCC21906

MDSWEQRTNELMECLAYFQELYNERKKLPPKTDLISMLAHSPEMGELSGTDFIGILVLLLVGGNDTTRNSMSGSILAAHLHPQEIDKVKANRALISSMVPEIVRWQTPIAHMRRTAIEDVEFRGQLIRKGDKVAMWYLSGNRDEEVIDRPMDFIVDRPRARHHLSFGFGIHRCLGNRLAEMQLRILWEEFLNRFSGVDVVGDVKRVPSNLVHGYDELPVQLRR

>CYP159B3 (VO64_0375)Pseudomonas_fluorescens_LBUM223

MPPPFRESELNTRDSAHLPPISLTIPQIRLPFAAPPPRSDNEQLRAHALAWAQRYHLIGRRGAHRLSTTPLLELGIALCGRAPTQQAQTLVCWYLWALTLDDRIDDGPWAENGALERFISAVQVVTESDGADPAGETSSFADPMLAVLVDDLWPQTQRWGGAGWRHRLVGHLIQHLRAQAALVRVRETDSALTLAEYLPLRRDSFGALFFFDLIDAAETLDPYEQQAHIEWWNTLREHAADIIAWTNDIHSLAKDVVCGERYNLVSILADTAGMDWPSALESAHQMVNSAVADFTAIAAQRVGQRACAATDPDRLRQVVRAAGDWHQSVSRYHLHAADSTPQNHRQVDLKLTPPTLKSRQFEIDPYPLYELLRTTLPIAYDEPTDVWLVSRHVDVKAALTHPEASNNNYTWQIGPLLGHTIVTMDGCEHAQHRALLSPSFRSKALAALETSIISVTTDLLARMHGRSQVDLIADFTAALPVRVMAHALGLPAETPETVERLKRWCAIGFAYMGNYRQDPALLTGGLSNRDSFYDFIQPYIDARRAEPTDDLISLLLTARIDGQPLSESFVRSYCAILMTAGSETSHGALANLIVNLLNEPGVKEAVLADPGLMDNALTETLRRNPPLQLVLREAREPLHLPSGTIPAGATLACLIGSANRDPDQFADPDTFNMSRTEQATSHFAFGAGRHFCLGSMLARMEITTSARMLLQTFPNLRWAPGFKPIERGFLNRCPEQLEVAL

>CYP159B4 (PFLUOLIPICF704805)Pseudomonas_fluorescens_PICF7

MALCGQAPTQQAETLVCWYLWALILDDRIDDGPWAENGVLERFVTAVQAITENDGADPLDEIGRFDDPMLGVLIDDLWPRTRNWGNERWRHRLVQNLLRHLRAQATLVNMRETGAALALSEYLPLRRDSFGALFFFDLIDAAETLDPYQHSADIEWWSRLREHAADIITWTNDIHSIAKDVVCGERFNLVSILADSAGTDWPASIKAAHQMVNAAVSAFTELAAKHTRQRPSAATDPDRLRQVARAAGDWHRSVSRYHLQANDPTGRINQQVDLNLTPPTLKSRQFEIDPYPLYERLRTTLPIVYDEPTDVWLVSRYADVKAALTHPGASSNNYSWQIGPLLGHTLVAMDGCEHAQHRALLSPSFRSKALEVLEASITSASMDLLAQMQGRHQVDLIADFTCALPVRVMARALGLPAQTTEEVEQLKKWCAIGFAYMGNYRQDPTLLTGGLSNRDRFYDFIQPHIDARRTVPTDDLISHLLAARIDGQPLPEAFVRAYCAILMTAGSETSHGALANLIVNLLDEPGVKEAVMANPDLMDNALAETLRRNPPLQLVLREARESLELPSGTIPCGATFACLIGSANRDPDHFTDPDTFNMSRPHQETNHFAFGAGRHFCLGSILARMEITIGARLLLQTFPGVRWAPGFQPAEHGFLNRCPDRLEVAL

>CYP168A1(PA2475)Pseudomonas_aeruginosa_PAO1

MDDAFSEEGSAQPRHDAQRPALAPRSDGFDIHTYHPDFVADPYPLLRLIRSRAPVCRDQASIWWISRYADVSACLRDRRFSADPARLGAAGVRQGGASWFGHQQLQPLARFYDNFMLFNDAPRHTRLRRLFAPAFGPDAVRRWEARIEVLVEELLDSLLERREPDLLRDFAEPLTIRVAAELFGFPREDTGQLLPWGRDLAAGLDLAASHGDAGQINRSAVAFSDYLQRQARGWSDGSSRPPSGAAPSILDGAAMLEAGLGLEDLVAAYAMVFMAAFETTISMVGNATLALLTHPDQLDLLRRCPELAANAVEELLRFDGAVRGGVRCTLEEVEIGGQRIPPGEKVWLSFLAANRDPEMFAAPDRLQLQRANAKQHVAFAHGPHYCLGAYLARLELQCALRGLVRRRFALASEPTDLRWRRSSVFRTLERLPIVPEGDAQKTCE

>CYP168A1(N297_2545)Pseudomonas_aeruginosa_PAO1-VE13

MDDAFSEEGSAQPRHDAQRPALAPRSDGFDIHTYHPDFVADPYPLLRLIRSRAPVCRDQASIWWISRYADVSACLRDRRFSADPARLGAAGVRQGGASWFGHQQLQPLARFYDNFMLFNDAPRHTRLRRLFAPAFGPDAVRRWEARIEVLVEELLDSLLERREPDLLRDFAEPLTIRVAAELFGFPREDTGQLLPWGRDLAAGLDLAASHGDAGQINRSAVAFSDYLQRQARGWSDGSSRPPSGAAPSILDGAAMLEAGLGLEDLVAAYAMVFMAAFETTISMVGNATLALLTHPDQLDLLRRCPELAANAVEELLRFDGAVRGGVRCTLEEVEIGGQRIPPGEKVWLSFLAANRDPEMFAAPDRLQLQRANAKQHVAFAHGPHYCLGAYLARLELQCALRGLVRRRFALASEPTDLRWRRSSVFRTLERLPIVPEGDAQKTCE

>CYP168A1(N296_2545)Pseudomonas_aeruginosa_PAO1-VE2

MDDAFSEEGSAQPRHDAQRPALAPRSDGFDIHTYHPDFVADPYPLLRLIRSRAPVCRDQASIWWISRYADVSACLRDRRFSADPARLGAAGVRQGGASWFGHQQLQPLARFYDNFMLFNDAPRHTRLRRLFAPAFGPDAVRRWEARIEVLVEELLDSLLERREPDLLRDFAEPLTIRVAAELFGFPREDTGQLLPWGRDLAAGLDLAASHGDAGQINRSAVAFSDYLQRQARGWSDGSSRPPSGAAPSILDGAAMLEAGLGLEDLVAAYAMVFMAAFETTISMVGNATLALLTHPDQLDLLRRCPELAANAVEELLRFDGAVRGGVRCTLEEVEIGGQRIPPGEKVWLSFLAANRDPEMFAAPDRLQLQRANAKQHVAFAHGPHYCLGAYLARLELQCALRGLVRRRFALASEPTDLRWRRSSVFRTLERLPIVPEGDAQKTCE

>CYP168A1(PLES_28211)Pseudomonas_aeruginosa_LESB58

MDDAFSEEGSAQPRHDAQRPALAPRSDGFDIHTYHPDFVADPYPLLRLIRSRAPVCRDQASIWWISRYADVSACLRDRRFSADPARLGAAGVRQGGASWFGHQQLQPLARFYDNFMLFNDAPRHTRLRRLFAPAFGPDAVRRWEARIEVLVEELLDSLLERREPDLLRDFAEPLTIRVAAELFGFPREDTGQLLPWGRDLAAGLDLAASHGDAGQINRSAAAFSDYLQRQARGWSDGSSRPPSGAAPSILDGAAMLEAGLGLEDLVAAYAMVFMAAFETTISMVGNATLALLTHPDQLDLLRRCPELAANAVEELLRFDGAVRGGVRCTLEEVEIGGQRIPPGEKVWLSFLAANRDPEMFAAPDRLQLQRANAKQHVAFAHGPHYCLGAYLARLELQCALRGLVRRRFALASEPTDLRWRRSSVFRTLERLPIVPEGDAQKTCE

>CYP168A1(PAM18_2564)Pseudomonas_aeruginosa_M18

MDDAFSEEGSAQPRHDAQRPALAPRSDGFDIHTYHPDFVADPYPLLRLIRSRAPVCRDQASIWWISRYADVSACLRDRRFSADPARLGAAGVRQGGASWFGHQQLQPLARFYDNFMLFNDAPRHTRLRRLFAPAFGPDAVRRWEARIEVLVEELLDSLLERREPDLLRDFAEPLTIRVAAELFGFPREDTGQLLPWGRDLAAGLDLAASHGDAGQINRSAAAFSDYLQRQARGWSDGSSRPPSGAAPSILDGAAMLEAGLGLEDLVAAYAMVFMAAFETTISMVGNATLALLTHPDQLDLLRRCPELAANAVEELLRFDGAVRGGVRCTLEEVEIGGQRIPPGEKVWLSFLAANRDPEMFAAPDRLQLQRANAKQHVAFAHGPHYCLGAYLARLELQCALRGLVRRRFALASEPTDLRWRRSSVFRTLERLPIVPEGDAQKTCE

>CYP168A1(M062_12875Pseudomonas_aeruginosa_RP73

MDDAFSEEGSAQPRHDAQRPALAPRSDGFDIHTYHPDFVADPYPLLRLIRSRAPVCRDQASIWWISRYADVSACLRDRRFSADPARLGAAGVRQGGASWFGHQQLQPLARFYDNFMLFNDAPRHTRLRRLFAPAFGPDAVRRWEARIEVLVEELLDSLLERREPDLLRDFAEPLTIRVAAELFGFPREDTGQLLPWGRDLAAGLDLAASHGDAGQINRSAAAFSDYLQRQARGWSDGSSRPPSGAAPSILDGAAMLEAGLGLEDLVAAYAMVFMAAFETTISMVGNATLALLTHPDQLDLLRRCPELAANAVEELLRFDGAVRGGVRCTLEEVEIGGQRIPPGEKVWLSFLAANRDPEMFAAPDRLQLQRANAKQHVAFAHGPHYCLGAYLARLELQCALRGLVRRRFALASEPTDLRWRRSSVFRTLERLPIVPEGDAQKTCE

>CYP168A1(PA1R_gp0271)Pseudomonas_aeruginosa_PA1R

MDDAFSEEGSAQPRHDAQRPALAPRSDGFDIHTYHPDFVADPYPLLRLIRSRAPVCRDQASIWWISRYADVSACLRDRRFSADPARLGAAGVRQGGASWFGHQQLQPLARFYDNFMLFNDAPRHTRLRRLFAPAFGPDAVRRWEARIEVLVEELLDSLLERREPDLLRDFAEPLTIRVAAELFGFPREDTGQLLPWGRDLAAGLDLAASHGDAGQINRSAAAFSDYLQRQARGWSDGSSRPPSGAAPSILDGAAMLEAGLGLEDLVAAYAMVFMAAFETTISMVGNATLALLTHPDQLDLLRRCPELAANAVEELLRFDGAVRGGVRCTLEEVEIGGQRIPPGEKVWLSFLAANRDPEMFAAPDRLQLQRANAKQHVAFAHGPHYCLGAYLARLELQCALRGLVRRRFALASEPTDLRWRRSSVFRTLERLPIVPEGDAQKTCE

>CYP168A1(T223_14470)Pseudomonas_aeruginosa_LES431

MDDAFSEEGSAQPRHDAQRPALAPRSDGFDIHTYHPDFVADPYPLLRLIRSRAPVCRDQASIWWISRYADVSACLRDRRFSADPARLGAAGVRQGGASWFGHQQLQPLARFYDNFMLFNDAPRHTRLRRLFAPAFGPDAVRRWEARIEVLVEELLDSLLERREPDLLRDFAEPLTIRVAAELFGFPREDTGQLLPWGRDLAAGLDLAASHGDAGQINRSAAAFSDYLQRQARGWSDGSSRPPSGAAPSILDGAAMLEAGLGLEDLVAAYAMVFMAAFETTISMVGNATLALLTHPDQLDLLRRCPELAANAVEELLRFDGAVRGGVRCTLEEVEIGGQRIPPGEKVWLSFLAANRDPEMFAAPDRLQLQRANAKQHVAFAHGPHYCLGAYLARLELQCALRGLVRRRFALASEPTDLRWRRSSVFRTLERLPIVPEGDAQKTCE

>CYP168A1(SCV20265_2835)Pseudomonas_aeruginosa_SCV20265

MDDAFSEEGSAQPRHDAQRPALAPRSDGFDIHTYHPDFVADPYPLLRLIRSRAPVCRDQASIWWISRYADVSACLRDRRFSADPARLGAAGVRQGGASWFGHQQLQPLARFYDNFMLFNDAPRHTRLRRLFAPAFGPDAVRRWEARIEVLVEELLDSLLERREPDLLRDFAEPLTIRVAAELFGFPREDTGQLLPWGRDLAAGLDLAASHGDAGQINRSAAAFSDYLQRQARGWSDGSSRPPSGAAPSILDGAAMLEAGLGLEDLVAAYAMVFMAAFETTISMVGNATLALLTHPDQLDLLRRCPELAANAVEELLRFDGAVRGGVRCTLEEVEIGGQRIPPGEKVWLSFLAANRDPEMFAAPDRLQLQRANAKQHVAFAHGPHYCLGAYLARLELQCALRGLVRRRFALASEPTDLRWRRSSVFRTLERLPIVPEGDAQKTCE

>CYP168A1(BN889_02704)Pseudomonas_aeruginosa_PA38182

MDDAFSEEGSAQPRHDAQRPALAPRSDGFDIHTYHPDFVADPYPLLRLIRSRAPVCRDQASIWWISRYADVSACLRDRRFSADPARLGAAGVRQGGASWFGHQQLQPLARFYDNFMLFNDAPRHTRLRRLFAPAFGPDAVRRWEARIEVLVEELLDSLLERREPDLLRDFAEPLTIRVAAELFGFPREDTGQLLPWGRDLAAGLDLAASHGDAGQINRSAAAFSDYLQRQARGWSDGSSRPPSGAAPSILDGAAMLEAGLGLEDLVAAYAMVFMAAFETTISMVGNATLALLTHPDQLDLLRRCPELAANAVEELLRFDGAVRGGVRCTLEEVEIGGQRIPPGEKVWLSFLAANRDPEMFAAPDRLQLQRANAKQHVAFAHGPHYCLGAYLARLELQCALRGLVRRRFALASEPTDLRWRRSSVFRTLERLPIVPEGDAQKTCE

>CYP168A1(PADK2_12870)Pseudomonas_aeruginosa_DK2

MDDAFSEEGSAQPRHDAQRPALAPRSDGFDIHTYHPDFVADPYPLLRLIRSRAPVCRDQASIWWISRYADVSACLRDRRFSADPARLGAAGVRQGGASWFGHQQLQPLARFYDNFMLFNDAPRHTRLRRLFAPAFGPDAVRRWEARIEVLVEELLDSLLERREPDLLRDFAEPLTIRVAAELFGFPREDTGQLLPWGRDLAAGLDLAASHGDAGQINRSAAAFSDYLQRQARGWSDGSSRPPSGAAPSILDGAAMLEAGLGLEDLVAAYAMVFMAAFETTISMVGNATLALLTHPDQLDLLRRCPELAANAVEELLRFDGAVRGGVRCTLEEVEIGGQRIPPGEKVWLSFLAANRDPEMFAAPDRLQLQRANAKQHVAFAHGPHYCLGAYLARLELQCALRGLVRRRFALVSEPTDLRWRRSSVFRTLERLPIVPEGDAQKTCE

>CYP168A1(NCGM2_3477)Pseudomonas_aeruginosa_NCGM2.S1

MDDAFSEEGSAQPRHDAQRPALAPRSDGFDIHTYHPDFVADPYPLLRLIRSRAPVCRDQASIWWISRYADVSACLRDRRFSADPARLGAAGVRQGGASWFGHQQLQPLARFYDNFMLFNDAPRHTRLRRLFAPAFGPDAVRRWEACIEALVEELLDSLLERREPDLLRDFAEPLTIRVAAELFGFPREDTGQLLPWGRDLAAGLDLAASHGDAGQINRSAAAFSDYLQRQARGWSDGSSRPPPGAAPSILDGAAMLEAGLGLEDLVAAYAMVFMAAFETTISMVGNATLALLTHPDQLDLLRRCPELAANAVEELLRFDGAVRGGVRCTLEEVEIGGQRIPPGEKVWLSFLAANRDPEMFAAPDRLQLQRANAKQHVAFAHGPHYCLGAYLARLELQCALRGLVRRRFALASEPTDLRWRRSSVFRTLERLPIVPEGDAQKTCE

>CYP168A1(NCGM1900_4055)Pseudomonas_aeruginosa_NCGM_1900

MDDAFSEEGSAQPRHDAQRPALAPRSDGFDIHTYHPDFVADPYPLLRLIRSRAPVCRDQASIWWISRYADVSACLRDRRFSADPARLGAAGVRQGGASWFGHQQLQPLARFYDNFMLFNDAPRHTRLRRLFAPAFGPDAVRRWEACIEALVEELLDSLLERREPDLLRDFAEPLTIRVAAELFGFPREDTGQLLPWGRDLAAGLDLAASHGDAGQINRSAAAFSDYLQRQARGWSDGSSRPPPGAAPSILDGAAMLEAGLGLEDLVAAYAMVFMAAFETTISMVGNATLALLTHPDQLDLLRRCPELAANAVEELLRFDGAVRGGVRCTLEEVEIGGQRIPPGEKVWLSFLAANRDPEMFAAPDRLQLQRANAKQHVAFAHGPHYCLGAYLARLELQCALRGLVRRRFALASEPTDLRWRRSSVFRTLERLPIVPEGDAQKTCE

>CYP168A1(AI22_20650)Pseudomonas_aeruginosa_YL84

MDDAFSEEGSAQPRHDAQRPALAPRSDGFDIHTYHPDFVADPYPLLRLIRSRAPVCRDQASIWWISRYADVSACLRDRRFSADPARLGAAGVRQGGASWFGHQQLQPLARFYDNFMLFNDAPRHTRLRRLFAPAFGPDAVRRWEACIEALVEELLDSLLERREPDLLRDFAEPLTIRVAAELFGFPREDTGQLLPWGRDLAAGLDLAASHGDAGQINRSAAAFSDYLQRQARGWSDGSSSPPSGAAPSILDGAAMLEAGLGLEDLVAAYAMVFMAAFETTISMVGNATLALLTHPDQLDLLRRCPELAANAVEELLRFDGAVRGGVRCTLEEVEIGGQRIPPGEKVWLSFLAANRDPEMFAAPDRLQLQRANAKQHVAFAHGPHYCLGAYLARLELQCALRGLVRRRFALASEPTDLRWRRSSVFRTLERLPIVPEGDAQKTCE

>CYP168A1(G655_12655)Pseudomonas_aeruginosa_B136-33

MDDAFSEEGSAQPRHDAQRPALAPRSDGFDIHTYHPDFVADPYPLLRLIRSRAPVCRDQASIWWISRYADVSACLRDRRFSADPARLGAAGVRQGGASWFGHQQLQPLARFYDNFMLFNDAPRHTRLRKLFAPAFGPDAVRRWEACIEALVEELLDSLLERREPDLLRDFAEPLTIRVAAELFGFPREDTGQLLPWGRDLAAGLDLAASHGDAGQINRSAAAFSDYLQRQARGWSDGSSRPPPGAAPSILDGAAMLEAGLGLEDLVAAYAMVFMAAFETTISMVGNATLALLTHPDQLDLLRRCPELAANAVEELLRFDGAVRGGVRCTLEEVEIGGQRIPPGEKVWLSFLAANRDPEMFAAPDRLQLQRANAKQHVAFAHGPHYCLGAYLARLELQCALRGLVRRRFALASEPTDLRWRRSSVFRTLERLPIVPEGDAQKTCE

>CYP168A1(U769_12850)Pseudomonas_aeruginosa_MTB-1

MDDAFSEEGSAQPRHDAQRPALAPRSDGFDIHTYHPDFVADPYPLLRLIRSRAPVCRDQASIWWISRYADVSACLRDRRFSADPARLGAAGVRQGGASWFGHQQLQPLARFYDNFMLFNDAPRHTRLRRLFAPAFGPDAVRRWEACIEALVEELLDSLLERREPDLLRDFAEPLTIRVAAELFGFPREDTGQLLPWGRDLAAGLDLAASHGDAGQINRSAAAFSDYLQRQARGWSDGSSRPPPGAAPSILDGAAMLEAGLGLEDLVAAYAMVFMAAFETTISMVGNATLALLTHPDQLDLLRRCPELVANAVEELLRFDGAVRGGVRCTLEEVEIGGQRIPPGEKVWLSFLAANRDPEMFAAPDRLQLQRANAKQHVAFAHGPHYCLGAYLARLELQCALRGLVRRRFALASEPTDLRWRRSSVFRTLERLPIVPEGDAQKTCE

>CYP168A1(PA14_32630)Pseudomonas_aeruginosa_UCBPP-PA14

MDDAFSEEGSAQPRHDAQRPALAPRSDGFDIHTYHPDFVADPYPLLRLIRSRAPVCRDQASIWWISRYADVSACLRDRRFSADPARLGAAGVRQGGASWFGHQQLQPLARFYDNFMLFNDAPRHTRLRRLFAPAFGPDAVRRWEACIEALVEELLDSLLERREPDLLRDFAEPLTIRVAAELFGFPREDTGQLLPWGRDLAAGLDLAASHGDAGQINRSAAAFSDYLQRQARGWSDGSSRPPPGAAPSILDGAAMLEAGLGLEGLVAAYAMVFMAAFETTISMVGNATLALLTHPDQLDLLRRCPELVANAVEELLRFDGAVRGGVRCTLEEVEIGGQRIPPGEKVWLSFLAANRDPEMFAAPDRLQLQRANAKQHVAFAHGPHYCLGAYLARLELQCALRGLVRRRFALASEPTDLRWRRSSVFRTLERLPIVPEGDAQKTCE

>CYP168A2(PSPA7_2764)Pseudomonas_aeruginosa_PA7

MDERHTQGLAALGGDALFELPAAQRCIRLGRALAEEVARLAGRAAPVGASLEEAGLASLETLVLAGKVERRLGVRLPASAWQGHQATPERLAQRIVQNLRPEDPATAGSRLGAVALGGWRVAAPLFCLGGAGGAVGYLAALDAALAASRPLVALRSPGLEGEEPPLGSVEEQAARYIQVIKAIQPEGPYLLAGHSYGGIVAYEMAQQLGVRGEPVGALLLIDTLRVENSGDSEPPAAETLAYELGLVQRRLGGAHQSRPLTDNPQLVAVYRSNYAAMERYEPRHYPGPVTLFKAREALPAATLHPQRRTRLYFDDPTLGWGALCPALRVVELEGDHFSLVLPPRAQRLAAAIEEVLGEEATFELGVERLRAAGGLSSRRALEETPEGGLELHPYHPDFLANPYPFLHQLRARAPLYRDREGNWWLTRYADVSACLRDPRFSADPARQSGAGGPGASWFDHQRLQPLARFYDNFMLFNDAPRHTRLKRLFAPAFTPEAVRRWGARIDMLVEELLDAMLERPAPELIQDFAEPLTIRVAAELFDFPREDVGQLLAWGRDLAAGLDLAAVQGDAARINRSAAAFSDYLREQAHGWLQGTARRSSTAPILDGAAMLDAGLALDDLVAAYAMVFMASFETTISMVGNSTLALFDHPGQLERLRREPELLGNAVEELLRYDGAVRSGLRCTLEEVEIGGQRIPAGERVILYFLAANRDPAMFAAPDRLLLDRANARQHLAFAHGPHYCLGAALARLELQGALRALARRRLAPLPQAEGLSWRRSAAFRTLERLPVVAAGPQNTWE

>CYP168B1(HELO_4099)Halomonas_elongate

MDQHFDSVSVKGETAPESTGHATPRETPPPSRPNLREALAQEIVRLTRNNDEDISIPEEYPLNQLGLDSLAAVELTAYIERHFHVKLPLMSLFGSMTLGQLIDLIEKTRTQESAEGPLRTSLGEQSGACAVALHDWQVALPLFCIPGSMGVATYLSSLCTELAGLASTIAFQSPGIDGSESPLGSVEELARRYIAEMQTIQPEGPYRLAGHSFGGLVAHEMACQLHERGERIEALFLIDTFQVRSIGEANDATTSDLMALYELHNIIQRLSDRLDDEPIRVSELEALPPEAQRELLTRRLGTRFNSLHRVATVHHANYMAMERFQPRYYPGPATLLCARSEFPAQLVHPARSLHFCTDEPDLGWQGLCASLNVITVPGDHLTMVRPPHVQALVEAMRPAMDAQSRLSLGMDRLLPARPPRAPGRALEISRHGISFDPYHPDHVDDPYPFLSQLRDCGPVIKDTVSRWWLTRHAEVSAGLRDKRFGVDPRGLAETLPHLDASSASFPFLSALSRQQEEVPFSQHLNRFMLFLDPPQHQQLRRVFSPLFTPEAVKHWTGYIDECAAELIGNLRQDREADLIKELALPLPAAAISEILGFPREDVPEVLPWGQDMISGFDPLMSDDTAARINRSAEEFSRYIREHLETQRKVKSGPGILDPNTALDQGLSIEELVTHYALMFAVGFETTTDMIGNSALALLRHPDQLERWQAQPEISDNAVEELLRYDGPVRCSIRYALEDLDFGGRRIRRGEMVVFSFSSANRDPQAFPEPDRLDLGRDARRHVAFAHGAHYCLGAHLARIELRRVLPALIQHDFSLAPGGTQWRPSLVFRGLETLRIRNH

>CYP168C1(LG3211_5249)Lysobacter_gummosus

MERADPGVNIKGSVGESDIDVANGERRLGAIERRREIAEDIAGEVGRLTRAKEVALRPDTLLSDVGIDSLGSMELLGYIERKFGVAIPISTLLGSTSFDELVTKIEQLQGSAGAVDGKIHSAELLHPRAWDRKARATLLRDWRIAAPLFLIPGLNGTTYYLSSLCKALHTNRACIAFQMPGVDGLEPPLGSIEEIARRHVDEMRAIQPHGPYAIAGHSFGGVVAYEMAQMLAEQGQQVAPLMLLDSPNSESEDEALQDDEVMALFEVIGVYCRFSERPLKPIRAERLSGLPVDEQLQLLWSLLASYPTAAHVIATYRKGFVAMTRYRPRPYGGPVILFRSAEGFPAEAMHPERRVRSQFDSSTLGWGGLCADLRIVETPGDHFSMVMPPHSAELGAAMQEPLNTAADMLIDFDRLRPAAVVTKVGRALRVDGARVHFDPHHPDFREDPYPFLNQLREHTPIFQDALSQWWVTRHADVSAGLRNRLLSVDARAIDHVEGIGSESARPSALSSWFRNQDASALAQLYNKFLLFIDAPRHTVLRKVFSPSFSHESIRGLADCIDERVETLMADMRAKSQPDLMRDLALPLPVGIISLIYGVPDADSAQVTQWARDLAAGLDSGMSLQAMRKAERSAEEFTRYLHGHVQRLRKTPPHTPGGARLDVNDAIAQGITPDELVAHIAMSYLAGFETTTNSIGNGALALLRHPDQLERLRSDPGLAENASEELLRYDCPVMFVMRFALEDLEVAGQRIPRGSSITFMLASANRDPAAFHDPDRLDLARSARHHVAFSNGAHYCLGAPLARLELQRVFVALSRQRFQPVPGGLAWRDAYTFRALERFPIAWC

>CYP169A1(N297_3803)Pseudomonas_aeruginosa_PAO1-VE13

MQQTIDCPIRRRLAHLPWANDGRAGVRHWLEMQRDPLAWLQKMHVAQPDLAVARMGPQRLWCLFHPQAVQELMVDRRDDLQRWQPALCMLKQWNGRSFMMREGAPAQARRKEVRPHLAPPPASEVRRLAAEWGERVEEGREYDLDLEMAAFSVTLSGHALFDVDLQPSAYRIAKAVRLLSRVALLEMSTGLPLGHWFPSKLCPRKRWALGQLREAVGEVAERSPRPLADLRDELCTLLMASHQSTGVTLTWSLLLLAQRPELLARLRAELAGVNWTAIRSVADLRDCALLRAVLQECLRLYPPAYGLAPRQVTADIEVFGQRLKRGDVTMVSSWITQRDPRWFEAPLEFRPERFLEPARWPRGAYFPFGLGDRACPGTAMAMIDLAAALAYWVEHWDIMHDGDLAPRGWFSLRPQRARVRFRRRA

>CYP169A1(N296_3803)Pseudomonas_aeruginosa_PAO1-VE2

MQQTIDCPIRRRLAHLPWANDGRAGVRHWLEMQRDPLAWLQKMHVAQPDLAVARMGPQRLWCLFHPQAVQELMVDRRDDLQRWQPALCMLKQWNGRSFMMREGAPAQARRKEVRPHLAPPPASEVRRLAAEWGERVEEGREYDLDLEMAAFSVTLSGHALFDVDLQPSAYRIAKAVRLLSRVALLEMSTGLPLGHWFPSKLCPRKRWALGQLREAVGEVAERSPRPLADLRDELCTLLMASHQSTGVTLTWSLLLLAQRPELLARLRAELAGVNWTAIRSVADLRDCALLRAVLQECLRLYPPAYGLAPRQVTADIEVFGQRLKRGDVTMVSSWITQRDPRWFEAPLEFRPERFLEPARWPRGAYFPFGLGDRACPGTAMAMIDLAAALAYWVEHWDIMHDGDLAPRGWFSLRPQRARVRFRRRA

>CYP169A1(M801_3668)Pseudomonas_aeruginosa_PAO581

MQQTIDCPIRRRLAHLPWANDGRAGVRHWLEMQRDPLAWLQKMHVAQPDLAVARMGPQRLWCLFHPQAVQELMVDRRDDLQRWQPALCMLKQWNGRSFMMREGAPAQARRKEVRPHLAPPPASEVRRLAAEWGERVEEGREYDLDLEMAAFSVTLSGHALFDVDLQPSAYRIAKAVRLLSRVALLEMSTGLPLGHWFPSKLCPRKRWALGQLREAVGEVAERSPRPLADLRDELCTLLMASHQSTGVTLTWSLLLLAQRPELLARLRAELAGVNWTAIRSVADLRDCALLRAVLQECLRLYPPAYGLAPRQVTADIEVFGQRLKRGDVTMVSSWITQRDPRWFEAPLEFRPERFLEPARWPRGAYFPFGLGDRACPGTAMAMIDLAAALAYWVEHWDIMHDGDLAPRGWFSLRPQRARVRFRRRA

>CYP169A1(PAM18_1267)Pseudomonas_aeruginosa_M18

MQQTIDCPIRRRLAHLPWANDGCAGVRHWLEMQRDPLAWLQKMHVAQPDLAVARMGPQRLWCLFHPQAVQELMVDRRDDLQRWQPALCMLKQWNGRSFMMREGAPAQARRKEVRPHLAPPPASEVRRLAAEWGERVEEGREYDLDLEMAAFSVTLSGHALFDVDLQPSAYRIAKAVRLLSRVALLEMSTGLPLGHWFPSKLCPRKRWALGQLREAVGEVAERSPRPLADLRDELCTLLMASHQSTGVTLTWSLLLLAQRPELLARLRAELAGVNWTAIRSVADLRDCALLRAVLQECLRLYPPAYGLAPRQVTADIEVFGQRLKRGDVTMVSSWITQRDPRWFEAPLEFRPERFLEPARWPRGAYFPFGLGDRACPGTAMAMIDLAAALAYWVEHWDIMHDGDLAPRGWFSLRPQRARVRFRRRA

>CYP169A1(M062_19455)Pseudomonas_aeruginosa_RP73

MQQTIDCPIRRRLAHLPWANDGRAGVRHWLEMQRDPLAWLQKMHVAQPDLAVARMGPQRLWCLFHPQAVQELMVDRRDDLQRWQPALCMLKQWNGRSFMMREGAPAQARRKEVRPHLAPPPASEVRRLAAEWGERVEEGREYDLDLEMAAFSVTLSGHALFDVDLQPSAYRIAKAVRLLSRVALLEMSTGLPLGHWFPSKLCPRKRWALGQLREAVGEVAERSPRPLADLRDELCTLLMASHQSTGVTLTWSLLLLAQRPELLARLRAELARVNWTAIRSVADLRDCALLRAVLQECLRLYPPAYGLAPRQVTADIEVFGQRLKRGDVTMVSSWITQRDPRWFEAPLEFRPERFLEPARWPRGAYFPFGLGDRACPGTAMAMIDLAAALAYWVEHWDIMHDGDLAPRGWFSLRPQRARVRFRRRA

>CYP169A1(SCV20265_1331)Pseudomonas_aeruginosa_SCV20265

MQQTIDCPIRRRLAHLPWANDGRAGVRHWLEMQRDPLAWLQKMHVAQPDLAVARMGPQRLWCLFHPQAVQELMVDRRDDLQRWQPALCMLKQWNGRSFMMREGAPAQARRKEVRPHLAPPPASEVRRLAAEWGERVEEGREYDLDLEMAAFSVTLSGHALFDVDLQPSAYRIAKAVRLLSRVALLEMSTGLPLGHWFPSKLCPRKRWALGQLREAVGEVAERSPRPLADLRDELCTLLMASHQSTGVTLTWSLLLLAQRPELLARLRAELARVNWTAIRSVADLRDCALLRAVLQECLRLYPPAYGLAPRQVTADIEVFGQRLKRGDVTMVSSWITQRDPRWFEAPLEFRPERFLEPARWPRGAYFPFGLGDRACPGTAMAMIDLAAALAYWVEHWDIMHDGDLAPRGWFSLRPQRARVRFRRRA

>CYP169A1(BN889_04070)Pseudomonas_aeruginosa_PA38182

MTDSAAPRLHVQALSADCAEAARRWAERLGLPLAADDEAEFAVQVGEQGLQVLQLGPDSPGPVRVDFVEGASAHRRKFGGGSGQMIAKAVGVQPGIRPRVLDATAGLGRDGFVLASLGCEVTLVERQPLIAALLEDGLERARRDPDVAPIAARMRLLGGNSADLMRAWDGEAPQVIYLDPMFPHRDKSALVKKEMRLFRPLVGDDLDAPALLQAALALASHRVVVKRPRKAPIIEGPKPGYSLEGKSSRYDIYPKKALGKAECRHRREAASMQQTIDCPIRRRLAHLPWANDGRAGVRHWLEMQRDPLAWLQKMHVAQPDLAVARMGPQRLWCLFHPQAVQELMVDRRDDLQRWQPALCMLKQWNGRSFMMREGAPAQARRKEVRPHLAPPPASEVRRLAAEWGERVEEGREYDLDLEMAAFSVTLSGHALFDVDLQPSAYRIAKAVRLLSRVALLEMSTGLPLGHWFPSKLCPRKRWALGQLREAVGEVAERSPRPLADLRDELCTLLMASHQSTGVTLTWSLLLLAQRPELLARLRAELARVNWTAIRSVADLRDCALLRAVLQECLRLYPPAYGLAPRQVTADIEVFGQRLKRGDVTMVSSWITQRDPRWFEAPLEFRPERFLEPARWPRGAYFPFGLGDRACPGTAMAMIDLAAALAYWVEHWDIMHDGDLAPRGWFSLRPQRARVRFRRRA

>CYP169A1(AI22_26890)Pseudomonas_aeruginosa_YL84

MQQTIDCPIRRRLAHLPWANDGRAGVRHWLEMQRDPLAWLQKMHVAQPDLAVARMGPQRLWCLFHPQAVQELMVDRRDDLQRWQPALCMLKQWNGRSFMMREGAPAQARRKEVRPHLAPPPASEVRRLAAEWGERVEEGREYDLDLEMAAFSVTLSGHALFDVDLQPSAYRIAKAVRLLSRVALLEMSTGLPLGHWFPSKLCPRKRWALGQLREAVGEVAERSPRPLADLRDELCTLLMASHQSTGVTLTWSLLLLAQRPELLARLRAELARVNWTAIRSVADLRDCALLRAVLQECLRLYPPAYGLAPRQVTADIEVFGQRLKRGDVTMVSSWITQRDPRWFEAPLEFRPERFLEPARWPRGAYFPFGLGDRACPGTAMAMIDLAAALAYWVEHWDIMHDGDLAPRGWFSLRPQRARVRFRRRA

>CYP169A1(PA14_16780)Pseudomonas_aeruginosa_UCBPP-PA14

MQQTIDCPIRRRLAHLPWANDGRAGARHWLEMQRDPLAWLQKMHVAQPDLAVARMGPQRLWCLFHPQAVQELMVDRRDDLQRWQPALCMLKQWNGRSFMMREGAPAQARRKEVRPHLAPPPASEVRRLAAEWSERVEEGREYDLDLEMAAFSVTLSGHALFDVDLQPSAYRIAKAVRLLSRVALLEMSTGLPLGHWFPSKLCPRKRWALGQLREAVGEVAERSPRPLADLRDELCTLLMASHQSTGVTLTWSLLLLAQRPELLARLRAELAGVNWTAIRSVADLRDCALLRAVLQECLRLYPPAYGLAPRQVTADIEVFGQRLKRGDVTMVSSWITQRDPRWFEAPLEFRPERFLEPARWPRGAYFPFGLGDRACPGTAMAMIDLAAALAYWVEHWDIMHDGDLAPRGWFSLRPQRARVRFRRRA

>CYP169A1(PLES_13051)Pseudomonas_aeruginosa_LESB58

MQQTIDCPIRRRLAHLPWANDGRAGVRHWLEMQRDPLAWLQKMHVAQPDLAVARMGPQRLWCLFHPQAVQELMVDRRDDLQRWQPALCMLKQWNGRSFMMREGAPAQARRKEVRPHLAPPPASEVRRLAAEWGERVEEGREYDLDLEMAAFSVTLSGHALFDVDLQPSAYRIAKAVRLLSRVALLEMSTGLPLGHWFPSKLCPRKRWALGQLREAVGEVAERSPRPLADLRDELCTLLMASHQSTGVTLTWSLLLLAQRPELLARLRAELARVNWTAIRSVADLRDCALLRAVLQECLRLYPPAYGLAPRQMTADIEVFGQRLKRGDVTMVSSWITQRDPRWFEAPLEFRPERFLEPARWPRGAYFPFGLGDRACPGTAMAMIDLAAALAYWVEHWDIMHDGDLAPRGWFSLRPQRARVRFRRRA

>CYP169A1(U769_06495)Pseudomonas_aeruginosa_MTB-1

MQQTIDCPIRRRLAHLPWANDGRAGARHWLEMQRDPLAWLQKMHVAQPDLAVARMGPQRLWCLFHPQAVQELMVDRRDDLQRWQPALCMLKQWNGRSFMMREGAPAQARRKEVRPHLAPPPASEVRRLAAEWGERVEEGREYDLDLEMAAFSVTLSGHALFDVDLQPSAYRIAKAVRLLSRVALLEMSTGLPLGHWFPSKLCPRKRWALGQLREAVGEVAERSPRPLADLRDELCTLLMASHQSTGVTLTWSLLLLAQRPELLAGLRAELAGVNWTAIRSVADLRDCALLRAVLQECLRLYPPAYGLAPRQVTADIEVFGQRLKRGDVTMVSSWITQRDPRWFEAPLEFRPERFLEPARWPRGAYFPFGLGDRACPGTAMAMIDLAAALAYWVEHWDIMHDGDLAPRGWFSLRPQRARVRFRRRA

>CYP169A1(T223_06425)Pseudomonas_aeruginosa_LES431

MQQTIDCPIRRRLAHLPWANDGRAGVRHWLEMQRDPLAWLQKMHVAQPDLAVARMGPQRLWCLFHPQAVQELMVDRRDDLQRWQPALCMLKQWNGRSFMMREGAPAQARRKEVRPHLAPPPASEVRRLAAEWGERVEEGREYDLDLEMAAFSVTLSGHALFDVDLQPSAYRIAKAVRLLSRVALLEMSTGLPLGHWFPSKLCPRKRWALGQLREAVGEVAERSPRPLADLRDELCTLLMASHQSTGVTLTWSLLLLAQRPELLARLRAELARVNWTAIRSVADLRDCALLRAVLQECLRLYPPAYGLAPRQMTADIEVFGQRLKRGDVTMVSSWITQRDPRWFEAPLEFRPERFLEPARWPRGAYFPFGLGDRACPGTAMAMIDLAAALAYWVEHWDIMHDGDLAPRGWFSLRPQRARVRFRRRA

>CYP169A1(M802_3800)Pseudomonas_aeruginosa_c7447m

MQQTIDCPIRRRLAHLPWANDGRAGARHWLEMQRDPLAWLQKMHVAQPDLAVARMGPQRLWCLFHPQAVQELMVDRRDDLQRWQPALCMLKQWNGRSFMMREGAPAQARRKEVRPHLAPPPASEVRRLAAEWGERVEEGREYDLDLEMAAFSVTLSGHALFDVDLQPSAYRIAKAVRLLSRVALLEMSTGLPLGHWFPSKLCPRKRWALGQLREAVGEVAERSPRPLADLRDELCTLLMASHQSTGVTLTWSLLLLAQRPELLARLRAELARVNWTAIRSVADLRDCALLRAVLQECLRLYPPAYGLAPRQVTADIEVFGQRLKRGDVTMVSSWITQRDPRWFEAPLEFRPERFLEPARWPRGAYFPFGLGDRACPGTAMAMIDLAAALAYWVEHWDIMHDGDLAPRGWFSLRPQRARVRFRRRA

>CYP172B1(GU3_14670)Oceanimonas_sp._GK1

MTCPVFPKPAKSKASLWKVFFTKRHSWLDALYERSYGMKMGEYKLPGLTLYMVNQPDLVRQVMVQSMADFPKHRMLGDILEPLLGESIFTTNGEQWQKQRDMLDPAFKHARVQQVFGLMQSAANDMLERLKTHRPGQDIDPEMTFVTADIIFRTIMSTRLNEEQANRILDAFVRFQAESPKLALMKMFRLPGFLQRGGSERRRMAAAKEIRGTIEDIIRPRYQQAEAARAGCPRSKAELENQQDILSSLLLTTDASTGQPFGFDEIVDQISMLFLAGHETTASSLTWSLYLLATHPQIQEDAYQEVTQVLNGQPISVEALRKMVLVRDVFREALRLYPPVGFFARECAHATEMRNKHMKAGSTVMVSPWLIHRHKDYWHNPHQFDPYRFTAKQLKTPLSKSYLPFGAGPRVCIGAAFAQQESSLILASILQHYQLSLAEGFEPKPVGRLTIRSDNGLQLVLTPREQQP

>CYP198A1(XCC2912)Xanthomonas_campestris_pv._campestris_ATCC_33913

MRRPAFPASVMNSSGAVWQHKRRTLMPAFRAALVRESAMQASAATRSLLHELGDSCATQDMRTLMTGLCAQLGAGFLLGDSANAADLLRMLPMVDAISKQTRRQSLAPTWWPSSGRRRLRRLRADIDMALDRILMQSTQRPPRAASVLALLLAETARDDGDWCRDEAAAILMSALEPMSAALTWTLLLLAQHPHIAQEVAQEASALDGADVASGTSLLDRLPQSRACVKESMRLYPPAWITARIAQRDATLNGFHVPRGTQLLVSAWVVHRDGRHFPDPEIFLPARWLDDSATHSLTRYSYFPFGGGPRSCIGCMLALTQMTIVIATVLHACSLHLAPDARPSPFPALVLRPMDVRIALRPRVIRSVVPSRAHASPVRLASVTPND

>CYP198A1(XC_1197)Xanthomonas_campestris_pv._campestris_8004

MRRPAFPASVMNSSGAVWQHKRRTLMPAFRAALVRESAMQASAATRSLLHELGDSCATQDMRTLMTGLCAQLGAGFLLGDSANAADLLRMLPMVDAISKQTRRQSLAPTWWPSSGRRRLRRLRADIDMALDRILMQSTQRPPRAASVLALLLAETARDDGDWCRDEAAAILMSALEPMSAALTWTLLLLAQHPHIAQEVAQEASALDGADVASGTSLLDRLPQSRACVKESMRLYPPAWITARIAQRDATLNGFHVPRGTQLLVSAWVVHRDGRHFPDPEIFLPARWLDDSATHSLTRYSYFPFGGGPRSCIGCMLALTQMTIVIATVLHACSLHLAPDARPSPFPALVLRPMDVRIALRPRVIRSVVPSRAHASPVRLASVTPND

>CYP198A2(SB85_08940)Xanthomonas_sacchari

MTDARPRPPGPRGHWAFGNRQAFAADPLAFLQDCAREHGDVVRIAERTYLIAAPAAIASVLGDDGSLYAKSDPDPRARRAAFPASVMNSEGEAWRHKRQALQPAFRASLVRDYAGQALAATQALLQSSSDSAQAPDLRLTMTALCAQLGAGFLLGDPAHAPALLRMLPMVDAILQQTRTPSAAPAWWPSAAKRRLRAARGELDTTLTQILTSPRSNGADASVLELLRRNDPQGENDWCRDEAAAMLMSALEPMAAGLTWTLLLLAQHPSIAQAVAEEADALPGIDGASTTPCADALVERLPLTRACVKEAMRLYPPAWMTARIAQRDTTLGGFAVPRGTQLIVSQWVVQRDPRHFAAPDRFLPARWLDPAQTPARYTYFPFGGGPRSCIGSQLALVQMTLVVAGLLRERTLHLATDARPRPYPALVLRPLDVRIALRPRTTTLSRQSPATAPFPRTPETPHG

>CYP221A1(Pfl01_2861)Pseudomonas_fluorescens_Pf0-1

MSDPLLKLLQKPLFDPEKRHRVSLREYMDLNIDRMRAIIGNGLMTNAMWLSQPRQSEFRLMLERAALIGAVDYSLLACIVDHFIAGDAFFAHGSQHQIAQYHQEICQLKAVYAFGCTEIASGSDVANLQTTINYDPHKHCLILNSPTPQSCKFWIGNALHAAVVVMVLGRLIVKGVDEGLHWFRVRIREQENGPLLPGVRITTCDPKGGIHANQVAGIRFCNMKLPLDALMQRYARFSAQGVFSSEIPPKERLKSAMQTFIQERLFLIAGARGAASMCVYLAYRFACHRLVKGNEGSQSLLTKALFRQRLYAEQLKVLALKLLEQAVLSRFEACWHQPARRKELHILAAVVKSVGTWLGLEVMSACRELCGSQGFHHHNRIVTLVMDHGISTTFAGDNNILCCQVARDAINRPRFANENIAQRIESLIVDQCRRAGDFSHRQAVALTYARALDLIINEGKHHPLVTSEIFEDIVHVFSPKLYEWELVASTKLEQNATEAQLILLNELLKPPSELVRAPIDKKNYVKHFTKPLYDNKPDFSNRNTIRNPYRAYTWLRKHQPVYWCEHLQAWFLTRYCDVIAAQADSRRFSSNRMQQLIDARVPENKRTHLNEFIKLASRWMYSQDGDTHKASRHLLGNAFTPRSIEALRAIIQDITDRELSRLHGQTDLKTALFDRVPALILARLYGMKDDEALRLRRWTRDIVMFLGGSQDADQGPDQALEGIKEMYACFAELIEQRRRQPGDDLVSRVLESGQNSAASLDEVLAQIVFILVAGYTTSADQMCLGLLHLLKHPQQLEALLADPTLIGSFIEEMLRFDPAGSLSHRILMEDVTIDNITMKKGNLVYLIRASANRDPEKFHAPNTFDIRRARNEHLTFGKGEHFCMGTSLFRLEAEIVFTSLLKRFPDLQLIARRPAKWRNSNLQFRGLKTLPVDLGTGV

>CYP221A4(AA957_29280)Pseudomonas_trivialis

MALLSAPDFSDPKTIINPYPAFARLREHHPVYWSEHHKAWLLTRYGDVSSAQADARRYSSNRMRQLVDAQLSPEKRAALEPFVEKASRWMYSQDGKEHEAGRKVLGKTFSPGSIEALGEAIQTIIDDQLKQLSPRPEMMDELFNKIPALILAYLFDIPANDALKIRGWTDAIIVCMVGSTDPAYGPKEALQAMEEMYAYFSRLIGRRRLAPGNDLVSQVIAAGDKASMSEEDFLAQLAFILVAATTTSADQLGIILFYLLEKPKRWAAVRDDPDKVDAAIEEALRICPAGQLSHRVLTEDVVLHGKTMRKGELVFLIRAAANRDPAHFAHPDRFDLYRQKQDHLAFGRGPHYCMGRLLFKLEAKILFTTLLRRFPHMHLIKGRPPRWRDNSLQFRGLGRIEVELAPVTDVITRCFSAAPWEKKGGYCRALRVGNLIMTSGTVSFDAQGKPFAEHDAYLQTQRCLEIIETALKQLGTDRTRVIATRMYTTDMELWQKILKAHKAFFDGCEPTTMLLSVKALIAPEFLIEIEAQAMVAQS

>CYP226A10(RK21_01256)Pseudomonas_plecoglossicida

MTIKPEKIMSTSEHNELTKAFNDVASNYRGTSDIDLHATYREMRANSPVLRDNFMARLGVPSIAGLDATRPTFTLFKYDDVMAVMRDAGNFTSGFIAEGLGAFFDGLILTAMDGEAHKSIRSLLQPVFMPDTVNRWKETRIDRVIRDEYLKPMVPARSADLMDFALYFPIRVIYSLIGFPEDRPEQIEQYAAWALAILAGPQVDPEKAAAARGAAMEAAQALYDVVKEVVAERRAQGGTGDDLISRLIQAEYQGRSLDDHEIATFVRSLLPAASETTTRTFGTLMSLLLQHPGVLERVRNDRSLVNKAIDEAVRFEPVATFKVRQAAKDLQIRDVPIPQGAMVQCIVTSANRDEDAFENPDVFDIDRKPKPSFGFGFGPHMCIGQFVAKTEINCALNAILDLMPNIRLDPNKPAPEIVGAQLRGPHYLHVLWD

>CYP226A13(Glaag_0363)Glaciecola_sp._4H-3-7+YE-5

MSKQQNENLEKAFAGVADNYRGQDVDLNAIYREMRKNSPIIAEDFMSQQGVPNIAGLDANRLTFTLFKHKDVMTVLRDAKNFTSGFIAEGLGAFFDGLILTGMDGEEHKKARALLQPVFMPDVVNTWRDTKMDPIVRNEFLIPLQKEGKADLMDFALYFPIRLIYSLIGFPEDDHDKVKQVAAWSLAILAGPQVTPEKAAEARKAAMEAAKCLYDAVKEAVVEVRKNGAQGGDLISRLIRAEYEGRQLDDHEITTFVRSLLPAAGETTTRTFGSLMTLLLERPALLERVRADRSLVGKAIDEAVRLEPVATFKVRQAAEDLEIRGMQIPKGAMVQCIVASANRDEEVFEDSEKYIIERKVKPSFGFGFGPHMCIGQFIAKTEMVVALNAILDLFPNIRLDPDMPKPKIEGAQLRGPHEVHVVWD

>CYP226A14(HP15_18)Marinobacter_adhaerens

MTLTANNLDIEAAYHAVSDTYLGSEVDIHELCREKRHNEPVMEGDFVDTYLKVPTNAGAKGGKCAVTLFKYKDILSVLRDGETFTNGFIAEGLGAFFDGLIVLAMDGEQHRRTRALLQPIFMPQTVNTWKPEIERVIRDEFLTPLVATKGTNLMDFGLYFPIRVMYALMGFPTDDTEKFKKYASWALALVAANQIDPEKAKIFGPIAGQAVKSLYDSINEVVVKTRAEGAEGNGLISRLINAEYEGRALDDHEVTTFVRSLLPAAGETTTRTFSSIMTLLLERPDLLERVKNDRSLISKLIDESVRFEPVSTFKVRQASKDVEIGGVKVPKGALVQCMVISANRDEDIFPEPDTFDIDRQARPSLGFGFGPHMCIGQFVAKVELNSAINAILDLFPGIRLDPSKPAPKIAGAQLRGAKAIHVIWD

>CYP226A15(HP15_51)Marinobacter_adhaerens

MSELSKNELENVFEDVASNYRGADIDLHAAYAQMRKESPVLPENFMEKLGVPSIAGVDPDRPCYTLFKYDDVMRVMRDSTLFTSGFIAEGLGAFFDGLILTAMDGEEHKKMRNLLQPVFMPDTVNRWKADRIDRVIREEFLEPMVADKQADLMDFALYFPIRVIYSLIGFPEDRPEEIKQYAAWALAILKGPQVDPAKAEAAKKEAMEAVQALYGAIREVVEQRRAEGGEGDDLISRLIVAEYEGESLDDHQITTFVRSLLPAAGETTTRTFGTLMTLLLERPELLARIREDRSLVNKAIDEAVRFEPVATFKVRQAAQDTEIRGVQVPKGAMVSCIVSSANRDEDAFEDADTFNIDRRQKPSFGFGFGPHMCIGQFVAKTEINCAVNAILDLMPNIRLDPSKPAPEITGAQLRGPHSLPVVWD

>CYP226A16(Glaag_0358)Glaciecola_sp._4H-3-7+YE-5

MKTESIEDLQLETAYKAVSDTYRGTGIDIQKACKKQREEGGPVYKGDFVAQFGVPTNAGLQQGTRPTFTIFDYKDVMAVMRDSKTYTSGFIAEGLGAFFDGLIILAMDGDQHRQVRSLLQPAFMPEAVNKWRPEIEAVMRRDFLEPLAPKKKADIMEFGLFFPIRVMYALMGFPTDDPEKYKKYAAWALAMVGGNQIDPTKIEEARRQAGIAVKSLYDSILEVVQERRASNEYKDDLIGRLIVAEFEGRTLDDHEIVTFVRSLLPAAGETTTRTLSCVLTMLFNTPGLLDRVRDDRTLIPKLIDETVRYEPMGTFRTREAASDTEIQGVKIPKGSFVQSMIVSANRDDTVFENGHEFDIDRKMKPSFGFGFGPHMCIGQFVAKLEMNCALNAMFDLLPNLRLDPDYPAPAIEGAQLRGCSSIHLMWD

>CYP229A1(Pfl01_3472)Pseudomonas_fluorescens_Pf0-1

MDPIIAATHADPYPYYAELRAAGGLTFHHGLKLWVASSARAVCAVLAHPDCRVRPVQEPVPKAIVDGMAGKVFGLLMRMNDGEAQRCPRSAIEPPLGLIDREEVGALVSARLITNDSDGLYKAMFRGPVCVVASLLGFTPAQARVISELTADFAACLSPLSNDLQLAAAHRAAEQLRGYFIEMLADPNPFLADIRQRFVGNEEVLLANLIGLCSQTFEATAGLIGNALVALHRQPELRNASVDSLLAEVQRFDPSVQNTRRFMANSCEIDGVRLEAGDVILVLLASANRDPALNENPDRFRVDRPNRRSFTFGSGRHQCPGQTLAMTIASATLTEILARNIDPGRFTWHYRPSLNGRVPMFSEVQP

>CYP229A3(PputUW4_02968)Pseudomonas_sp._UW4

MDPIIAATHADPYPYYAQLRAEGGLAFHPGLNMWVASSARVVAAVLAHADCQVRPALEPVPRAIADGMAGQVFGQLMRMNEGERQHCPRSAIAPGLDLIDTREVESLVSARLISADAVGLHNAMFRGPVCVVAALLGFTPAQGRIISELTADFVACLSPLSTSAQLAAAHAAAEQLSGYCAELLADPDNHSRLLAGIRQRFTGGAPQTLIANLIGLFSQTFEATAGLIGNAVLALIQHPSLHSESTSIEDLLAEVQRFDPPVQNTRRFVAAPCEIDGTRLNAGDVILVLLASANRDPQLNDNPDTFLLDRPNRRSFTFGAGRHQCPGQSLALSIAGATVRQILAMKPELDRLTWHYRPSANGRIALFKDWPVA

>CYP229A4(PSEBR_a3386)Pseudomonas_brassicacearum_subsp._brassicacearum_NFM421

MDPITAATHSDPYPFYAALRAAGGLAFDPGLNLWIASSAEAVCAVLHHPDCHVRPAHEPVPKAIAEGPTGRVFGHLMRMNEGERQHCPRAAIAPRLQDINPRQVEALVRARFLREGAEGLHQAQFIGPASVVAALLGFSPTDCQRVSELTGDFVAGLSPLSQAPHLDAAHQASEQLTGLFQARIEAQDNPLLLGIGQGFEGADPNSKIANLIGLLSQTYEATAGLIGNALLALIGDPALRRTLREAPTQIGSLLAEIQRFDPPVQNTRRFIAAPCKILGTALNPGEVVLVLLASANRDPQLNPRPDTLLLDRPNRRSFSFGSGRHECPGQTLAMDIACATLAAILEREPPLDQLTWCYRPSVNGRIPLFCERSRTDRP

>CYP229A5(PCL1606_36860)Pseudomonas_chlororaphis_PCL1606

MDPITAVTHADPYPYYASLRARGGLAFDPGLGLWLASSAEAVAAVLAHPQCHVRPVHEPVPRAIADGAAGRVFARLMRMNEGERQRCPRAAVEPGLQQVHAEEIGQRVCALLPGLPSDIGARLHDCQFHLPVAVVAALLGFAAHQLPEIAELTRDFVACLSPASHLAQRDAAHRAAEHLTGHFAVLLDRAPISPLLQRIVDGFGAQGRDSLIANLIGLLSQTLEASAGLIGNSLCALLNDRQLLAELRAAPARIGDLLAEVQRHDPSVQNTWRFVAAPCSIAGVTLEPGAVVLVLLASANRDPQLNPHPDRLLLEREARRSFSFGNGRHQCPGQALALSIASAVIGALVRDPTLEQPIGWSYKSSLNGRIPLFTTLPESH

>CYP229A5(TO66_12285)Pseudomonas_sp._MRSN12121

MDPITAVTHADPYPYYASLRARGGLAFDPGLGLWLASSAEAVAAVLAHPQCHVRPVHEPVPRAIADGAAGRVFARLMRMNEGERQRCPRAAVEPGLQQVHAEEIGQRVCALLPGLPSDIGARLHDCQFHLPVAVVAGLLGFAAHQLPEMAELTRDFVACLSPASHLAQRDAAHRAAEHLIGHFAVLLDRAPISPLLQRIVDGFGAQGRDSLIANLIGLLSQTLEASAGLIGNSLCALLNDRQLLAELRAAPARIGDLLAEVQRHDPSVQNTRRFVAAPCSIAGVTLEPGAVVLVLLASANRDPQLNPHPDRLLLEREARRSFSFGNGRHQCPGQALALSIASAVIGALVRDPTLEQPIGWSYKSSLNGRIPLFTTLPESH

>CYP229A6(JM49_18480)Pseudomonas_chlororaphis_subsp._Aurantiaca

MDPITAVTHADPYPYYTSLRARSGLAFDPKLGMWLASSAAAVAAVLAHPECRVRPAHEPVPRAIADGAAGTVFGQLMRMNDGERQRCPRAAVEPGLQLVPAEEIQQRVAALLLGLPRDTAARLHDCQFRLPVAVVAGLLGFAAHQLPEIAGLTRDFVACLSPASNQAQREAAHRAAEALSRHFEVLLDQAPVSPLLQRIVNGFGRQEHDNLIANLIGLLSQTFEASAGLIGNSLCALLNDRQLLADLRATPNRIADLLAEVQRHDPPVQNTRRFVAAPCRIAGMTLEPGAVVLVLLASANRDPQLNPEPDRLLLEREDRRSFSFGSGRHQCPGQPLALSIASAVVGALLPQSTLDQPIAWTYKPSLNGRIPLFTLLPESH

>CYP229A6(EY04_11415)Pseudomonas_chlororaphis_PA23

MDPITAVTHADPYPYYASLRVRDGLAFDPRLGMWLASSAAAVAAVLAHPECRVRPAHEPVPRAIADGAAGTVFGQLMRMNDGERQRCPRAAVEPGLQQVYAEEIQQRVAALLLGLPRDTTARLHDCQFRLPVAVVAGLLGFAAHQLPEIAGLTRDFVACLSPASNQAQREAAHRAAEALSRHFEALLDQAPVSPLLQRIVNGFGRQGHDSLIGNLIGLLSQTFEASAGLIGNSLCALLNDRQLLADLRTTPNRIADLLAEVQRHDPPVQNTRRFVAAPCRIAGVTLEPGAVVLVLLASANRDPQLNPEPDRLLLEREDRRSFSFGSGRHQCPGQALALSIASAVVGALLPQSTLDQPIAWTYKPSLNGRIPLFTLLPESH

>CYP229D1(A7J50_1920)Pseudomonas_Antarctica

MTPLQAATHVDPYPYYAGLRRNPELMFDADLGLWIASRASTVEAVLAHPDCRVRPLNEPVPKSLAQGAAGQVFARLMRMNEGQAHRCPRAAVEPALAGVGAQPIADVVGQLIDSMGNLDTLMFSLPVSVVAALLGFQAQQLASVAGLTRDFVACLSPLSSEPQLLKADAGASRLRQMFSDLLEETALLAHLRSGDWQDADVLTANLIGLLSQTCEATAGLIGNSLVMLARRPDLVDRILHTPALALSLVEEVARYDSPVQNTRRFVAEDCTIGNRVLAAGDSILVLLACANRDVEANPNPDSFELERAHRRLFSFGSGRHQCPGQRLALSIASQAVSALLHRQPGLLAGAGRFSYWPSLNGRIPRFHAAALSQ

>CYP229D2(PflA506_2140)Pseudomonas_fluorescens_A506

MTPFEAATHADPYDYYARLRRQSELVFDADLGLWIASGASVVEAILEHPDCLVRPPHEPIPAAIAQGAAGDIFGRLMRMNEGVQHRCPRRVIEPALGSLGTENIAGIVARVVETLDDPVGNPNEWMFRLPVSVIATLIGFAPSQCQEVAQLTQDFVACLSPLSDEAQLSEAHCGAARLRQMFQALLNNDDKRSGLLDRLYGEYQASAWKDDDALIANLIGLLSQTCEATAGLIGNALIALQRNPDLFEGMHATPMCVAEWVAEVARHDSPVQNTRRFVAQRCTIGGSVLEAGDTVLLLLAAANRDPCANPDPDSFLINRTQRRTFSFGAGRHQCPGQPLALAIASEVISAFLHRRPDLFARAYRWDYLPSLNGRIPRFYRDEETQQ

>CYP229D3(AA957_05280)Pseudomonas_trivialis

MTPLEAATHADPYAYYARLRRNEELLFDAELGLWVASRAETVGAVLAHPDCRVRPPHEPVPPAIAQRAAGQVFARLMRMNEGAAHRCPRAIIEPALAALDTQQMAVVVKQLSSRMNTPDEWMFTLPVSVVAQLLGVPSEQLKTVADSTRDFVACLSPLSSAAQLQNADAGVSQLRQVFSGVLAETGLLALIRRGDWQDAEVLNANLIGLLSQTCEATAGLIGNTLVLLSRRPDLVEQIQRAPTLVTALVEEVARYDSPVQNTRRFVAGRCTIGRRVVEVGDTILVLLAAANRDPDANPDPDTFQLERAQRRVFSFGSGRHHCPGQTLALTIASHAILAVLQRQPSLFASAIAFSYWPSLNGRIPRFDSTVLQR

>CYP229D4(HZ99_01685)Pseudomonas_fluorescens_UK4

MTPFEAATHADPYVYYSGLRRQNGLLFDADLRLWIASSAKAVEVILGHPDCRVRPLNEPIPPAIAQGVAGMIFGRLMRMNEGPQHLCPRMAIEPALASVGTESIADIVSRVVETLDNPVENLDELMFTLPVSVIAALIGLPSSRLQEVARLTRDFVACLSPLSDEIQLGEANSSATRLSQLFSALLSNDDDRSRFLSHIRSGCEATAWRDHDALIANLIGLLSQTCEATAGLIGNTLVALHRNPDLIKDMQPTPMLVADLVEEVARYDSPVQNTRRFVAKRCTIGGSVLEAGDTVLVLLASANRDSYANPDPDSLLVKRTQRRTFSFGSGRHECPGQQLALTIASEAILAWLHRQPASSARLYRWIYLPSLNGRIPQFYRDRETQQ

>CYP229D5(PFLU_3256)Pseudomonas_fluorescens_SBW25

MTPFEAATHADPYGYYSNLRRKNGLLFDAELGLWIASSANAVEAILGHPDCRVRPLSEPIPPAIARGAAGVIFGRLMRMNEGPQHLCPRMAIEPALASVGKESLSEIVSRVVETLDDPVEDLDELMFTLPVSVIAALIGLPSGRLHEVAKLTRDFVACLSPLSDEIQLGEAHFGATRLNQLFSALLSDQEDSSRFMSHIRSACAANAWSEHDALIANLIGLLSQTCEATAGLIGNTLVALHRYPDLTEGMQPEPMLVADLVAEVARYDSPVQNTRRFVAKRCIIGDSVLEAGDTVLVLLASANRDPYANPHPDSLLLKRTQRRTFSFGSGRHECPGQQLALTIAAEAILAWLHRQPTSSVRPCQWGYLRSLNGRIPQFNRDRDIHRYL

>CYP229D6(H045_00255)Pseudomonas_poae

MIFGRLMRMNEGPQHLCPRMAIEPALASVETDSIADIVSRVVETLDNLVENLDELMFTLPVSVIAALIGLPSSRLQEVARLTRNFVACLSPLSDETQLDQANSGATRLSQLFSALLSNDDESSRFLSHIWSGCEATGWTDHDALIANLIGLLSQTCEATAGLIGNTLVALHRNPDLIKDMQPTPMLVADLVEEVARYDSPVQNTRRFVAKRCTIGSSVLEAGDTVLVLLASANRDPYANPDPDSLLVKRTQRRIFSFGSGRHECPGQRLALTIASEAILAWLQRQPASSAHPYRWSYLPSLNGRIPQFYRDQETQQ

>CYP229D6(VO64_5352) Pseudomonas_fluorescens_LBUM223

MTPFEAATHVDPYVYYSRLRQQRELLFDADLGVWIASGAGVVEAILEHPDCLVRPLHEPIPAALAQGAAGHIFGRLMRMNEGAQHQCPRRVIEPILASMGTQNIAGLVAQVVETFEISGDNPNEWMFTLPVSVIATLIGFTSNHLQEVAQMTRDFVACLSPLSDEAQLSKAHCGAARLSQMLQALLSNDDKGNCVLGRLYSDYDTSAWEDDDALIANLIGLLSQTCEATAGLIGNTLIALQRNPELFEGMQATPTLAAEWVAEVARHDSPVQNTRRFVAKRCAIGDSVLEAGDTILLLLAAANRDPCANPEPDSFLLKRAQRRTFSFGSGRHKCPGQPLALAIASEVVFAFLHRQPTSPAPAYRWRYLPSLDGRIPQFCRDGEIQQ

>CYP229E1(POS17_2517)Pseudomonas_sp._Os17

MDPISAATHFDPYDFYYARLRARGGLTYDSALGLWVASSAAAVAAVLNHPACRVRPLQEPVPRAIAGRPAGQVFARLMRMNDGPRHGCPRQAMAPGLQALGGIDLMPWLARWRPALRAPQCAADLQRWQWCLPVALLAALLGVPAAQCEELARRTGEFVACFSPLSTEPQLQAADSAAQALLRQMQAVLDNPQPGPLLRTILERSNGLAPEDLQANLVGLLAQTHDACAGLLGNSLLALLADPALARRLQHDPRQLQDWLLQLQRLDPPVQNTRRFVAEPCTLHGVDLQAGDSVLVLLAAANHDPALCAITGSDPARQGFSFGAGAHRCPGRDLALNIVHSLLRTLLQPPGLDALALQWQYRASVNGRLPQFSDAPGSRPMDWQPADA

>CYP229E2(PFL_2514)Pseudomonas_protegens_Pf-5

MDAISAATHSDPYFYYTRLRARGGLTYDCALRLWVASSAAAVAAVLDHPACQVRPSTEPVPQAIAGRPAGQLFGRLMRMNDGPRQRCPRAAIEPALQGLCTAGLETHLARWQPALAGPACAADLQRWQFVLPVALLASLLGVPAEQCETLARRTGEFVACFSPLSDQPRLQAADRAALELDAQMQALVQAPQHSPLLRQILDRSGELAPADLRANLAGLLAQTHDACAGLVGNSLVALLAAPAMQQRLRQEPGLLGDWLLERQRLDPPVQNTRRFVTAPCTVHGVELQAGETILVLLAAANQDPALAAITGSNPAHQGFSFGAGAHRCPGRELALGIVRCLLHALLQGPGLDALALDWQYQASVNGRIPLFSDRGEAEQ

>CYP229E2(PFLCHA0_c25790)Pseudomonas_protegens_CHA0

MDAISAATHSDPYFYYTRLRARGGLTYDCALRLWVASSAAAVAAVLDHPACQVRPSTEPIPQAIAGRPAGQLFGRLMRMNDGPRQRCPRAAIEPALQGLCTAGLETHLARWQPALVAPACAGDLQRWQFVLPVALLASLLGVPAGQCETLARRTGEFVACFSPLSDQPRLQAADRAALELDAQMQALVQAPQHSPLLRQILDRSGELAPADLRANLAGLLAQTHDACAGLVGNSLVALLAAPAMQQRLRQEPGLLGDWLLERQRLDPPVQNTRRFVTAPCTVHGVELQAGETILVLLAAANQDPALAAITGSNPAHQGFSFGAGTHRCPGRELALGIVQCLLHALLQGPGLDALALDWQYQASVNGRIPLFSDRGEAEQ

>CYP229E3(PPC_2556) Pseudomonas_protegens_Cab57

MDAISAATHSDPYFYYTRLRARGGLTYDCALRLWVASSAAAVAAVLDHPACQVRPSTEPIPQAIAGRPAGQLFGRLMRMNDGPRQRCPRAAIEPALQGLCTAGLETHLARWQPALVAPACAGDLQRWQFVLPVALLASLLGVPAGQCETLARRTGEFVACFSPLSDQPRLQAADRAALELDAQMQALVQAPQHSPLLRQILDRSGELAPADLRANLAGLLAQTHDACAGLVGNSLVALLAAPAMRQRLRQEPGLLGDWLLERQRLDPPVQNTRRFVTAPCTVHGVELQAGETILVLLAAANQDPALAAITGSNPAHQGFSFGAGTHRCPGRELALGIVQCLLHALLQGPGLDALALDWQYQASVNGRIPLFSDRGEAEQ

>CYP236A17(Patl_2305)Pseudoalteromonas_atlantica

MKNYQSSDPFKEARVSSGCAHMNDQDDPVTMILRLKDVRKTAHNWKTFQSGAAPGRIVIPSEVNIRDTRQIPFEVDPPLHGAFRSLLDPWFKRPLKAQYQETLAQQVNALVDDVLKADSIDVVEDFALCLQSRALTLLLNTDADEADTWIGWGTHVFRSEDDPLDGDKAAILYDYIDEKIKASSPNPEGDLYSVLLAAEVEGKKLTHEEIKGIMILTFAGGRDTVINAVTNTVSYFAQHPESLARLRNEPEIIGRAIEELIRYFTPLTQMGRVVTQDTQVCEHAIKADSRVSLCWASANRDESVFESPNDVILDRKLNPHVSFGFSHHNCLGATHARQIMRVLIETLMAKVGSIDIIDFEENIEYLGEFERKVGFHKLHVNIHPR

>CYP236A18(Glaag_2453)Glaciecola_sp._4H-3-7+YE-5

MKKYQSPDPFTGAREVSGCAHMNDQDDPVTMILRLKDVRKTAHNWKTFQSGAAPGRIVIPSEVNIRDTRQIPFEVDPPMHGSFRALLDPWFKRPLGDEYRAQLGQQVNALVDEVLTKGVIDAVEAFSLCLQSRALTLLLNTDASEADTWIGWGTHVFRSEDDPLDKDKAGLLYDYIDEKINAASADPEGDLYSVLLAAEVEGKKLTHEEIKGIMILTFAGGRDTVINAVTNTISYFAEHPESLTRLRNEPDIIGRAIEELIRYFAPLTQMGRVATQDSTVCEHAIKADSRVSLCWASANRDASVFESPEEVILDRKLNPHVSFGFSHHNCLGATHARQIMRVLIETLIEKVGSIDIVDFEENIEQLGEFERKVGFHRLNVHFHPR

>CYP236A19(AOR13_2915)Alteromonas_stellipolaris_LMG_21856

MQTKNSDGGCGTPSHQQFEGEKSKCPMSEHVMGNVSQLDDPFTALRQGDGVLDIDDQGDPVKMVLGLKDVRKAAHSWQLFQSGAIPGRIVVPSEVAIRDVRQLPFELDPPAHKGFRGLLEPWFKRPAGKAYQDKLTDIVSIMFDSILDNHTAINKRSSPEMVTHDAVKDIALVLQSRALTVLINVPMTEAQTWINWGTHVFRSDDNPLDASKANVLYDYLDEQIAHAKKAPGEDIYTELLHAEVEGKALTEEEIKGIMILTFAGGRDTVINALTNSLAYFSENPDALNFINEHPENLNSAIEELLRYFSPLTHMGRVATKDTQVAGHDVAYDSRISLCWASANRDESVFESPNEVNLTRKANPHVAFGFGIHNCLGATHARALLRIWVGQLAERVARVEVVEADENTEQWGEVARKVGFHKLLVRLHKRA

>CYP238A1(PP_1955)Pseudomonas_putida_KT2440

MEILDRPQAPSDFNPMSEQSFRDPASICQRAREETPVFFYAPLGVWMVTRREDAERVLSEWETFSSLANSPNVPEEFRSRFAPSVMADSIVAIDPPRHTQARNVIQRGFMKPKIDPLEPIIEQRAHEIIDRFAGESGTEIMNNYCLELTTRTLMALYDLPLEDRPMFERIRDVSIKVLASVYEPMQEPEKSRVWNEYVSGYEYFYQLVEQRRNSDARDIISTMASQKDNQGNPALSTERIALHLVEIAFAGTDTTAQMMANAILFLDSHPEALAAAKADKTLWSRVFEETVRRRPSAPFAGRITTTEVEIQGVKIPAGSPVWVSLAAANTDPRHVGCPMNFDINREAPQDHLAFTKGRHTCPGAPLARLQGATGLRVLFERLPELKVVPDQPLNFAPMALLPVRLSLQVIW

>CYP261D1(MVIS_3494)Moritella_viscosa

MNANMVKDMNASTISLDELPGPKQTPVLGNFTQISSESFHTNLESWAREYGSAYQIKLLNKQFLVISDPKIGLEIIKQRPKLFNRTERLEWLFEDLGFHGVFSSNGDKWKRQRRLIMPAFSYKTLANFVPHLKSLSINLQTTIDQKIATGAAFNVHKLLQYFTIDITTSLVFGYQTNMLSGSTDTHLRDNIDRLFHALNKRSKYPFPWWHYIRTPETRRIDKAREEVYQLAVTMITKAKADLAGNSELSEEPDTILQAMIVASDSEDNKLTDDELVANILTLLLAGEDTTSNMLAWTLYYLAKNPHLQQQVIDEVSQVCNGDIETLDLVALEQFEFIEAILREGLRLKGTAPLISAEPTEDTVLSNGIQLPKGTAIFILTRPGGLDESVVKCPEQFNPERWLATPEKPVCPHLQSSHIPFGAGARHCPGERLAIMEGKAVIARLCWYYAINQPEQAAEVGEEFAFTMRPTNLHLTLTPR

>CYP289A10(GU3_03105)Oceanimonas_sp._GK1

MSSKLHQWDPAGEQDLIEACDRLRPRCPLAHHDALHWSVLRQQEARQVLSDHATFSNAVSRHLSVPNGMDPPEHGPYRRLIAPYFSPERMAEFEPRCRAIIEALATGLRGELELVQAFAEPLSLQLQCAFMGWPETLHGPLRDWTRQQHQATRSGDHAALQALANTFDGYIREQLEGRRCAQAPDDITTRLLAERVNDRPLTDEELISLIRNWTVGELGTITACVAILAHYLAAHQEVQELLRQTPEVRGAAIDEILRIHPPLLSNRRITTQDVTLGGQSLPAGSRLSILWASVNRDEGVFGDPDEFRLDRNPEHNLLYGAGIHRCPGAPLARMELELALDAMLSAGRLTLVPGKPPVKARYPTGGFSELWLRLE

>CYP289A11(ACG33_09565)Steroidobacter_denitrificans

METDWDPRSAEVLRDQRAAYDSMRERCPVAFSEFMHWSLFRHEDIERALLDHETFSNAVSQHLTVPNGMDPPEHTAYRAIIDRCFTPERVDAFAPVCRGIAADLVRNMLGRDEVELIADLALPFSVYVQCAHLNWPTTLHEPLIRWTRRNYEANLKRDRHITSEIAREFEALIDDMIEARSQGQSKPEDDLTAVLLNERVWGRPLSNEEIASILRNWTVGEIGSISAAVGIVAYYLAQHVDLQQQLRTQPSLLDAAIDEILRMFGPLVANRRITTRPVEIGGRKIGAGERISLMWIAGNRDGRVFDDPDSFRLDRDPAKNLLYGAGVHVCPGAPLARLELRMITEELLRQTTAIEPVPGKTPVNAIFPSSGYATLPLCIKR

>CYP289A12(Q7A_103)Methylophaga_nitratireducenticrescens

MANNSRKADWNPRSEEALKDQISTYDTMRTQCPVAWSDYQHWTLFRHGDVMRVLEDHHSFSNAASSHLSVPNGMDPPEHALYRQVIEPYFSTQAMSEFEPTCRNIARQLVDTLPSGQPFDVVDLFSRAFALQIQCAFMGWPDSLRAPLREWVLKNHHATLSGDRQAMADVAHEFDGYIHDLLETRRQAGANAPKDVTTSLMKETVNGQTMTDEALTSLLRNWTVGELATISASVSILLNYLSEHPDLMHSLSIDTNALSDAIDEILRMDAPLMSNRRVTTRDVEMGGKSIPAGEKITILWASANRDEEVFGDPDAFSPQQNREQNLLYGAGIHVCPGAPLARMELRVLMEEFLAAVDTLKHAPNEQAERAVFPTGGFSYLPMIIEKN

>CYP289A6(PSF113_3435)Pseudomonas_fluorescens_F113

MDEKSLPDWDPRSEAVLKDQTTAYDDMRRRCPVAYSQYGYTSLFRHEDVLRVLKDHESFSNAVSRFPSVPNGMDPPEHTVYRNLIEPYFSPEHMDAFAPVCRDIAVRLVRALPDEGPSELIGEFAQIFALRIQCAWLGWPADLHEPLRLWTLKNHAATLARDDADLAAVALEFDGYIKDLLEVRRTAGAAAPNDITTILLRDQSLGRTLTDDEIVSILRNWTVGELGTISASVGILAHYLAINSEWQQQLREQPALLPAAIDEILRIHAPLIMNRRVTTKQVMLGGRTQAKGARIALNWASANRDEAVFGDPDEMRLDRDPELNLLYGAGIHVCPGAPLARLELRIVMEELLGRTSRIALAMDKEPVNAFFPASGFSSLPLRIQKPSSQALGACLPPEIP

>CYP289A8(PSJM300_12960)Pseudomonas_stutzeri_DSM_10701

MSEEPKSDWDPRSADALADQIAAYDALRARCPVAYSDYLQWSLLRHADVMQVLLDHETFSSAVSSYPSVPNGMDPPEHGLFRRLIEPYFAPPRMQAFEPLCRAIASELAAALPKAGDVELIDGFAQDFALRIQCAFMGWPGDLHEPLRQWTRKNHRATLAGAHAAKAEVALEFDGYIRGILEARRAEDHLAGDDPTDRLLAERIEGRPLSDEEIVSILRNWTVGELGTIAASVGIVTHYLAARPELQRQLRQDLSLLPAAIDEILRIDAPLIANRRITTRAVELGGRRIEAGERLTLLWASANRDEDVFDDPDAFRLDRDPSLNLLYGAGIHVCPGAPLARLELRVVMEELLRQTLHIALVPDQPPVRACYPAGGFSELPLRIG

>CYP289A9(Maqu_1895)Marinobacter_hydrocarbonoclasticus_VT8

MAEQHHEDWDPRSAEVQKDQIRAYDAMRKECPVAWSDYQQWTLFRHADVMRALEDHHTFSNAVSAHLSVPNGMDPPEHTPYRKAIEPYFAPEPMARFEPVCRDVARALVQTLDKNKPLDVVNALSRPFALQIQCAFMGWPDSLHQPLAEWVMKNHRATLARDHAAMADVAEEFDGYIRDLLDSRRQPNKPAPDDVTTRLMREQINGQPMTDAELVSLLRNWTVGELATISASISILTNYLAHHHELLNNLKAAPEQLPEAIDEILRMDAPLISNRRVTTREVEIGGRTIPASEKITLLWASANRDEVVFGNPDQFCPRQNAARNLLYGAGIHVCPGAPLARMELRIFMEELLKQIEAIEQADGEQPERAMFPTGGFNYLPLVFR

>CYP289D1(HDN1F_30550)Gamma_proteobacterium_HdN1

MSNSSNQDWAPQSDSVQGDQRAAYDQMREDCPVAYSEYAHWSVFRHKDVLRVLLDHETFSSHVSRFASVPNGMDPPQHTRYRELINPYFSAEKVAEFEPLCEKIADSLAQSVLCGEQVELMEAFAQPFALQVQCAFMGWPLSMQGTLLSWVQRNNQAIFKQDRALLAELAAEFEAIIAGLIAERRQAKVGPDCDVTGALMHEEIDGRLLNNAEIASILRNWTVGEVGTIAASVGIIAHFFATNPEWQARLRESPDLLWKANDEILRIHGPLVGNRRRNTCPVEIGGRQIPAGERISVNWIAANRDPQVFPQPDQFSLERNPADNLLYGAGVHVCPGAPLSRMELVVVARALLRSTDAIRLLEGQPPVLAGFPASGYARLPLALG

>CYP1043C1(YC6258_04794)Gynuella_sunshinyii

MNIFLLTVTVAFVLSLPYWLPPLIIRLRMNVFTRINGEEALHLPSDSFNAEDFKTLYGNPALSGRSKGAELSDLFWYWLAPGPEVHPEHLELSDRYRNLSRFTRQLMARSRTELESMIDQYQQDPLRLSLHHKKNWTSIRLRDAFMPLWADFFYRLLFNEPCNEKTRNLIVNHASDVVNALKCCKLRNMSKRHQLTEFLVNKLESNEFPHPFPPGLTTLEQAHYLQGAFFNTAIVQMSEAMSHLIMVIAQHPHCQERLRSGDHDHYLDDVINESLRLNPLFGIAHRIVTDTVNFKGSTIKKGTVVCFNYPEFHKQGYENPDQFNPDRWQQCPAKDSNFMPFGITSNRSCPAQGLATVTMRRLAFHVINSYWFTSPVPHTRSLPNRGPCLVMLSQSVSGHRFIRHIILPLMLLRDHWEDLYRSLTQLVFGTIMILHAKKLKLCKKYFQQLESL

>CYP1097B1(OLEAN_C17420)Oleispira_Antarctica

MTIKSATSSSATPPLRQFKDLPGPRPWPLVGNALQIKLSRMHQDIEDWAQQYGPIFKMHLGPTKVLVIADHQIETALLKDRPDSFRRPKQLVDTLEEMGLQSGLLTAEGQTWKDQRRMVMASLSANNVRSYFPSLLKVTKRLQGRWLNAVDTDTTIDLQADFMRFTVDAIAGLSFGSDINTLETDDDEIQRHLDKILPTLFKRVNALVPYWRIIKSPADRQLDRSVALVNESINGFIEKARARLDSDSARREQPQNLLESMLVAADKSDSGVNNNDVAGNVITMLLAGEETTATTLSWLIYLLKRNPEALKCAQQEVQSILAELDDDLDSLTPEKLNELKFIEACTLETLRLKPTGPFDVLVALEDTEVAGIAVPKGMWIWVVCRHDTLAEHYFPDPKAFKPQRWLKTEDNDLAQSRRVSMPFGSGPRICPGRYLAMLEIKLAMVMLLKNFDIESVDTPDGGEAKEIMSITMNPVGLTMKLQKRL

>CYP1104B1(FF32_08560)Halomonas_campaniensis

MHQDSVLCKAVTTKSANDHQGLSAALNRAKGLEPPVKVTPVSASKGIVHRLRLAQHNLLSVWREEDYSIRTSRMALMKQDYVLCNSPDTVRRVFLEQHDNYDRKSPQMRRALEPLLDDGLFVSDGDLWRQRRRQCAPSLTNALLPGFCTTMTASAEETAKRWAQHPSDTPIDMLTEMAHLTARIIGRTIFGDDTTDEEAEQVVAGFTEYQRHSEHLDMANMLGLPFLSLLGNPLRRARTRYSAKQVHEIIDRIIDRHTHRSGQGQHSLIESFMTSMKDGGDNSCPMGRKAVRNEAIVMFMAGHETTANALAWCWYLLDYDPQAMARLQHELDDVLGGRTPCYEDVAKLPYTMAVFEEAMRLYPPVPMLSRQARGGDKVRNRDVREGTVLLVSPWLLHRHRKLWEAPDHFVPERFLPDAPRPDKFAYLPFSVGPRVCLGKRFGLYEGVLCLATLAQHFTPKLVKGHQVSIECRLTLRPQGGLPMMLQPR

>CYP1104B2(A9404_09670)Halothiobacillus_sp._LS2

MTEFARLTLPEGCRDLAEPDPVKVIPPTAWLDSTRTLSELKTNLLSIWPERAYRGLTFAFQLLNQHYLVCNSPDTVKRVFLEEHDNYDRKSPQMRHALEPLLGDGLFVSDGALWKERRDYCAPAFEAERLPDFAGVMVESAQEMADRWARLPHDQPVDMLNEMARLTSLIIGRTIFGDDTSDAEAAQVVDGFSTYQKAIEQMNWADTFGLPYLRWLSNPLSRFRAQRSAARIHEVIDRIIERHKARKDSDRVTLLSMLLEGHPGSRGQKRCPLHALGARNEAIVMFMAGHETTANSLAWAWYLLDHSPRAADRLHEELDRVLGGRAPTLADVPKLPYTRAVFEETMRLYPPVPVLSRQARAGDEIRGKALRKNSIILVIPWLLHRHELFWEKPNQFIPERFLPGQPRPDKFVYLPFSVGHRVCLGMRFGLTEGILCLATLAQRFRARMAPEHSVDIECRLTLRPRGGLPMYLEPRSS

>CYP1104B3(Hneap_1482)Halothiobacillus_neapolitanus

MPESTFERLPEAYRDLNEPPPVAVAPPDTWLKDWSAILKLRKNLLVLWPKRAYEGKTFTAQLFKQHYFICNSPDSVRRVFLDEHDNYDQKSPQMRHSLEPLLGDGLFVSDGAVWKERRAYCAPAFESELLPDFAAIMVDSARELADHWESLPAGSSIDMLNEMARLTSRIIGRTIFGDDTSEAEAATVVDNFSQYQKAIEQLNFSDSFGLPHLKWLGNPMAKWQSLRAAQKIHTVIDQIIERHPQRAKPESPTLLSYLLGEHTSKKTSGKRCPLSSVDARNEAIVMFMAGHETTANSLAWVWYLLDRYPRVAEKLQEELTQMLGDRSPRFEDVPQLPYTRAIFEETLRLYPPVPVLSRQARASDEIRGKAVPPNSIILVIPWLLHRHNLYWEKPNHFIPERFMPGQPRPDKFVYIPFSVGPRVCLGLRFGLTEGILCLATLAQRFRAKLKVGHEVEIECRLTLRPQDGLPMQLEPTASSVTPPQ

>CYP1138A3(R615_00540)Thalassolituus_oleivorans_R6-15

MDFLAEYDAAPDVEKYPLVRKWIKTNPLPFFKQLRAERPILVTPECTLLARSSDVRDCLQMPTIFSVDLYKSKMGVTDDNPGYLMAHDDDALHYREKSIMQSMLNRDDIPRIKKLISDTSKKILDDADGQIEIVNNYCRMVPVILVQKYFGFDGIEPEALFKWSYWNQYNTFYNQPFDLNPKDKHDHIVQCHEECTAELTEYLKAMILRKLFAVNVKDRVLKPALKIKNAVRGLIGKEPDVHEDDIVTRMLRSSFPDEVDFPLIRLGRNVGGLLIGTVETTSKAAAQVIHFFLERPELLAQAKAASLLEDTDEIESLVWEALRFVPIGAYMFRQVAQDYIIAEGTEHETSLKKGTTVLALTQSAMFDECAYKNADEFNKDRNWYNNFTYGFGAHDCLGKYIGMAMLPEMVRQVLALDGVHAVSAMDFKDGPFPEHYELVWNK

>CYP1142A1(VIBNI_A1030)Vibrio_nigripulchritudo

MGCLKTSVNRMKRRLSPFTRQLRLLAEIMSITTRLKEWASNNVPLVFSILRFIQPNVIYKNNAVITRFKDVQEALSRPNELGVTYAEKMRIITDGKNFFLGMDDIPDYTRDVSNMRLVARRDDLEKIVNPMTEKMARVIMEKSSGRIELVSELTSVVPSQFTAEYLGVSGLEPKTLFDWTCNLFQYLFYPDCPKEVEDAAIRDAAGLRAHIDELIKQRKQQPEKDDVLGRCLKLQESGTPGMTDIDIRNNLIGIIIGLVPTTSKCVIQVLDYLLDRPELYKQAQRAAHLDDMHTLQQFVLETLRFTPFGAGLLRTANCDYTIAKGTWRAKKIKKGSQVFVATQSAMMDGRDVSSPKSFKLDRPRHIYMHFGYGMHTCFGQYINLIQIPNILKAILKCEHVQRAAGKEGQVTYRQPYPISLTVTHEVMTRPAVKTKQESKEAEPSKVA

>CYP1157C1(PsycPRwf_1012)Psychrobacter_sp._PRwf-1

MFGNSKIKALYSEDDQRSLTDKRRQQQDIIKNEAGEWVLLRHADVKAAALDDATFSSHVSRFLQIPNGLDGAEHDQYRALINRYLSQDAITPYVPLFQHVATELVSGLPKGEVINAVTDIGTVFAVRAQCQWLGWPAEVEPILVQWVNDNHQASRLKDHTKLATVAQDFNEIIRSVIAPFRDTDSAAASHNKANQDSITAQLCREQIKGRPLSEAEIVSILRNWTGGDLGSMALCVGVVVAYLAHHPKQLQHCANASDAELEAIIDEILRLDNPFPSNRRITKCPVTLGEHQLPEGAKVHLDWISANRDEAVFGKDTFDPVRHAADNLVYGIGRHVCPGRLLATWQLRILIRALLAHVEAITPAPNESFERQLPPLGGYSRVPVVLS

>CYP1164A1(HDN1F_07780)Gamma_proteobacterium_HdN1

MLTVDLYHLRRRLLKGSITQGLQGYAKVRALLPEQRVSQSTHRAPPTHPTTPEDFRPLETGCFQNPYAFYRMLRDEYPVYRLRNGIYCISRYDDIQTVSRDTNTFSSEHQGVIANLKPGQDLLQQVRRFEKMTALGIIPADVLATSDPPQHTVERKIGHTSLNAHFVKSLEPEVEKLSTNMLAPLLDAGEMEFMQAFGWRLPMVLIIRLLGLPEQDFTKIKKWCVDTLNSQNGIQTSAELAQSYVSAINFLDYCWTQYLKVKTNPGNNLMGILAKSADDAATPFDDKKAVSAIFQLLIAGSDSSATTMGNALKLLIERLDLQEKMRADPALVNDFIEEVFRLESAFQGHFRWVTKDTALHGVQLPRGSRIFLMWAAGNRDERFFENPDELILGRKNGKKHLTFGHGLHACLGRELARMEIRIVLQQFLTHTRNLHITGETPFVASMFARTLVTLPIRFERATKPAALG

>CYP1165A1(HDN1F_19730)Gamma_proteobacterium_HdN1

MTAKAKLPPAPKDPKYRQALGLGIGIWDYMEKNQRELGDTFTLTLPGQGPMVWTSNQDMIKDILKLQEEQVDASLVQLPMDLGEHNTVFLNEKEHQDSRKIVIPAFNTTRLRARAPAMHDIVTEHINSWKVGQKLNVPRMIGDITLDVICYTLFNLRHGERKDRYQYLMGNWMLEATSDTMFTFGAIFGAKNFRQWLNKKYLARADKNQGGNGKKGILPWKRAVDLKVQLADMIREDIRDIRRRMDESETHLLSVIARAKDDKGNLLSEERVISETIGLLIGGHETSAATASWFAIWLQQRPDVNDKIRREVLESIQNEGKFDPVKVSELPYLTACLNESQRLTPSAVGFIRWLRQDTQLGSHLIPAGSSVLPCIYLTHRDPAIFGPDVLEYRPERWMEGKKYLPSEFLPFGGGRRACVGLNQARQQLRIAFAEFARRVEFTSEYQHSNKMPRSRMIGGQTEPEKGVWLTVKQIRPESYGMPDTVGAAETATA

>CYP1165B1(HDN1F_04700)Gamma_proteobacterium_HdN1

MLSKLHANASQLPPGPSGLKFVNAMKMGMGAFDYLWENFSKYGDLFTLQFPGMTPFVWMNRPDLVQKIFNLKPEQIDASKLPIPIDIGVRMTGFLNGAEHMLSRKIVVPPLIAGRLQARAGIMHEIISEHINHLQVGEEFNTPRKIGDITMDIAIYTLMGLRGGARAAAYKEVMLNWVGAATNNTMFTIGTLYGPYRWREYLNEKYLEEIAEGSTGTGKHRLLPWMKSVDYKVELGNMFREDIRRTRAENDSKRTDMFSVMCRATYEDGTLLDEERIISEAMGILVGGHETSAATSAWHMLWMLKRPDVYKKCREEVLACIQQHGGFNPLAICELPYCNAVLNESMRLTPSAVGTLRYLTCDLEVDGYTLPAGTNVLAGAYVIHRRKDIWGADAEEFRPERWLEEGRFKPGPFEFFPFGGGRRACVGSNHAKQQLRILWAEFYRRVSFSSRYSNNDVWPGQQQVSGQTEPTGGVPVRVTRILPANTGYPEIQTEAAAPKEVANA

>CYP1172A1(LHA_1423)Legionella_hackeliae

MSNGNKANPVTITVNRRGFFGNLVETIKQPVIKIVDYAKGTGNLYRLLVNYGAAKDPNAFHKMLDNDYSQIPPTQSKVVIEPILSPSSSQIYDRVVGIANPVILQELRKIPRVETTRPSYYEEHPDVPRISGGRAFSSVLDAIGMGILSAESDVHKLFLQEMTAALTIRLNNKSDSLYAGNDYKTRFINVIREETQELISTIRAAAESKQKEPCHLDFRFYALKIFLRGFYPEATWENDWINKLSQEIEIVSDMAFKGMVNPYTDIEALRKEANKRMDPFIERIMKEKQGYLRANYVNEATPEILRQIIVSLLFAGGDNIKKYLDHIFVEFGNDKIREKYLQKKLAGEELKTYITEIGRLYTTIYAQPGDALDDFVIEYKGEKIYIKAGDKLHYTTWRANRDEQEWGPFANEFNPEENKKYYDQLNPLATFGSGARACFGKTITMSIIEYLMNEVLTICRWKTFVNGQENSHPTEFNFNNGVQGTIGLIFTLFQEPVLRSTENQCDSNVLDQVPNWRTTAANTDGVPSKCGSFKQPVKRCDNPVEEPATKLEL

>CYP1199A3(SB85_18120)Xanthomonas_sacchari

MSATMVDRDARATVFAPRLQALLRAHVGQDAFRLDAETVGVAGAALSDRLLAARPATEHERPTFKPLHGRSIARSEAAKLMQAIGRDVREALKRPPPPTLDLSGPWPHIGHVYLRDLLLGGDPWRLRLLMDRTLELTPRLTWAVIAAGALAPLAPGAEASALATLLSAADGYRARRDAMGLYRRTAAPVCFTISTLVANALWLGSPFDARVCNRNILYETLRLLPPSWNILRNASPEYGALDPRIGAADDVLVLPLLSHRDPALWEEPEAFRPERWDGVDPDALPGYLPFGHASERCWGRHMVMPLAERLLDLLRGQDLVADPQQRRATVPLTGLLGVAQVDVIRHTRARGVACRTR

>CYP1199A5(FD63_14695)Xanthomonas_translucens

MSAIVRDRQARATVLAPRLQALLHDHLGQDAFRLDADTIGVAGPALTHRLLDARPATEWERPTFKPLHGRSIARADASKLMQAIGRDVREALKQPAPPNADLSGPWPHVGHVYLRDLLLGDDPLRLRLLMDRVLELTPKLTWAVIAAGAVAPLAPQAGASALATLSAAAGGYHERRYAMGLYRRTAAPVCFTISTLVANALWLGSPFEACTSNRNILYESMRLLPPSWNILRNASPEYMALDARIGAADDVLVLPLLSHRDPALWDEPQAFRPERWDGLDPDVQPGYLPFGHASERCWGRHMVMPLAERLLDLLRGQGLAASPRQTRAEVPLAGLLGVAQVDVVRH

>CYP1201A1(LMI_0196) Tatlockia_micdadei

MIILLLILGALVVIRVYQQSQRALSLETTSPATTDYLKLVKDVVSLRQSQSEENKMQLQTRFMSFVADLGARSLQEGGSGVGYFRLPNLTPVYVLSNRAVIKKFYEGNAYLDNEQKKQIRFGQKKFFTRLAIILGQDNLMSADLGSATHSEVRAAILSRNEMFRPKIADLVLRYFKEYEKSEQGRPLSDVMDALSRQVLIATYFDPLVINQFETLYKPELTKELISFLFSLDPISTNEQQSLVKLREKIFELGCNLIFSTSEIKQQLLEEKSWLNYLLKIRVLGNEALQDELARLDIFVSPKRELSSSQCERLVRYAISNNDRTPLAAAVKDAVNESLFIPLLGFDATATALITSLRIIIQDRRIYTLVMKEIREKLANQEDFVLHSPWDLGKEGALSYMEAVILEALRLSPPAPMIPETINETLSLGIDGKTLVLPKGALVFIPLESLHVHPSYFPDIPLSPRGQEILGKQSMSASDIFPERWLPKHKDEIYNADFFQEGYLNETDSSVNPRQLEKEGGLLTFKTGPRRCPGLRIALAEILALFKMLSVFKFELDNEENLALGFHYATPLQRNGGKGTITITPLEETKQTATVRNSAAQESSFSASGNSPFFKSSPESRRSRLDAICLSSEKTAMTGRI

>CYP1201B1(LLO_3439) Legionella_longbeachae

MKEQDDQLDYIIMSEDPLPLFIWDSIMDTLIEKVKNISSYISNIWQGSSQGIEAKKEKNTAGTLTYPSLGHYLTFLKDITLLKTDPAKKAQLQSYFMTFVTELGAHSLDEGSGVACFSLPDLTKVFVLSNQQAIKQLYEKSNEKKFGQKPLFQRLALILGPDNLMSSALGSEVHSKIRASILSRNESNRRNVAEIVAEFFKEYESEQMVQRQSLSELMDRLSRRVLIATYFGKEVVQPFEQLYSSSITKELIDCLFSLDPIKNSEEKELLALRGKVLDLGYNLIFSLEAISKQLMSEQSWLNYLLKVRVLSNPDAASELQKMGINSTTNLTAEQCEFLIKYSQLHIDNTLLSTLIRDVINESLFIPLLGFDATATALIASLKIALQDKRIHTIIKQEVKQKILAGEAFELHSPWDVVKKENALSYTEAVLLEALRLSPPAPVVPEIISEAITLNIDDFSFTLPAGSLVFIPMQNLHTHEKHFPDIVLSEEGQKVIGKKLITANDIFPERWGPKQKNNELYNANFFSEDTDTYKPGVLQKEGRFLTFKTGARRCPGLRIALTELLSIFRILATYKIELTGEEDLQLSFHYETPLQRNGGMGLMKITPKNTLTTMSTVSSKEEQSALSRSGFHFFSQAQIPETQDLSTREPLSENSSFLKMTVHV

>CYP1202A1(THII_0230)Thioploca_ingrica

MWFNRSYFRQRQVPGPRRFKLLGHLLAGRRDLLTTLQQCQRDYGDLLRFSLGPKTVYVACHPDMAEQILIRDLETFGSLSQQAKPVGLALILGNGLLQSYGEDWKRQRQMLQPLFHKRPVERMAIHMTTAGEQLLQRWHSTYSPGEVINVLEEMVKVTLDIICRTLFSVDVITPLDTLQTVLPILIEHAATSLKNPLLPPLNWPTPRNRRFKQALQTLDDIIYRLIQQRQMSGEQNDDLLELLLQTRDEISGETMNRQQVRDQVATLLGVGHETTAAAMTWLWYALDQHPLVLQRVQDELATVLAERLPTLEDLPQLTYTRQVVDEVLRHYTPAPLVARLVLRDTEINGYPLPAGSTVFVSLYNIHHHPDFWINPEQFWPERFATREESVKHRYAYLPFGVGPRFCLGNHFALLEMSLLLAQIAQRYTLKLVPNYLIERDVTMTMKPRHGLLMRLYPR

>CYP1225A9(AYM39_01420)Methylomonas_sp._DH-1

MPNTRPVPQAKGDFLLGHLRPLAADPFRTLSTWWHDYGDAVGFRVLTRQFYMLSHPDMAEQALVRQADKFVKMYDPQKPKGLELVLGQGLVTSRGDLWRNQRRLMQPVFQRGNLSALQPQIVAAGREMLRRWHRLGDGAQVNLCSEMMRLTLEVITQTMFGTSVLDRIEAIAPALDTALRHAAASLLNPLALPPFVPTPGNRAFNRAMATLDGVVYGIIEQRRTNPAAAGGDLLDMLLQARGEDGGAGMSDRQLRDEVLTIFSAGHETTSNLLSWTLYLLARHPDALGRLRRELQDLPAEEDWQFADLQTLEYCKAVLNESLRLRPPVGVIMRKIRQNTEVEGYRLAAGSLALINIFNLHHHPQFWNDPERFEPERFLGNQSRRFAFMPFGAGERICIGNHFALLESQLLLSLIVRNYAIELLDPGKADMEMVVSLRPKGGLPVRLKRLAKP

>CYP1229A1(XCV2150)Xanthomonas_campestris_pv._Vesicatoria

MRVRCNAAAIGKKGARAARLVAQPYRLSQCSRRSQVDTRSPLVSLWLLPTVHAALSNRSGFPRSPPHEMIMQQPLKCTSAELQANPQIVFARLRPLTPVLQRDDGLYVAIRAQDVQQLLVDPRTRQMETEIATARGVTDGPLLEFLKHTMVLSNGTAHRNRRLPLVQAFASRFVQDVRPYARRVAEQLIDARYDAGAMDVIGDFASWLPARVICHILGLPETDIPAFTRCVYSVSRAFNSTFTPDEVPELQQASGELDAYVRGLIAHRRRHPREDFITSYIAASDASGQLSQTEVVAQLMSILLAGSDTTRSALAIQTSLLLQHPEQWQAVCRDSALIPAAVRECLRYQPAVASVPRITLEDIVLDDTLVPAGKILSLSTLSALRDPALYAEPERFDIHRTDAPKRQLVFGGGVHRCLGEALAMIELEEGLAAMAQRLPDMRFDGNPVVVQGGFGIRTAQDFRVSWGSARTG

>CYP1234A1(ABO_2384)Alcanivorax_borkumensis

MNRMEVAQQEAAQLPRMPQRKLKDIPGDYGWPLLGHTVPFLKDYHKMVTQQAAKHGLIFKSSVLFQHGVTLLGPDANEFVLKDPEHAFSSRAAWNPILEKLFTDGLMLRDFADHKFHRRIMQQAFKKPALASYLGRMNGHIGSEISHWPTGKELRFQDHIKSLLLDVGAQIFFGLEMGPESNKVNQSFIDATDASLAVVRLPIPGLLWHRGMKGRRYLEKFVTGLIPQKRASNTPDFFSELCKAADEEGGLSDKDVMNHMIFLLFAAHDTTTSTLCSIVYMLAKHPNWQDILVKEIEGLNKETLDYDDLAKMEKTDWVFRETLRMRPALTTFPRRTVKEIEYQGYTLPKNTLVSISTLYTHYMEDYWSNPTTFDPERFSDERAEHKKHFYQWVPFGGGHHKCLGLNFAELQTKTFLFQFLKRYRVSVKPGYELPTQQVPLIMPKDGLPVVLEKRA

>CYP1234A1(AS19_24460)Alcanivorax_sp._NBRC_101098

MNRMEVAQQEAAQLPRMPQRKLKDIPGDYGWPLLGHTVPFLKDYHKMVTQQAAKHGLIFKNSVLFQHGVTLLGPDANEFVLKDPEHAFSSRAAWNPILEKLFTDGLMLRDFADHKFHRRIMQQAFKKPALASYLGRMNGHIGSEISHWPTGKELRFQDHIKSLLLDVGAQIFFGLEMGPESNKVNQSFIDATDASLAVVRLPIPGLLWHRGMKGRRYLEKFVTGLIPQKRASNTPDFFSELCKAADEEGGLSDKDVMNHMIFLLFAAHDTTTSTLCSIVYMLAKHPNWQDILVKEIEGLNKETLDYDDLAKMEKTDWVFRETLRMRPALTTFPRRTVKEIEYQGYTLPKNTLVSISTLYTHYMEDYWSNPTTFDPERFSDERAEHKKHFYQWVPFGGGHHKCLGLNFAELQTKTFLFQFLKRYRVSVKPGYELPTQQVPLIMPKDGLPVVLEKRA

>CYP1234A3(S7S_16860)Alcanivorax_pacificus

MSASMHDAQLHAARLPRMPQRQLAHIPGDYGLPVLGNTLEFLRDFQGLVHRKAAKYGPVFRSNAFFQRSVTLLGPDANEFVLRDTDHVFSSRAAWNPMLERLFTDGLMLRDFADHKFHRRMLQQAFKKNALAGYMTRMNPRIAAGIRGWPEGHTFRFFDHIKSLLLDVGAETFLGLDMGPQAQQVNQAFVDAVEASLAVLRLPIPGTTWQRGLRGRRFLEQFMTGLIPAKRAGDGDDFFSELCRAADDEGEATLSDQDIMNHMIFLLFAAHDTTTSTLSSVIHALAQHPEWQERLAQEYFSLGTDTLAHHDLERLPQTTWVFREALRMFPPLPTIPRRTVTEVTWQGYRLPANTLVSVVPLHTHYMPEYWQHPERFDPERFSPARAEDKQHFFQWVPFGGGHHKCIGLNFAELQTKLFLFHFLRRYRVSVEQGYRMPYQLVPLAVPKDGLPVQIRRRMTA

>CYP1234A4(AZF00_06235)Zhongshania_aliphaticivorans

MKTSTDYLELPRKANRHVDHIPGDNGALPILGDTVEFLKDYHGLINRKYKLYGPIYRNNALLQRNIALLGPEANELVLKDSDKIFSSKKAWDPILDKLFPNGLMLRDFDVHRFHRKVLQAAFKKESLQAYLETMNPRMRQGVIDFPKDSEFGFKDSIKSLLLNVAAQVFMGVEMGKEADKINKGFLHAMEASMAVVKLPIPGTTWYKGIKGRQALEEFIQKHITAKRATESGDFFSQFCHAKDEDGNELSDEAVRDHIIFLLFAAHDTTTSTLCSIIYAIAKNPEWQDILHNEYQQITGDDLQYDDLPKLEKTGLVMKEALRMYPPVPVIPRRTIKETEIMGYRIPANTGVGVSPLFTHYMEEWWTEPQKFDPERFSPERAEHKRHFYQWIPFGGGHHKCIGLNFAEMQVKLFLFHLLRNHTISVKPGYKMQFNVVPIVFPTDGLPIQINKRKA

>CYP1234A5(AZF00_12795)Zhongshania_aliphaticivorans

MNSQSIDQAELPRRPNRSLSHIPGTSGLPVFGQTFSFLRNYKALTELRFKKYGKISRGNTLFQHSLTLLGPEANEVVLKNSEAQFSSMLAWNPLLDRLFPNGLMLKDAEAHRYDRKILQGAFKKSAIEGYLDTMNPQLERGLAAWPKGQEFHFQHTVKKLLLEVAAEVFLGVEMGPEASGVNQAFVDTMLASMAVVKLPIPGTLWHRGLRGRQHLEDFVMAHIDTKRREQGRDIFSQICHATDEDGNHFSDEAVRDHIIFLLFAAHDTTTSTLCSIIFALAKNPEWQQRLREEYKALGKPQLEYDDLGQLEDSKLVFKEALRMYPPVPAIPRRTLKDMELFGYHIPKNTAVAISPLFTHYMEEYWSEPTKFDPERFSKARAEDKKHFFQWVPFGGGAHKCLGLNFAEVQTKLFLFHLLTRYQIQVKEGYDMPRNWVPLIFPADGLPVTFIPL

>CYP1234B1(IMCC21906_00747)Spongiibacter_sp._IMCC21906

MAEAETEHYTLAKDNTDLAHIPGSFGPPIIGHTIALVRDLHGTISKQQQQYGAVSRFGLAGFKGVLLLGPDLSQEVLRDPQRNFSAEMGYRRSLGRYYLGSLLLRDGEEHRFQRRMMQTAFKAEAMRGYAERMGAMMASAIDSWRYTPEMKGFPAIKDILLDSAAQIFVGVDPGEAAAKNMNRAFTDVANGMLGIILKELPGTRHAKAKKQERFLQSFFNHLIDERRQGSASDVFSYLCRERTEDGAFFAKADISVQMSFLLFAAHDTTTSALSHLLYYLGQDMETQQRLRDEVMALDKSLLEYSDLEKMPLAEVALKEALRLHPSVMMMQRRSIKACELGGYHIPENTLIFLAPQHTHRMADYWDAPDKFDLDRWLAPREEHKRHSFSFVGFGGGVHKCIGMHFALMQSKIFLHQFLRRYRFKLADNFSSKMQTVPLPKPVDNLPVVLVPIKAV

>CYP1247A3(VS_1891)Vibrio_tasmaniensis

MKRIEQLPMPPTSGVLGHVSYLKQPNVHQKMLCWIKEYGAHFRLKLGLKDVLVLSDAAQIKSVLKSRPDEFRRLKSIESVFDEAGLNGIFSAEEERWKHQRKLTEPMFQPSHLKHFHPQLSVITERLGKHFEMLAESGEVVELVAEFKKYTVDVTSLLAFGEDFNSIEQTTTPLGRSLQDVFPVINQRCKSPIPLWRFFKTKKDKQFDASLEDIGHFVSGCIEKQRARLISEPNLKDSPENMLQIMLLEQEQDSSLTDQDIMANAITLLLAGEDTTANTLAWMAFLVSGNRSSELALQDELDALGKRKVLEWPLPRTPYMTAVMYEAMRLKPVAPQLYLEPTRDTQIGDIEVKKGTPVFVMLHANGFDPDLFEDPTTFNPNRWVEKDGASFSNLQPFGGGARLCPGRSLAMMEIKLAFHTLFKEFSIEPQQAPEAVVEQFAFTMSPVGFNVKITKRS

>CYP1261B1(BST95_01770)Halioglobus_japonicas

MLSRAAITHPHTLYAQLREHTPIARIGDSGFHTVATWPHIEEVLGREDDFSANLTGVLYLGDSGEPACFELPQSGSATVIATADDPRHAVHRTLLQPAFLAAQIKAMEPLLRSWASATLAPLTSEGGGDFTALAEQVPARAVAHLLGLPQEDMPLHRQWAMMGGDILAGKITSERLIYLAEQTTQMATYLAAQLDAVPNHIDAGAHSPILYSLAKAIAQETISREEAVGIAIVLFGAGGESTSALIGSALYRLAGNTELAEQLRRKPALIPLFIEEVIRLEPPFNFHYRTVRRACTLGGFDLVPGDRLMLLWAAANRDGARFVDPDTLRLDRRHSRQHLSFGRGMHFCIGAGLARLEAKIIVETVLTDPRRLELMTTPKPVYADSIFVRRFEQLPLRFAD

>CYP1311A2(IMCC21906_02744)Spongiibacter_sp._IMCC21906

MQFDPMSPDFQQNPYPFYDELREKAPVIWSESMQGFCVAGYDEIMTVLTDSDQYSSSKFWPILLGEYDPAPEVQPMISLDPPDHLRTRTLAQKAFLPRELKKLEEKIIQISDELVEHAISVSDDNTFDMAWDFAALFPVSVIAEMLGIDKSMRLDFKHWVDNLLAASNRAVYDEARLKEIKHASDSLRAYFSKIIDERTENPGDDMISAFIKAEVNGEKLSKIEVLNLSILLLIGGTETTTNLIGGLFAGVNEYPEAFAAARADRSLVPQLLEEQLRYRPPVQSLFRHTTREVVLGGVTIPENTMVMPLLGSANRDPEKFPNPGKFDLNRDVRGYCTFGQGPHFCMGSFLSKFEAAIAVNRLFDRFKVLEPLQKTAEIRWIDSYFAHGPATLPVRYELA

>CYP1415A2(CD58_14745)Pseudomonas_brassicacearum_DF41

MPNNPSAIATVKPSAAKYAQLGFLKIAESACRQHGDKVWIGEEEHAVLLLAGARHLRFFLENESSFHKELDNGASVRRLLLGQSLITAREGEEWHLARKLTTPLVNPKSALLKQGTRLSAQWLVDTLHDPEKNSMQEICLQWALMCVAQGFVGSRLGYQQLDELINHFRRIYLQLIVAEPNEDYAVLSQHPALIAFRETLESMIGPLVTGVDAGDRDVDMLVRFCQALDVSAHPQERERAISLLLGNLVASVDNTGIALLWCLAHLSQHPHYQDQVREESCQGKRDMAIAIVKESLRITPVTAFFERGTLDPLEIDGVGIPAGTKVLFSPWLVHRNAVYWPEPLSFRPERFLEGRKIPREHFVPFSMGKRNCVGMALALDQLTTAVEALCSHCRFSLAPSTTPAALTPLYGLNVMPRGPICFTIESVDRVSQHECIA

>CYP1464A1(PFL_2992)Pseudomonas_protegens_Pf-5

MSQIEDTTVPAQSTADAAQPQLKAARPLAANIKIKDGFPVMPGKIPLLGHVHKIGKDALGELRQAEAACGPMFWTYFGSQLPVLQIVDEAGLAILQNKYTDNSFLREQMPVITGEAMNAFDGPRHRNARKASTDAFTPKGLTRAKVGQFIMETIDQRLKHWSRENQLAIFPQTKDIALEVVFRILGIATHELELWRHQYEEFFLGMIPLKINLPGFPAWRCRKARGWLEQRVAQIVATTRANNDHDSLVGAMILGRDEQGNGLSEVELVHNILGLGFAGSETTAAVMAWSALMLSQHPDVWQQLCEQVAGLESLPVTHEELVRQVPLAEGIFRETMRLYPPAPFEMRKVHTEFELLGQRIPAGVMAGVSLLHVSRNPERYPDPDSWRPERWLGLDRALNQVETCQFGGGPHACLGRHVAALEITLFIAMLARELGPKGNVPRLVGKMPPPAYLPFLRPSNKAFLDFSGA

>CYP1464A1 (POS17_2973)Pseudomonas_sp._Os17

MSQIQDTRVPAPEHADATAPAIKAPRPLAAGIKLKDGFAVMPGKIPLLGHVHRIGRDALGELRQAEAACGPIFWTYFGTQLPVLQIMDEAGLAILQNKYTDNSYLREQMPVITGEAMNAFDGPRHRNARKASTDAFTPKGLTRAQVGQFIIETIEQRLKHWSRENQLAIFPQTKDIALEVVFRILGIQTHELALWRHQYEEFFLGMIPLKVNLPGFPAWRCRKARGWLEQRVAQIVATTRANNDHDSLVGAMIFGRDEHGNGMSEVELVHNILGLGFAGSETTAAVMAWSALMLSQHPDIWQALCEQAAGLDEMPVTHEELIRQVPLAEGIFRETMRLYPPAPFEMRKVHTEFELMGRRIPAGVMAGVSLLHVSRNPERYPDPDTWRPQRWLGLDRALNQVETCQFGGGPHACLGRHVAALEITLFIAMLARELGPKGIVPRLVGKMPPPAYLPFLRPSSKAFLDLSGA

>CYP1465A1(BZ13_874) Francisella_philomiragia_subsp._philomiragia_ATCC_25015_O#319L

MNKIKEKNYNIYAPFPPYYTDEKVPLRKLLKAKSFIEFYKERHYKMKMGYPKKKVGKKEISLCVNEYVLDVLSDYERYPKSKNLHKLLSPLLGNSIFTTNGDIWRFQRNIMNKSFAALQPKKTFSLMAEATLALIELIDNKSKDSNIIAIDSMMTYVTANIIFRTIFSIDYSYDNAIKLFNDFNLYQETSYLLNSPYKYILYPYLKYKQREYVRKIHNQFYPEIAKRYHTDDCSQYNDILGNLILKTDEKTGKKFSQKDLNEQICMLFLAGHETSATALTWALYLISQSEELQEDLYQEVQDSLENGEIAYSSLKSMPLMTAVFEETLRLYPPVVGLLRQSSENVVMYNKNLVKPRDEIIIPLWIQHRHTDKWYNPMEFNPYRFYNKKASNVCPVYMPFGKGDRVCIGSAFALQESLLILSTIINKYRLENLTKDVMPIGRVTLKPSEPINVRFTNR

>CYP1465A1(Fphi_1150Francisella_philomiragia_subsp._philomiragia_ATCC_25017|

MNKIKEKNYNIYAPFPPYYTDEKVPLRKLLKAKSFIEFYKERHYKMKMGYPQKKVGKKEISLCVNEYVLDVLSDYERYPKSKNLHKLLSPLLGNSIFTTNGDIWRFQRNIMNKSFAALQPKKTFSLMAEATLALIELIDNKSKDSNIIAIDSMMTYVTANIIFRTIFSIDYSYDNAIKLFNDFNLYQETSYLLNSPYKYILYPYLKYKQREYVRKIHNQFYPEIAKRYHTDDCSQYNDILGNLILKTDEKTGKKFSQKDLNEQICMLFLAGHETSATALTWALYLISQSEELQEDLYQEVQDSLENGEIAYSSLKSMPLMTAVFEETLRLYPPVVGLLRQSSENVVMYNKNLVKPRDEIIIPLWIQHRHTDKWDNPMEFNPYRFYNKKSSNVCPVYMPFGKGDRVCIGSAFALQESLLILSTIINEYRLENLTKDVMPIGRVTLKPSEPVNVRFTNR

>CYP1465A1(BF30_1394)Francisella_philomiragia_O#319-029

MNKIKEKNYNIYAPFPPYYTDEKVPLRKLLKAKSFIEFYKERHYKMKMGYPQKKVGKKEISLCVNEYVLDVLSDYERYPKSKNLHKLLSPLLGNSIFTTNGDIWRFQRNIMNKSFAALQPKKTFSLMAEATLALIELIDNKSKDSNIIAIDSMMTYVTANIIFRTIFSIDYSYDNAIKLFNDFNLYQETSYLLNSPYKYILYPYLKYKQREYVRKIHNQFYPEIAKRYHTDDCSQYNDILGNLILKTDEKTGKKFSQKDLNEQICMLFLAGHETSATALTWALYLISQSEELQEDLYQEVQDSLENGEIAYSSLKSMPLMTAVFEETLRLYPPVVGLLRQSSENVVMYNKNLVKPRDEIIIPLWIQHRHTDKWDNPMEFNPYRFYNKKSSNVCPVYMPFGKGDRVCIGSAFALQESLLILSTIINEYRLENLTKDVMPIGRVTLKPSEPVNVRFTNR

>CYP1465A1(KU46_163)Francisella_philomiragia_O#319-067

MNKIKEKNYNIYAPFPPYYTDEKVPLRKLLKAKSFIEFYKERHYKMKMGYPQKKVGKKEISLCVNEYVLDVLSDYERYPKSKNLHKLLSPLLGNSIFTTNGDIWRFQRNIMNKSFAALQPKKTFSLMAEATLALIELIDNKSKDSNIIAIDSMMTYVTANIIFRTIFSIDYSYDNAIKLFNDFNLYQETSYLLNSPYKYILYPYLKYKQREYVRKIHNQFYPEIAKRYHTDDCSQYNDILGNLILKTDEKTGKKFSQKDLNEQICMLFLAGHETSATALTWALYLISQSEELQEDLYQEVQDSLENGEIAYSSLKSMPLMTAVFEETLRLYPPVVGLLRQSSENVVMYNKNLVKPRDEIIIPLWIQHRHTDKWDNPMEFNPYRFYNKKSSNVCPVYMPFGKGDRVCIGSAFALQESLLILSTIINEYRLENLTKDVMPIGRVTLKPSEPVNVRFTNR

>CYP1466A1(YC6258_04201)Gynuella_sunshinyii

MQKTHQHDDSSLNSQFNYLFNGLFKTSIPLKNKLFLKTSIGPTPYWPLGNAHNFLGQSPYMVLKQYNDSFGNTVTFWLLKKANVLLVDPRDIQVLFEQYTPDIFKDSPKHAAGKYFRRSVFFANGSEWEKKRQHHPLSHQNIKSFFGMAQPMLRNICKHYINRINAFDEPKEIPLFDEMTRLSFEIFCQFMLGEKATYTTFDAYLTQMQDMHRRGTSLFPITGFSTARKAGYWRRKVEKTINSARNSPQKSFFSLPGWLSDQGQIDDSGFKLLRDEISTAFYAGTRNVASAVSFLIQLIAAHPDKYTQLQQTIDAFMQNHPNGYQYEDLAELEYLDCTLKETLRLYPTVPAFFREVLPGRQLELPSVVLPEKTQIFISSWVTHRNPELWPSPENFQPERFFEEPQKYTYFPFGVGRQICVGKAMTDFIVKVTVIELLSQLKFESLLPHNTSYIDNEYFSGIIIPSNGLAIKVRNLSSQQR

>CYP1467A1(UMN179_00602)Gallibacterium_anatis

MCNTSIDLISSYKEFYEKLISYDTKAKGFYFNKDRKYWVVYGYEDCSNLLSNAYVSKKRMLIPLELFDKERDLVERFLHLINKSIIFRDDKKSGVVRLIHDNYKKIDILYIIERLLGKEKIIDEQNLSKLNNYLASLLVGFESDISLTKHAENVGMLFDGRVRDKEHFISIVKSFLIIFDVFKRFYNTTDINTSDIVVTYIAAHQTTMQLIVASLYNIYKFNLSVTRENVRDIITESSRLCSPILCMGRIVTQNINYKDFIFKKGDRIMFYTGMANFDPNVFKNPFEFSLNRYEKPLSFGSGVHMCIGMGISLSFSSKCVDFICSNYSIKNVSVFELIKGVSSLGAFRFSIEVG

>CYP1468A1(Marme_4095)Marinomonas_mediterranea

MRYYEEDANVTDSPNRNAAPKLIPTIKLGSQDNHDMMWNEDFHLTITNMAKKYGAIYALETGKTTLIALNDHNAVREALVNQSDVFNIRADLEILQVAPQKHFLELEAGELWSLHRKTFATAMRDYFRDRWDTMDQWLVTEIDDIEAAWKSQGDQAVFDPNRDISIKLASFLHRVMFDRRFGEFEESVFDEKSLSWLPAGFINSTRYELMPEHNKESYYAHYGDVIEKFASNLNGLDAYVSMNVLKEKECYNKGQYRHLTDFLLNACDDIPNDVKQQVGATEKEIIIGSLTQVAGAGGGVGAFALRWMLLYLASFPEKQKQVHAELDQVIGQNETPQQSHKGDLHYTQAFIAEVLRHCSITSMPASNYAASKDTFIDGYFVAKGTPLIVNNYGMTRDEALWENPDEFIPERFLEADGTFSKKQQAKSFPFGIGQRRCLGELFGKFLINTLFTHLAHRFEFSLPNNEPINLRAISGVFLVPEKVDIKAKSRSLSDS

>CYP1469A1(LA76x_0658)Lysobacter_antibioticus

MPSPLDQAQDILSYPFAIGPLGTPPETIAWARKHRPVCPISLPSGTRAWMVTNKDDIGLVLTDKRFSRDLTYAGAPRFVGEDFTAVPGGLFNLDPPDHTRVRRVIGNFYTRSGVERFRPLVERHAAQLLDAMAEGDNPADLMQAYSTQLPLHSSCDMLQVPVDFREQYLAYFHTQTNYQATAEEVAQATAKILDFSRDIVALKRRHPGLADPIGALIEARRQGLIDEDELVGTVCYLFVTGSEPLIPPLSTGVLTLLEHRAQLQQCIDDPTLWPKAIEEVLRYHHNGVLGLPRVATEDVALKDTVIRRGEAVCATMLGVTWDPKYYRHPAKFDIHRSTDGTATFGAGPHFCLGSALVRMFLEVAYRMLFARFPRLALAVPASEIPWEENILFIRPVSLPVAW

>CYP1469A1(GLA29479_2851)Lysobacter_antibioticus_ATCC_29479

MPSPLDQAQDILSYPFAIGPLGTPPETIAWARKHRPVCPISLPSGTRAWMVTNKDDIGLVLSDKRFSRDLTYAGAPRFVGEDFTAVPGGLFNLDPPDHTRVRRVIGHFYTRSGVERFRPLVERHAAQLLDAMAEGDNPADLMQAYSTQLPLHSSCDMLQVPVDFREQYLAYFHTQTNYQATAEEVAQATAKILDFSRDIVALKRRHPGLADPIGALIEARRQGLIDEDELVGTVCYLFVTGSEPLIPPLSTGVLTLLEHRAQLQQCIDDPTLWPKAIEEVLRYHHNGVLGLPRVATEDVALKDTVIRRGEAVCATMLGVTWDPKYYRHPAKFDIHRSTDGTATFGAGPHFCLGSALVRMFLEVAYRMLFARFPRLALAVPASEIPWEENILFIRPVSLPVAW

>CYP1470A1(Fphi_0249)Francisella_philomiragia_subsp._philomiragia_ATCC_25017

MKFIPPYPKPISSSKKLYKKGLLNTLRTIFYLNKSGVHATCESNFQEDVIFDLSPPLFKIIGLKESANLILNNKEVSAKKSVLINNMLRPLLRDSIFNTNDDVWQKYRTIMSRGLNTLHTRKTFFTMLDVVKKNISKFETGKEIDIEEKMTNITADIIFNTILSSQLSSRELSEFITDFTNFQKTFIKSYKFKILGIDFLEKKLNKLGQNIRNVIDMRVSSRYESFAKDDCDDTLTQFIKASLDSESLNISKDEMVDQICMLFLAGHETSAAALSWSFYLLSQDQKIQIEVYNEIKSIIGNRDVEFEDLNKLSLTSGVFYEAMRLYPPVYILPREKSTRCPISNKTIKKEHYLINNWIIHRNSNYWENPNSFCPDRFVNKKYSEYTKEGSYLPFAKGARACIGKAFAIQEGLITLAEIIKKYKILPSDTPPKPYGSLTLRAKKGIKVTLEERNDN

>CYP1470A1_ortholog(BF30_424)Francisella_philomiragia_O#319-029

MKFIPPYPKPISSSKKLYKKGLLNTLRTIFYLNKSGVHATCESNFQEDVIFDLSPPLFKIIGLKESANLILNNKEVSAKKSVLINNMLRPLLRDSIFNTNDDVWQKYRTIMSRGLNTLHTRKTFFTMLDVVKKNISKFETGKEIDIEEKMTNITADIIFNTILSSQLSSRELSEFITDFTNFQKTFIKSYKFKILGIDFLEKKLNKLGQNIRNVIDMRVSSRYESFAKDDCDDTLTQFIKASLDSESLNISKDEMVDQICMLFLAGHETSAAALSWSFYLLSQDQKIQIEVYNEIKSIIGNRDVEFEDLNKLSLTSGVFYEAMRLYPPVYILPREKSTRCPISNKTIKKEHYLINNWIIHRNSNYWENPNSFCPDRFVNKKYSEYTKEGSYLPFAKGARACIGKAFAIQEGLITLAEIIKKYKILPSDTPPKPYGSLTLRAKKGIKVTLEERNDN

>CYP1470A1(KU46_1128)Francisella_philomiragia_O#319-067

MKFIPPYPKPISSSKKLYKKGLLNTLRTIFYLNKSGVHATCESNFQEDVIFDLSPPLFKIIGLKESANLILNNKEVSAKKSVLINNMLRPLLRDSIFNTNDDVWQKYRTIMSRGLNTLHTRKTFFTMLDVVKKNISKFETGKEIDIEEKMTNITADIIFNTILSSQLSSRELSEFITDFTNFQKTFIKSYKFKILGIDFLEKKLNKLGQNIRNVIDMRVSSRYESFAKDDCDDTLTQFIKASLDSESLNISKDEMVDQICMLFLAGHETSAAALSWSFYLLSQDQKIQIEVYNEIKSIIGNRDVEFEDLNKLSLTSGVFYEAMRLYPPVYILPREKSTRCPISNKTIKKEHYLINNWIIHRNSNYWENPNSFCPDRFVNKKYSEYTKEGSYLPFAKGARACIGKAFAIQEGLITLAEIIKKYKILPSDTPPKPYGSLTLRAKKGIKVTLEERNDN

>CYP1471A1(YC6258_00787)Gynuella_sunshinyii

MSHHYSSHNTLQNAEYTDVDLHSTEFISNPFPAYQALLQQGSIYQSPSATEYYVVRPSQVERILKDNKTFLSDRTGSFAAKLSEGQHDQVQPLLNSLAKWLLFQDPPKHMPLRKIVNASLSHKLVSSLEPDIRAITRQLVTTMVTEQHNDLVNNLSYPLPALVIARLLGVPAEDILLVKKWSDDIASFTGAQSGIDIAERARASVVEMSDYLQQLLHSTELIENTTVLGNLTQFRQQNDEFTEEDLIANCIMLLFAGHETTTCLINNLWIQLQQHDSQRLDLIRHPELISSAVEEGLRYDGAVHRLGRFIKSDTEIDGIQLQQNRMIYVLLGAANRSPELCERPDKFDIRRKPVRNFGFGFGPHLCSGAALARLEAEIATEELLKLIPEGHVMETPEYHRNLALRSVKSLKIKI

>CYP1472A1 (PSEEN5529)Pseudomonas_entomophila

MSINTAPDVEMDVLLEPVEQGAGGNRKIDGPSSILGTVRKLRKDALAALVEFNTTYGDLCRIKFGLKEQALIISHPEDIREVLSDRRGHYQKGGNRNFKEIDRFFTNSLFTSDGDFNKRQRKLLKPTFNPMLTDSFAVPMVNAAKEMMDAWEQQGLQQIDLKQAILQLTRRNICENVLGVEETFEDAARTIRECFEVANIVTMERARQIAPAPLWVPTPSNRRFLEAKERMLHLIERVIERHRVEQAPVRSMVQMFMAARYADNGEPMAHEQLLTECMTLCFGAYETSANTFTYAFHFLSKYPQVRARVIAEVDEVTQGRLPTIADVAKLGYTRKVLNETMRHYTPGSMLIRCAKQDTELAGNPVPAGTMIVLNIYFMHRHPDYWENPLAFDPDRFDAPVANPGVKQAFIPFGGGGRSCIGMGMAMMDGLLLLATVSQRYLLDNRLDAAEGAAPSLRRIVMGPESGVQVMLRKRGHH

>CYP1473A1(JT25_015675)Methylomonas_denitrificans

MANSTLQIHPEKPSLPEHSVIMPMAQLIWRPLATLARIHSKYGELVLGRLFGRKILFVSTPEHIEQIFNLEGKGLLSRDFLYDAKKVLFGDGLVNSGSEVWSRQRRLMQPLFTKEAVKNYELIMIEEAAAVANQLKKAADSPINLTTELKNLIQRIFIRILLGKSVDSLSNSAELIKVIEIIRQELPVQLGSEIIFGSRLKRFIPLKSRRYHAAVDYLKAFIRQEIAEKQENPGQDLISQLIQSGDRATGYTMPAELLQDEAVNLFFAGQETTINTLLWFFYLTGKHAEVRNKIAAEIRQLPDGPLNAAHLSQLSYTKAALNETLRLYPPTSALSTQTVQDIELGSYNIPKGTTVLLSMHTTHHNPRLWDKPEEFNPNRFLETATPERHKYAFFPFGGGVHNCIGKHFAELEMLLVIASFIREFTFETDITVKEAFSITLKPDRPIVGRVEPIS

>CYP1474A1(Smal_1573)Stenotrophomonas_maltophilia_R551-3

MDERAADAEVVSINDLLERVRREGPVLRFQNDVVGIFDPALAVKIDKANTDQHTVPDSLIDFLGLRGSKDPVAWREVRALLSEQAGRLASPAHMRSLYARMQGFLTERAGRPHDLSALSWWTISQSLLPLLIDGLSRSDVDALIGEQKTRYNAIVLQNFSFWRRVIDFHLSRRAARTVSRHIRRRSRETQPREDFLQSLLPLVQRVGVDRVAYLVSMVLAGMSGLPGITAASLLYAMHRFPQWHARIREETSALSLDELYALPIKSLPCTSRFVKETLRLWPGLFALHRPASHDIDIDGVCIRKGGAYELSSYFQHHSPDYWQSPDSFDPDRWLPERRQPNKGAYVPFGFSSRACIGSAVGHAQLLLFCALVTRDFELQVQDQPAPWMQLEGFAIPVDFIGTLTPRRA

>CYP1474A1(BurJV3_1622)Stenotrophomonas_maltophilia_JV3

MDERAADAEVVSIDDLLERVRREGPVLRFNNDVVGIFDPALAVRIDKANTDQHTVPDSLIDFLGLRSGRDPVAWREVRALLSEQAGRLASPGHMRDLYTRMHGFLAQRADRPRDLSELSWWTISQSLLPLLIDGLSRSDVDALIGEQKTRYNAIVLQNFSFWRRIIDFHLSRRAARTVSRHIRRRARGTVPREDFLQSLLPLVQRVGVDRVAYLVSMVLAGMSGLPGITAASLLYAMHRFPHWQARIREETSALSLEQLYALPIKSLPCTSRFVKETLRLWPGLFALHRPASHDIDIEGVCIRKGGAYELSSYFQHHSPEYWQNPDSFDPDRWLPERRQANKGAYVPFGFSSRACIGSAVGHAQLLLFCALVTRDFELTVQDEPTPWMQLEGFAIPVNFIGTLTPRCA

>CYP1474A1(SMD_1771)Stenotrophomonas_maltophilia_D457

MDERAADAEVVSIDDLLERVRREGPVLRFNNDVVGIFDPALAVRIDKANTDQHTVPDSLIDFLGLRSGRDPVAWREVRALLSEQAGRLASPGHMRDLYTRMHGFLAQRADRPHDLSELSWWTISQSLLPMLIDGLSRSDVDALIGEQKTRYNAIVLQNFSFWRRIIDFHLSRRAARTVSRHIRRRARQTVPREDFLQSLLPLVQRVGVDRVAYLVSMVLAGMSGLPGITAASLLYAMYRFPHWQARIREETSALSLEQLYALPIKSLPCTSRFVKETLRLWPGLFALHRPASHDIDIEGVCIRKGGAYELSSYFQHHSPEYWQNPDSFDPDRWLPERRQANKGAYVPFGFSSRACIGSAVGHAQLLLFCALVTRDFELTVQDEPTPWMQLEGFAIPVDFIGTLTPRHA

>CYP1474B1(LC55x_3050)Lysobacter_capsici

MTTQPDPADDPAARLLERVRREGPVLPLPGGVVGIFSPALANKVDKINSDDLKVIDSLADVLGMRKSEPVTWREVRALLTERSGALVTPGQMRALHQRMQAYLGEHTGSEQDLTKLMWRTVSRALIPLAIDGIDGRDLRTLIAEQELRFRIQLEQHVPLWRRIPDFLLHRAATRAITRQIKQRVARGESRDDFTQPLLGLVDRIGVDRVTYLVTVQMIAISGVPGMMAACLVYAMSQYPQWREHLQEEMDALEWEELYTLPIRKLPRTMRFIKEAMRLWTTPFVTRRVAQRDIELDGVSVRKDQIYELSSYILHHSEEYWDEPETFDPDRWLSSRRQEAKGAYVPFGFGPRSCVGASVGHAQLVLFCAMMVRDFRCDLSPVHAPWMRKEGFAVPTDFVGVVSPRPR

>CYP1474B2(GLE_2797) Lysobacter_enzymogenes_C3

MISIDHLLERVRQEGPVLDFQGDVVGIFDPVLAVKVDKANTDRLTVPDSLIDFLGLRKSRDPLAWSVVRTVLIEQSGRLSQPEHLRGLYSRMQAFLAQRAGSVRNLSELSWWTISQSLLPMLIDGLDEADTDALIGEQKARYNGIVLQNFSPWQRIVDFGRSRRAARAVSRQIRQRLRDGDRREDFLQSLLPLAERIGTDRVAYLVSVVLAGMSGLPGITAASLAYAMYRFPEWRERIRAETAALSLDALCALPMRELPCTARFIKETIRVWPGLFALRRPAFHDIEVDGIRVKKDGAYEISSYYQHHCPHYWDAPDVFDPDRWLASRRQSNKGAFVPFGFSSRACIGSSVGHAQLVLFCALLTRDFDIQVQEQPKPWMQLEGFAIPVDFTGIVTPRGAAAA

>CYP1474C1(SMD_1774)Stenotrophomonas_maltophilia_D457

MSNEGSAIAAERLAARVEREGPVLKLETGGIGVFCPELAGKVDKENSEHLFVVESLADLFRRHERVRWTEVRSLIATRSAELTGAQTLEALQSRMRQALDALCGRELDATPAVWKVMAHPLVPLVIDGLDARGTEAIERALHLRYTTQVEQVIHRRHLLRNFLIGRGESRAIAAELRRRVRSGRSPLDYAQSLLTLRERIGLDKVTYLVTVQLIAISGVPGMMAACLLLAMQMHPHWRQRIEEEFAPLSDAEIHALPMARIPCTMRFLKEVMRLYSTPFNNRRVAACDLQVEGQAIAEGTVFELSSFIQHRSAKYWDDPLQFDPDRWLPERRKRTAGIYVPYGFPTRSCVGSAVGNAQLVLLCALLAREFRFTPSADYRPEVRMEGFAIPAALHGTFSRHKAGA

>CYP1474C1(BurJV3_1626)Stenotrophomonas_maltophilia_JV3

MQGMSNEGSAIAAERLAARVEREGPVLKLETGGIGVFCPELAGKVDKENSEHLFVVESLADLFRRHERVRWTEVRSLIATRSAELTGAQTLEALQSRMRQALDALCGRELDATPAVWKVMAHPLVPLVIDGLDARGTAAIERALHLRYTTQVEQVIHRRHLLRNFLIGRGESRAIATELKRRVRDGRVPLDYAQSLLSLRERIGLDKVTYLVTVQLIAISGVPGMMAACLLLAMQMHPHWRQRIEEEFAPLSDAEIHALPMARIPCTMRFLKEVMRLYSTPFNNRRVAACDLQVEGQAIAEGTVFELSSFIQHRSAKYWDDPLQFDPDRWLPERRKRTAGIYVPYGFPTRSCVGSAVGNAQLVLLCALLAREFRFTPSAGYRSEVRMEGFAIPAALHGTFSRRTAGA

>CYP1475A1(LOKO_03351)Halomonas_chromatireducens

MTDSKPTESTPPPSLPGLPLLGNTIELLTKDANRFFSEAYEKLGPVFQVNYLFRNYTIMAGPESLNHLLEFREQGMSREAFFGPVDKQIGGVVLLTQPVGAYQNLRNQARLAFSRQLAAEFIPDLVGCVDEGLDRHPPGSTVSVMDLCTRVSFDQYSRLLCGESLDAYFDTANRYAVWVMNIGVKKVPEFSVHLPAYKKLRRDVMKMADEVLTRYENRPADHGMPYTILDALVSATDDDGNPLPRKDLVATLQYGIIGTVVYMNRTVAFLLHDLLSNPEHYERVREEVDGAYANGLPDSLDLRGMSTLNAALKESMRLHPVSLGMPFMVDEPFEFNGHTVPKGQFCVYSGVPNHFSADFYPEPHRFDPDRCRAPRNEHKQRRAYAPYGYGKRVCPAGGLVETCTLVSISRIIHRRTFERVPANDPLRTVLAPLPAPDRKFRIRFNEERSKHAAGKTDHTQLAVNALDELFESGRLAKPAMRERLDHVTARRHEPGDYILRKGERAETFHVLIEGTVEVTSGSDEHLATLEPGSYFGEIGLLGEGRRTANCLSTTRTLVLEMTREDFLAIVLKDDLVPGEIAAALRQRYLTTRMHKALPALTSEQLRTLAGTGSLKRFEADDTIIRQGDEADWFYILLTGKVAVLIEDANGTQQVATLEAGDHFGEIGIIESRARTATVQVTPDGPVETLAIQRDALLELVEHDPQARKDIASVIMDRIRTGAP

>CYP1476A1(BST95_07125)Halioglobus_japonicas

MSEAATALPPVAEIFNPHSSEYMNDPVSQCLALAERGPIVWYEPWQAWIVTRMEDIMACWKTEPLSSDFYDWEFAPERPSEDKWSNFERAMIGHSLLADHGHHRLVRKVVSPAFSRNVVDKIQEKIKPDVEKLFDELGSPETFDYIEDIAQHIPFISITRMVGIPEKYWPEIRKVILTFTETWNPTISDEQREAARQDCNKAIDIILKVIAERRAQPEQDDFLSTLLKIEEENENFKEWDIVTLVLALIGAGADTTLVAQQWSVYSLLKHKDQVAAALESPDAFSNAFSEINRWGVASKMGFARYAPNDMEFMGEQLRKGVMVLMMPHLKDYNPAYYDSPETFDVKREFNPDAMFGYGPRFCIGAALAKRQLYLTMCELFTRFPDVELAEEPERDADDHNAIVFKRLMLRTNA

>CYP1477A1(Fraau_1243)Frateuria_aurantia

MSPIQRECPCSARTDITISPHPHSVPPAKGKPAFALWLLYLAIIITLIGAWLLHPVLGIALAAGLAIAAAAWTSWHNQRQARRFRRVTGIDNTGPERLPVLGALRTFCDIALRRQTQLRDIDLQRMRRYGDIYLMFLGTMPIVVVTCSRLAGRISTALDVFAKSDPRDLNMPFYYQWVGNNNVVLANGEAWRRIRRITHPPLNRVHLFSPIFRRKALLLCESIASQLPATGTADIKLQRWLKAVSLDSAGEALFGYDFNHLKQQRNPGVDALDYILAQVFNPLRRMLPIINQLPLPSNRRLQRSMTLLDQLVLNLVRSTRNRQSETPRNHVLELLLREHAEEHLSDEELRNNIIAMVLASHETTQVALGGVLYHLARYPQWQQQIRQEATELFPDLDAAFAQPGHHEDGRHSLAFRSLQSFHTLSRFILESMRIYSPLAHQNPRTTTRATELAGYQIPIGTLVSINIHAIHMNPREWTQPERFDPERFTGESSQFSHAYLPFGTGPRICAGRLFSLMEQKIVICHLLRRFDIELPSPDYILPLERGSFTGMHDASFRLRFRQRTSPAD

>CYP1478A1(YC6258_03069)Gynuella_sunshinyii

MTKKTSNSDITYNGKPLQGLALSSEDEVFRENTDEVLDYIRKTVPVLEDGEFKRWFISDHAVADGVLKDRENFRRDWEENAAEGTWGKDVAPMIGKWGMFDIDGPEHKRVRAPVSRMFTPGKLKLLIPKIEEAIDREIEHIRNKEEFDFISEFAVPMSTNFMAALLGIEARDLVEFKQYAEDLTQVFIERESENVIEKAGAAKEGLEAFFHKVVAEKRENPKEDLISLLTEEEKNPITDKDIVDNCVLMVIAGKVTTTDLLGNGLVAFLQHPEQLKEVQENPELYDAAIEEILRYDSPATEIPRYNRNACQLKDVHLEQGQTLAISLAGANHDPEVYSCPHQFNIHRDEPAHLSFGGGAHYCVGAPLARIESKIAFKRFFDEFPNAKLVDEKPKRKAIGGFGGYREIFVKIN

>CYP1479A1(HCH_03600)Hahella_chejuensis

MRPPTVQSQELALKLLSHKKLAAFSIEDYVEKLHQESGEDFSYLIQVTRAMLSFMEGASHLALKKVALSSLQHGLIIMDKLCLHAVTRERLQQLKLQSQVDLVNDLCDPLFTDMISILFGLDIPDRADFLALIDKAAAITEPLLPIRRLKEVQNAFLTLRDMIGGQWRTLRPGILSAMQQHGDGLGQEELLILMATLVIASRTTTETLAGVMLENSGKDGCRHGLMGDPQWVEEHIEGMIRLCASTEYLTRVAKESVQIGDLPLAAAGQVFIHAPSANRDPSYYPDNHFSGLERSKHCRHIAFGGGSHRCPGANLAKVTLSAVIPIIYSTLERIDIDPKAVRYKNSTFAKRPASIPAYVC

>CYP1480A1(TERTU_2285)Teredinibacter_turnerae

MSNDFLLPANIKFKTFSLGFRNDPFNTYKTLLSDAPIFYVKGLHGKEWVVAKYEYVSSALKDRSLVKPDVVNEIAGREKNNKNEKKYLYLKSMMSNWLFFMEADRHEKIRKIMAAWFTPSKVSEINKILLKSIEDNLSTLKAEHSFDVLGDLSTKVTLTTISHLLDCARMDNRETEKYALELFKIVNPPVPVNEYDSLEQAARYFAISLREDLDREHSKQGFVQYLLKKKSCADITEDEIIGALSLVMCVGLDTTKHLIGNSLQALYSDGKQLQAIRRDTSLLNQSILECGRYNAPVSMLPRVAIEDTYIGDAKIAAGERVYFLVSAACRDGERFSEPDSLNIHRSKRSTLIFGAGHHFCLGAHLSLVIAEQVIKRLITSPDLAIHFKDFSWVSSPGIRGLERVQASWVS

>CYP1481A1(PAM18_5672)Pseudomonas_aeruginosa_M18

MNSKENSAPFPGLIAHHSGSCPATGHAEAFHHATVCQGLDASTYFARSGIAELAENNEGLCTFWLGDDLALYQTTNAPLVDDEDLAPSINANAELFGSFLGSLPAQDERRKAKRAVVERVLGSNRFVTSLDPHVREMAQHYLREVAGRSLPLQDFCLHMVARIDSGLPGVLDFHQKPLTHYLQSTEYGVIARDFFEIASEVISKMNPESIENADMIVEMTRDMLDSNYESIVRAPPTNMILAQFDCFSRPFTRETIRTLDAASLKELGTIIVATYDTTALSLLWTLTYLEDNPAEKERLLGVVDNPEQALDEAYLLVLEAIRLGGSNPTALWRRTNRPIRIRHRGTEVTIPANTMLWLDRRRANRDASLFPHAERFDTDNIRQLIRNQTSHGQAVSLLARNRYEINSFNMVNTHRSPRKCPGRLFSVREQALILTELYRLYKVCVTEADSTLAPHSSMPRPRRSGNIILTARAAAL

>CYP1481A1(AI22_04035)Pseudomonas_aeruginosa_YL84

MNSKENSAPFPGLIAHHSGSCPATGHAEAFHHATVCQGLDASTYFARSGIAELAENNEGLCTFWLGDDLALYQTTNAPLVDDEDLAPSINANAELFGSFLGSLPAQDERRKAKRAVVERVLGSNRFVTSLDPHVREMAQHYLREVAGRSLPLQDFCLHMVARIDSGLPGVLDFHQKPLTHYLQSTEYGVIARDFFEIASEVISKMNPESIENADMIVEMTRDMLDSNYESIVRAPPTNMILAQFDCFSRPFTRETIRTLDAASLKELGTIIVATYDTTALSLLWTLTYLEDNPAEKERLLGVVDNPEQALDEAYLLVLEAIRLGGSNPTALWRRTNRPIRIRHRGTEVTIPANTMLWLDRRRANRDASLFPHAERFDTDNIRQLIRNQTSHGQAVSLLARNRYEINSFNMVNTHRSPRKCPGRLFSVREQALILTELYRLYKVCVTEADSTLAPHSSMPRPRRSGNIILTARAAAL

>CYP1482A1(Loa_00739)Legionella_oakridgensis

MRKNLSRFLVPSRLVNPIITEIESNMDAATDIQIRHAVCGIVRAVMVGNILGVKQLPTNTYDLMEAYRNDVKRWGAFPFPELLNLMPSLRKKRDVYRAFSRGILEQEFEKLVEVLHTDDHPENANLIAAAVVSLFRDEHPSLSVEELSSAIKSLSVDEIRRYFENPVVQSLPMILKAADNLTDAIVLCLEQIVLDPSKFQMLRDEIDGSGLVIGDGMDIGLLKSLPILNAFYKEAVRFDAPVAVPRYAQSGYSSDAMTIPPNTMIIFDLHALAKGEQYWTNPEEFDPKRFLPSGSEASRTTGQFPFVPFSVGLRNCPAFAVTEVLFKAAIAKFVSGYELRFVEKRDNDSIVHVTPREESLTLAV

>CYP1483A1P(HCH_03601)Hahella_chejuensis

MVLSDKAFHVPDLPGFLRKLETHATVDLRYLKLYVDNSPFFLEGEKHKQLRDICLRYLSGQGLKELDDVISSQTGVILESLPSAPFDAITLIGKPVFTLIIKPILGLRPAESDKFDRLAMVLQRLIEPMLSLNNLARINSELEWLTHQIQRQFEKEPSPGGVLARLMADEETPLSGEEKIALVITLYAAVAPLAQTLCNMIDVLYRNGAPAPCEPAQLLEQLPFYIHQGAAPRFIHRVASESRRIGGVAIRQGDTVMIDIARAALTEAGPPGQRLRHYSFGHGAHFCIGAPLSKRIVSEFIPRFFQQFPSLRVIEKQYDENNHIARALTSLIVSPDP

>CYP2242A2 (CT573326) Pseudomonas entomophila

MEALDNRSLHTHGGHPFTMVAATPLQSRLHRLLGVLAAPWLLPRR WRNSRALRDRTRPYHYIGKLARHADGSWRRYLRLPTFAGDYSVLIGVEEIRGLMQHPR

GQGELVGDGRQFLVIADALGRMRLSKERQDAKQKRNIIAHLVSGPERFIQTMRQLSEQR

VRRWWQGAPTLLVNAELSEFTAEVYLRSVMDLQGPVEGVGQMLEEQVDLLGQAF

AHFNRRFDELKRQLVARIGNDPGLLNSTDYTHRLNAYIDQHYQSLQDEAFATGLNGAV

AGYVAPFPSFLALVDELGRHPRYRQALRQELLTKGDDHAAYIRRDDTLLHACVHEVLRL

HPAQPFLFRAASRDLMVNGHFVRQGSELVADIYHVLRLPELWGEDADAFRPERFQEAPE

RYRQPFLAYSSGPNNCTGQMFSRYSLKVLLAEMVRAGDWESTDEPLEHHFHFALAMSRP

VRIRLKEHHHG

>CYP159B4 (CP010896) Pseudomonas simiae PCL1751

MKQGCLVTNNSEVNVAPPTPLIIPDIQLPFSNPTPRSDHDVLRE VLAWAQRYGLIGRRGAHRLSTTALLDLGMALCGQAPTQQAETLVCWYLWALILDDRIDD

GPWAENGVLERFVTAVQAITENDGADPLDEIGRFDDPMLGVLIDDLWPRTRNWGNERWR

HRLVQNLLRHLRAQATLVNMRETGAALTLSEYLPLRRDSFGALFFFDLIDAAETLDPYQ

HSADIEWWSRLREHAADIITWTNDIHSIAKDVVCGERFNLVSILADSAGTDWPASIEAA

HQMVNAAVSAFTELAAKHTRQRPSAATDPDRLRQVARAAGDWHRSVSRYHLQANDPTGR

INQQVDLNLTPPTLKSRQFEIDPYPLYERLRTTLPIVYDEPTDVWLVSRYADVKAALTH PGASSNNYSWQIGPLLGHTLVAMDGCEHAQHRALLSPSFRSKALEVLEASITSASMDLL

AQMQGRHRVDLIADFTCALPVRVMARALGLPAQTTEEVEQLKKWCAIGFAYMGNYRQ TLLTGGLSNRDRFYDFIQPHIDARRTVPTDDLISHLLAARIDGQPLPEAFVRAYCA TAGSETSHGALANLIVNLLDEPGVKEAVMANPDLMDNALAETLRRNPPLQLVLREARES LELPSGTIPCGATFACLIGSANRDPDHFTDPDTFNMSRPHQETNHFAFGAGRHFCLG LARMEITIGARLLLQTFPGVRWAPGFQPAEHGFLNRCPDRLEVAL

>CYP163K1 (BX470251)Photorhabdus luminescens

MNLISNVAKVPDNINLTDPLTHLRPDIDNIWRQLRNESPVAWHPA

VNGQSGFWVVSTHELAMRVYRDSQTFTSVRGNVMATLLHDGDTAGGRMLAVSDGDRH IRKELLKSFSPKNLMSVQKRIKNAMHELVRNAVTSGQCDFATDVAPHIPLAAICDILKV PESDRAKLFVNASAALASNSLSVDKVDTRLARNEVLMYFYKCIQTRKIAPLEDDLISNL

IAMTQNSLALTEEELVFNCYSVLLGGDETTRLALIGIIKAFAEYPECWAQLRRGEAEI KAVEELLRWTTPALHGGRTATVDVELGGQQIRAGDIVIVWNRSANFDETVFTKPNQLDL

NRSDNRHISFSYGAHFCLGAALARIEIIALLEALVELVDQISLSGEPSPIYSTFLSGYH

RLPVKLTAAHYS

>CYP1779A1 (CP014544)Zhongshania aliphaticivorans

MLKYIRAKFRPANISHTAAPPTLAVIDPYSQAFIDSPYSALEQLR

HHSPVHRCTSGSWLLSRHRDISAALADPRLSNTPSDYAVVNQRHRDRYTCADVANNTLP

FMDAPQHTEARRNIARVFHEQLRRASLQSNLPTDTPSDLLHDFATPLCTKTLCSLFGI PRQQTAELDRQLKLWANWFFSLFSAIPSQQHRQQLDAELHSFRQFCSGLLNQKRQHPSD DLPSALAQLHDQTPSISDTFMADNCMLLLADGLNADYAIANALHCLLQQPEKINQLRAK

PELIPAAADELLRFDSPVLFIARRALEDIQLHDQLITENSGVLLMLAAANRDPDVFT NTLNFEREAKPYLSFGRGQHGCIGRVLVKQLLELSLRWLISEAPNFALVRTQPLWQHQA

GHRWLQDLPVKIP

**P450 fragments**

>Xanthomonas_translucens (XOC_0082)MAEMRVVVDQALCATTGQCALTLPAVFRQRVSDGVAEVCVAEVPPALHAAARLAASQCPVAAIRIIDADADAAGTGGGPASSQAEPSIASAPRNSGGHDGTM

**P450 false positive hits**

> Pseudomonas_aeruginosa_PA1R_23(PA1R_gp4505)

MTPQQLTEEYIFAHDLREASAKIYRAATKALLKHFGPTATVHDVDHRSVLGWRRKVLEQGLSKRSWNTYSNHLRTIWGYAIEHELVTHSQVNPFRKTTVIPPRRASKTVAAEAILRARNWLNMQVGAERCTGDRARITPAWFWLCTFEVFYYTGIRLNALLCIRKRDIDWDNQLILIRGETEKTHKEFVVPITEGLVPHLSRLLQEADRAGFADDDQLFNVNRFSPHYKSKVMNSDQVEAMYRKLTEKVGVRMTPHRFRHTLATDLMKAPERNIHLTKCLLNHSNIQTTMSYIEADYDHMRAVLHARSLAQGALENVRKVDYSGSPQASAKPKPCGQPLARMGEVPPPEARTEPAEPREHIPGTGIQGGPTVREEALPQPPDTFDQSVLFTLMAQHLSNRAASASAAPAATSGSGGSGGWGSTARSSLA

>Pseudomonas_aeruginosa_PA1R_42(PA1R_gp4503)

MNQKKAVILLSGGLDSATVVAMAKADGYACYTMSFDYGQRHRAELQAAERVARQLGVIEHKVIGLDLNGMGGSALTDESIAVPESPSEGIPVTYVPARNTVFLSLALGWAEVLDARDIFIGVNAVDYSGYPDCRPEFVEAFERMANLATKAGVEGNGFRIQAPLQYLSKAQIIQAGVARGVDYGLTVSCYQADEQGRACGKCDSCRLRADGFAAAGISDPTPYF

>Pseudomonas_aeruginosa_PA1R_39(PA1R_gp4509)

MAKYRISHDAQADIIDILRFTHNRFGDAARRRYQALIGAALEAVATDPQQVGSISREELGAGLRSIHLVYCRSMPNIGKVVRPRHFVFFRVATDQVLEVVRVLHDSMDLDHHLPQR

>Pseudomonas_aeruginosa_PA1R_26(PA1R_gp4501)

MPKHLRVLTFLALSLPLAAWAEVPVYDGVAANNGGNVPPSGYGTAGAGGAFAGGGVTTPTSVQGELFMQLQQMQDELARLRGTLEEQQNQIQQLKQESLERYQDLDRRISGGGAPAAQNSAPAGAINANGAPAAPAGNNAPAPSSEPGDPAKEKLYYDAAFDLIKSKDFDKASQAFNAFLRKYPNSQYSGNAQYWLGEVNLAKGDLQGAGQAFARVSQSYPSSQKVPDSLYKLADVERRLGNNDKAKGILQQVISQYPGTSAAQLAQRDLKNLR

>Pseudomonas_aeruginosa_PA1R_42(PA1R_gp4502)

MQQTLRITEIFYSLQGETRTAGLPTVFVRLTGCPLRCHYCDTAYAFSGGDVVSLDAIFERVAAYKPRYICVTGGEPLAQPNCISLLERLCDAGYEVSLETSGALDVSRVDPRVSKVLDLKTPGSGEVGRNRYENIPLLTDNDQVKFVVCSREDYDWAVSKLIEYRLDQRAGEVLFSPSHHQVSARELADWIVADNLPVRLQLQLHKILWNDEPGH

>Pseudomonas_aeruginosa_PA1R_24(PA1R_gp4507)

MHLVRVRVRNFRGIAYGEVHLNGHTAFIGDNNAGKSTLLEAVDLVLGPERLSRRPVIDEHDFYAGTYVDPAKNEVVPIQVEVIVGGLSDEQLRHFRDHIEWWDTQTKSLLVGAPPEGTDAPHVGAAIRVFFNGWYDVDEDDFAGDTYYATPEMPDGSYPRFSAPDKRKCGFLYLRTLRTGARALSLERGSLLDVILRLKETRLTMWEDLLDQLRALPVGETEDIGELLVAVQDAVRHYVPSDWAEQPHMRVSDLTRDMLRRTLTVFMGTGAKRPDGSVYSAPYQHQGTGTINTLVLALLSIIAELKQSVIFAMEEPEIALPPHTQKRIINSLRQKSAQAIFTSHSPYVLEEFEPAQVVVLKRTAGVMTGVPATYPPAVKPKAYRTEFKARFCEALLARYVLVLEGRTEFDALPAAARRLAELDPTRFKSLENLGVAIVDARGETNVAPLGAFFRSLGKVVFAVFDKQTPEALATITASVDHAYESATKGFENLVLYGASEVALRSYAAVVVVGGDWPQHLAAYTPTPATPLANLQVALSHYFGWAKGGGDAGDLLASCPTANDMPEYVRTTLAAIKNVIEPPQPPPLPHQLAGVPPLPEAAAKPPPLAAAYGKFHPSPPQPLA

>Pseudomonas_aeruginosa_PA1R_36(PA1R_gp4500)

MMEMLKFGKFAALALAMAVAVGCSSKGGDASGEGANGGVDPNAGYGANSGAVDGSLSDEAALRAITTFYFEYDSSDLKPEAMRALDVHAKDLKGSGQRVVLEGHTDERGTREYNMALGERRAKAVQRYLVLQGVSPAQLELVSYGKERPVATGHDEQSWAQNRRVELKK

>Pseudomonas_aeruginosa_PA1R_25(PA1R_gp4508)

MTEVWSDQKRGFLACAGHTLALGGPGAGKTHVALVKARDEIRSGVLKPGQKILFLSFARPTVARIIEKASELISREDLKQLEVSTYHGFAWSILRSHAYLLNGRPSLQLLPPPEAAAHLADIDKAQHENEKRRLFEHEGRLHFDLFASLVSELLSRSDRLSAIFSDAYPIIILDEFQDTNCDEWALIQQLGKRSRLIALADPEQRIYEFRGADPRRVGDFLELFGAVHFDFAGENHRSSGTDITTYGNDLLTGANKGKVYQQVKITRYGFMYGKSLHFTAKAAVLSALDRLKAIPDKSIAILVPSKRLMLELSDYLSSAADGLPELHHDVAMDAEPPALAAGVIATLLEGGTAGDLASRMLGALHSHIRGRRGGKPTPQSELDLAGALSGFLSSGKIRGAKRQLIVSEVQRIAELRQQLQLTGDPAEDWLQLRGLLQSSAAVALKQVATDARYLRLLHRGSVLRANLGALWRAQGEYKGAEEAVRSALMQEHFAAAQKDWRGIHLMTIHKSKGKEFDEVIIYDGLFQRIAKAPQDPKICAQDLLVLRVGVTRAIRRTTILTPKRDTCPFL

>Pseudomonas_aeruginosa_PA1R_46(PA1R_gp4506)

MELLGTPRRRQLLENIWQRASLSKQQFEEIYRRPLANYAELVQQLPASENHHHAHPGGMIDHGLEIVAYALKVRQTYLLPIGAAPESQSAQAEAWSAAAAYGALAHDIGKIVVDLQVELQDGSTWHPWNGPINQPYRFKYVKSREYQLHGAASALLIHQLLPRTALDWLSRFPELWAQLIYLFAGQYEHAGILGEIIVKADQASVAQELGGNPDRALAAPKQSLQRQLADGLRFLVKDKFKLNQPGGPSDGWLTQDALWLVSKPAADQLRAYLLAQGIEGVPSSNSTFFNMLQDQAVIQTNAEDKAIWTATIDNGAGWRNKFTLLKIAPALIWADPAERPDSYSGSLVIEEGNASPEKPETTCEIPNDPIEQRQAPEAKMTLRQPTPSVAKPSNEMRAIAKPSAEDQEETDDLYALLGNINSPPEELDTSHDSPAASSTNTRGEENLQQPLGTKEPTDCAPEAVEDVFMPSRSTDLGQGFVGWMKSGIAARRLFINDTKALVHTVDGTAMLVTPGIFKRYVQEHPELEKLAQAKETTGWKLVQRAFEKQGLHRKTSKSLNIWTIKVSGPRKTKELKAYLLQDPKLLFPEQPLDNPSLTVITDAEGGVE

>Pseudomonas_pseudoalcaligenes_26(BN5_1177)

MKVAILSGSVYGTAEEVARHAERQLKAAGLDAWHKANVSLEELLAFAPDAFLTVTSTTGMGELPDNLLPLYSEIRDRLPAWSGKPAAVLALGDSSYDTFCGGGELMRELYAELGLREVVEMLRLDSSETVTPETDAEPWLQAFVAALKA
